# Integrative Analysis of Axolotl Gene Expression Data from Regenerative and Wound Healing Limb Tissues

**DOI:** 10.1038/s41598-019-56829-6

**Published:** 2019-12-30

**Authors:** Mustafa Sibai, Cüneyd Parlayan, Pelin Tuğlu, Gürkan Öztürk, Turan Demircan

**Affiliations:** 1 Graduate School of Engineering and Natural Sciences, Istanbul Medipol University, Istanbul, Turkey; 2 Regenerative and Restorative Medicine Research Center, REMER, Istanbul Medipol University, Istanbul, Turkey; 3 Department of Biomedical Engineering, Faculty of Engineering, İstanbul Medipol University, Istanbul, Turkey; 4 Department of Physiology, International School of Medicine, İstanbul Medipol University, Istanbul, Turkey; 5 Department of Medical Biology, School of Medicine, Mugla Sitki Kocman University, Mugla, Turkey

**Keywords:** Computational biology and bioinformatics, Data integration

## Abstract

Axolotl (*Ambystoma mexicanum*) is a urodele amphibian endowed with remarkable regenerative capacities manifested in scarless wound healing and restoration of amputated limbs, which makes it a powerful experimental model for regenerative biology and medicine. Previous studies have utilized microarrays and RNA-Seq technologies for detecting differentially expressed (DE) genes in different phases of the axolotl limb regeneration. However, sufficient consistency may be lacking due to statistical limitations arising from intra-laboratory analyses. This study aims to bridge such gaps by performing an integrative analysis of publicly available microarray and RNA-Seq data from axolotl limb samples having comparable study designs using the “merging” method. A total of 351 genes were found DE in regenerative samples compared to the control in data of both technologies, showing an adjusted p-value < 0.01 and log fold change magnitudes >1. Downstream analyses illustrated consistent correlations of the directionality of DE genes within and between data of both technologies, as well as concordance with the literature on regeneration related biological processes. qRT-PCR analysis validated the observed expression level differences of five of the top DE genes. Future studies may benefit from the utilized concept and approach for enhanced statistical power and robust discovery of biomarkers of regeneration.

## Introduction

Axolotl is a salamander species of amphibians which has recently been established as a promising vertebrate model system for developmental and regenerative biology due to its unique features of high regenerative capacity, scarless wound healing, and low cancer incidence^1,2^. Due to their inability to undergo natural metamorphosis, axolotls keep on exhibiting embryonic-like cell characteristics, which promotes the finely-tuned regenerative capacity of their body parts throughout their lifespan^1,3,4^. Axolotls can faithfully regenerate several organs besides their limbs^5–11^. Studies have suggested that the successive regenerative capacity of axolotls may be driven by a weak inflammatory response due to their simpler adaptive immune system^1,12^. In response to experimental induction of metamorphosis via thyroid hormone administration, diminished regenerative power of axolotls is observed for some body parts such as appendages^13,14^, while such regenerative potential seems to be unobstructed for other parts of the body^1,15^.

The process of axolotl limb wound healing and regeneration involves several key genes^16–22^ and proceeds in several stages. In response to an amputation, a thin wound epidermis forms around the severed stump within 24 hours due to the migration of a collection of epidermal cells to the amputation site^23,24^. Within 48 hours thereafter, the wound area is infiltrated by macrophages where they phagocyte the debris of dead cells and clear the wound zone from different kinds of pathogens^25^. In the following couple of days, several key processes such as activation of progenitor cells as well as dedifferentiation of terminally differentiated cells take place as a result of secretion of mitogens and growth factors from the epidermis accompanied by innervation^24^. These processes lead to the formation of the blastema cells^24,26^. They are encoded with precise positional information, behave as autonomous units, and exhibit unidirectional signaling driven by factors originating from wound epidermis^24^. After blastema cells reach a definitive size, they flatten out for cartilage to condense, allowing the differentiation of the required tissue types, and ultimately constructing a delicately-sized, perfectly regenerated limb that is identical to the amputated one^24^.

The ease of breeding axolotls and the application of molecular genetics on them have made it advantageous to use axolotl as a model organism^24,27–32^. While the genome of the axolotl is a simple diploid with 14 pairs of chromosomes, its enormous, highly repetitive sequence and long introns^33^ have been the major hurdles towards obtaining a full genome assembly due to the inability of acquiring sufficient read-length and the absence of an improved methodology of genome assembly^24^. Consequently, only very recently sequencing and assembly of axolotl genome was reported^34,35^. Therefore, proteomics, transcriptomics (RNA-Seq), and microarrays have been the primary, indispensable tools for investigating gene networks and pathways of axolotl’s regenerative mechanisms^19,24,36–40^. Despite these advancements, a strong and popular methodology that has the potential for enhancing our current knowledge on limb regeneration is missing in the axolotl literature. Integrative data analysis (IDA) is a key methodology that is applied across many scientific disciplines and aims to derive scientific consensus on a particular research question^41–43^. Although the concept of IDA has recently been expanded to refer to experiments aiming to integrate information from several layers of “omics” information (aka multi-omics)^44^, the utilized IDA in this study refers to the process of combining information from different platforms across independent studies^43^. The latter IDA concept is commonly used in biomedical sciences to detect DE genes for having a better gene signature for basic science and clinical applications^43,45,46^.

Factors such as directly-incomparable experimental techniques between different studies due to poorly articulated experimental designs, as well as data deposition in public databases with no relevant publications are the main reasons why many biological research and preclinical medical sciences have not embraced the application of IDA very quickly^41^. Therefore, when such factors are no longer an obstacle, the advantages of implementing IDA can be realized from the limitations of individual studies. Firstly, the cost of utilizing new technologies often times leads to the collection of a limited number of replicates^41^. Moreover, researchers are faced with challenging statistical issues arising from such limited replicates, manifested in high false-positive and false-negative observations^41,47^. Therefore, IDA is useful to minimize the effects from the issues described before, especially through promoting statistical power^42,43,48–50^. Meta-analysis and merging are two fundamental approaches to perform IDA^43^. While the former integrates statistics from different studies at a “late stage”, the latter integrates data before running the statistical test^43^. It has been argued that whenever datasets are selected for answering particularized questions and are reasonably homogenous, the merging method outperforms meta-analysis for biomarker discovery analyses^43,51^.

In this study, we aimed to identify candidate genes for possibly characterizing biomarkers of regeneration by separately implementing integrative analysis on publicly available microarray and RNA-Seq axolotl data using the merging methodology. We report 351 DE genes (adjusted p-value < 0.01, |logFC| > 1), commonly identified by microarray and RNA-Seq data analyses in the regenerative phase compared to the control (intact) limb, including 23 DE genes uniquely identified by this study. Overall, this paper identifies a set of commonly found differentially expressed genes curated from various studies. Our approach would benefit the regeneration community, offering a closer look at the DE genes that are present in up-to-date gene expression studies.

## Methods

### Gene-expression data collection

Microarray and RNA-Seq axolotl data were collected from the Gene Expression Omnibus (GEO) and the European Nucleotide Archive (ENA) databases^52–54^. The collected data were subjected to selective criteria filtering which was set according to *PRISMA* guidelines^55^, based on which GEO series were collected as follows:

1. GEO series data deposited until September 2018.
2. Non-redundant series.
3. Series pertinent to Axolotl tissues.
4. Series having unduplicated datasets.

**Figure 1:**
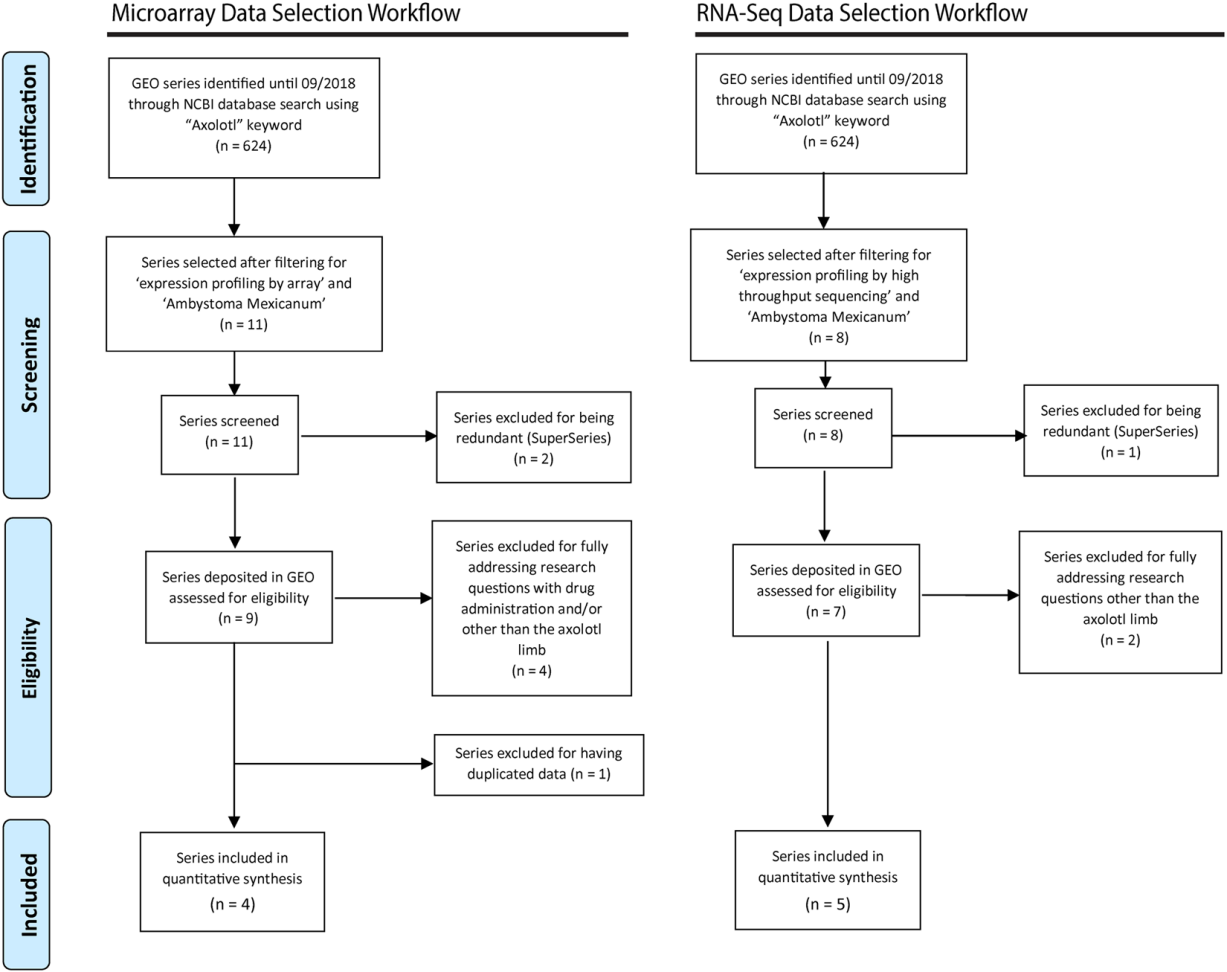
Flow diagram of the IDA design. This diagram illustrates the selection criteria used for separately performing integrative analysis on microarray and RNA-seq data from axolotl samples. The diagram is prepared according to *Preferred Reporting Items for Systematic Reviews and Meta-Analyses (PRISMA).*

The GEO datasets used, and the number of samples that are associated with these datasets, are in Table 1 and Table 2 for the Microarray and RNA-Seq experiments, respectively. Three biological groups were set for our integrative data analysis; “control” group (intact/amputated/injured limbs and/or flank wounds at 0-hour timepoints), “wound healing” group (amputated/injured limbs and/or flank wounds up to about 50-hours post amputation/injury), and “regenerative” group (amputated/injured limbs and/or flank wounds of time points ranging from about 50 hours to 28 days post amputations (dpa)/injuries(dpi)). According to the criteria for microarray data selection (Fig. 1), a total of 4 GSE datasets (series) were selected; three of which were based on the Affymetrix *Ambystoma* platform, and the fourth (GSE36452) based on the Agilent *Ambystoma* platform. The excluded samples out of the selected microarray datasets were those of denervated limbs (in GSE37198) which can’t regenerate and those of limb buds (in GSE36451) that are totally distinct from a fully mature, amputated limb. Overall, a total of 313 samples were selected (Table 1)^18,56–58^.

**Table 1:** The utilized microarray datasets.

Dataset GSE Accession	Platform	Group type			Total	Ref
		Control (0 dpa/dpi)	Wound Healing (up to ~ 50 hpa/hpi)	Regenerative (>~ 50 hpa/hpi until 28 dpa/dpi)		
GSE116615	GPL25286 [AMBY_002a520748F] Affymetrix Ambystoma mexicanum	3	3	N/A	6	^56^
GSE67118	GPL15153 Affymetrix Ambystoma mexicanum AMBY_002 20k array [CDF: AMBY_002a520748F]	10	40	148	198	^18^
GSE37198	GPL15153 Affymetrix Ambystoma mexicanum AMBY_002 20k array [CDF: AMBY_002a520748F]	8	8	16	32	^57^
GSE36451	GPL15342 Agilent-019788 Ambystoma mexicanum 44k_v3_20080327	5	42	30	77	^58^
Total	26	93	194	313

**Table 2:** The utilized RNA-Seq datasets.

Dataset GSE Accession	Platform	Group type			Total	Ref
		Control (0 dpa/dpi)	Wound Healing (up to ~ 50 hpa/hpi)	Regenerative (>~ 50 hpa/hpi until 28 dpa/dpi)		
GSE116777	GPL21473 Illumina HiSeq. 2000 (Ambystoma mexicanum)	2	3	N/A	5	^56^
GSE103087	GPL22800 Illumina HiSeq. 2500 (Ambystoma mexicanum)	N/A	N/A	4	4	^59^
GSE92429	GPL22800 Illumina HiSeq. 2500 (Ambystoma mexicanum)	3	N/A	4	7	^19^
GSE74372	GPL14997 Illumina Genome Analyzer IIx (Ambystoma mexicanum)	1	N/A	3	4	^39^
GSE34394	GPL14997 Illumina Genome Analyzer IIx (Ambystoma mexicanum)	1	4	7	12	^60^
Total	7	7	18	32

As for the criteria for RNA-Seq data selection (Fig. 1), a total of 5 GSE datasets (series) were selected, all of which were based on slightly different versions of the Illumina *Ambystoma mexicanum* platform. The excluded samples out of the selected RNA-Seq datasets were those identified as an outlier (in GSE116777) by the authors^56^, those which underwent several rounds of amputation-regeneration (in GSE103087), those which were prepared for small RNA (sRNA) profiling experiments (in GSE74372), those which were mouse samples (in GSE34394), and those extracted from multiple positions along the axolotl limb except for those of the upper-arm to be used in the control group as well as the proximal and distal blastemas to be used in the regenerative groups (in GSE92429). Overall, a total of 32 samples were selected (Table 2)^19,39,56,59,60^. Further detailed information about the selected microarray and RNA-Seq studies are summarized in Supplementary Tables 1–4.

### Gene-expression data processing

The full axolotl microarray data processing workflow can be found in Supplementary Fig. 1. CEL files from the 3 Affymetrix datasets were processed as a single dataset having a total of 236 samples and 20080 probesets. Summarization, quantile-normalization, and log2-transformation were applied on this dataset by using the RMA algorithm from the Bioconductor’s “affy” package in R (R version: 3.5.1 was used throughout the whole IDA)^61–65^. Samples from the Agilent dataset were collected from GEO as summarized, quantile-normalized, and log2-transformed, having a total of 77 samples and 43,796 probesets. In the Affymetrix and Agilent datasets, low-expression probesets were filtered out using Bioconductor’s “Biobase” package in R^62^. Probesets with intensities greater than a median intensity of “4” in at least the number of samples in the smallest group were kept; this left 17,658 and 41,579 probesets for the Affymetrix and Agilent datasets that were considered for downstream anlaysis. The former probesets were annotated using AMBY_002a520748F Affymetrix probeset annotation file (~20k probesets) provided by Sal-Site (http://www.ambystoma.org/genome-resources/20-gene-expression) and the latter probesets were annotated using GPL15342 Agilent annotation file obtained from GEO (https://www.ncbi.nlm.nih.gov/geo/query/ acc.cgi?acc = GPL15342). The annotation yielded 13,316 Affymetrix and 21,419 Agilent probeset-gene mappings including duplicates, respectively. The “WGCNA” R package^66^ was used to remove those duplicates by respectively collapsing the Affymetrix and Agilent data from probeset-level to gene-level while taking the maximum row mean value of the duplicated probesets to represent the corresponding gene, yielding a total of 10,442 genes in Affymetrix and 5,083 genes in Agilent. The different number of genes observed between the two platforms may be ascribed to the probesets design differences and the subsequent elimination of some probesets through the filtering steps on each platform. The latter two gene lists were then merged together resulting in 4,322 unique genes common between Affymetrix and Agilent datasets. Thereafter, the gene lists of both of the Affymetrix and Agilent log2-transformed data were each substituted with the 4,322 common gene list, followed by transforming each of them to z-scores^67^ in order to minimize the batch effect between the two platforms. The resultant two z-scored Affymetrix and Agilent lists were merged together, yielding a single dataset of 313 samples and 4,322 genes.

The steps of axolotl RNA-Seq data processing workflow can be found in Supplementary Fig. 2. Fastq files corresponding to samples from the 5 GSE datasets were obtained from the European Nucleotide Archive (ENA) database. Datasets with paired-end libraries are GSE116777, GSE92429, and GSE103087, whereas both GSE74372 and GSE34394 are single-end libraries. Recently, an axolotl transcriptome “V5 contig assembly” with contig length of 19,732 bp was published by Dwaraka *et al*.^56^, and was chosen to be our transcriptome reference. According to the authors, a total of 31,886 pairwise alignments with more than 98% sequence similarity were detected between V5 RNA-Seq contigs and the 20,036 V3 contigs which were used to design Affymetrix microarray probesets (AMBY_002a520748F) already having an annotation file. Therefore, since our microarray analysis pipeline included those microarray probesets along with its annotation file, a new annotation file for the 31,886 aligned contigs of the V5 assembly was generated for our RNA-Seq analysis pipeline by merging the 31,886 V5 contigs-V3 probesets alignment with V3 (AMBY_002a520748F) annotation file, resulting in around 25,000 genes. The transcriptome-wide quantifier “Salmon” tool^68^ was then used to separately quantify the expression of the transcripts of each of the 5 datasets by indexing the V5 transcriptome upon which direct quantification of the reads was carried out. The R/Bioconductor “tximport” package^69^ was used to import the scaled counts generated from transcript abundances along with our RNA-Seq annotation file, yielding a count matrix of all samples from the 5 datasests combined (32 samples) with 10,000 unique genes. Lowly expressed genes were filtered out by keeping genes having minimum counts for at least some samples using R/Bioconductor’s “edgeR” package^70,71^. The filtered count data was then converted to log2-counts per million (logCPM) and was made ready to be used for linear modeling for differential expression analysis using the “voom” function from R/Bioconductor’s “limma” package^72–74^. In order to deal with issues pertaining to batch effects, a design matrix consisting of group types (for contrasts) and GSE study origins accounting for each sample (for batch correction) was included in the “voom” function. Therefore, when the function is run, the resulting list of logCPM counts is that which has batch effects (study origins) accounted for.

### Differential expression analysis (DEA)

Microarray DEA was performed on the z-scored, combined data (313 samples, 4,322 genes), while RNA-seq DEA was performed on the logCPM (voom) counts (32 samples, 7,562 genes), respectively. The following analyses were performed using the R/Bioconductor “limma” package^72–74^. Wound healing vs. control, regenerative vs. control, and regenerative vs. wound healing comparisons were conceived for DEA of both microarray and RNA-Seq data. Design matrices were incorporated for each contrast to account for group type and study origin (batch factor) of every sample, followed by a fitted linear model on the expression data for each gene, which were then ranked based on an order of evident differential expression by applying the empirical Bayes method. False discovery rate (FDR) using the Benjamini-Hochberg (BH) method was controlled at 0.01, below which all genes were extracted representing lists of DE genes for each comparison from microarray and RNA-Seq data.

### Principal component analyses, clustered heatmaps, and correlations

In order to explore overall relationship occurring among samples of the z-scored microarray data and that of the “voom” counts RNA-Seq data, for each comparison, principal component analysis (PCA) was separately implemented on each of them in base R. PCA was also separately used on DE microarray and DE RNA-Seq data while minimizing the “study origin” batch effect using “limma” R package^72^ to illustrate how differentially expressed genes determine the clustering among samples, for every comparison. Complementary to both PCA approaches, heatmaps with Sample-to-Sample clustering based on “Manhattan” distance were implemented using “pheatmap” R package^75^. The distribution and correlation of the DE genes among the three comparisons within each technology and the subsequent comparison of correlation of the DE genes between the two technologies were carried out using Venn diagram online tool (http://bioinformatics.psb.ugent.be/webtools/Venn/) and scatter plots using the R/Bioconductor “ggplot2” package^76^. Pair-wise correlation testing of logFC of the DE genes for the three comparisons within and between the two technologies was performed using the “Spearman” correlation test in base R, as in^77–79^.

### Gene ontology enrichment analysis

Gene Ontology annotations of the top DE genes commonly identified by microarray and RNA-Seq DE analyses were carried out using Bioconductor’s “clusterProfiler” package in R^80^. The top up-regulated and down-regulated Entrez ID-mapped genes of each comparison were separately queried against the three GO (BP,CC,MF) categories. The background gene list was all Entrez IDs from axolotl Affymetrix annotation file. The organism database was set as homo sapiens “org.Hs.eg.db”, the adjusted p-value method was Benjamini & Hochberg (BH), and cutoffs for p and q values were set to 0.05 (to have a better comparability with previous studies which utilized “0.05” when identifying biological processes and pathways). Redundant GO terms were eliminated using “simplify” function from the clusterProfiler package^80^. The latter package was also used to visualize some GO categories and genes using a heatmap-like plot (heatplot) and a circular net (cnetplot). Bar plots of the top 10 terms of GO categories were generated using the R/Bioconductor “ggplot2” package^76^. Pathway analysis of the genes which were uniquely identified as DE by our IDA was performed using PathCards (Human biological pathway unification)^81^.

### Heatmap generation for top 100 regenerative vs. control genes

The top 100 DE genes detected by DEA of each technology in regenerative vs. control comparison were visualized in a clustered heatmap using “pheatmap” package in R^75^. The samples were clustered using the “Manhattan” distance measure. The genes were hierarchically clustered using “Complete Linkage” method. The values were centered and scaled in the row direction.

### Ethical statement, animal husbandry and qRT-PCR analysis

Animal care conditions and experimental procedures were approved by the local ethics committee of the Istanbul Medipol University (IMU) with authorization number 38828770-E.16123. All animal experiments were performed in accordance with relevant guidelines and regulations. Followed housing conditions, feeding regime and used anesthesia were described in [12,34]. Right forelimb of 9 axolotl was amputated at mid-zeugopod level. Amputated animals were selected randomly to form three groups (0, 1, and 7 days post amputation groups) and tissue samples were collected accordingly. To minimize the differences between individuals, 3 samples of each group pooled prior to RNA isolation. RNA was isolated from axolotl limb tissues (0,1 and 7 dpa) using TRIzol reagent (Invitrogen) by following the manufacturer’s instructions. RNA quantity was checked by spectrophotometrically using a NanoDrop ND-1000 (NanoDrop) and quality of isolated RNA was assessed by gel electrophoresis. M-MLV Reverse Transcriptase (Thermo Fisher Scientific) was used to perform reverse transcription according to manufacturer’s procedure. Quantitative PCR assays were performed at following conditions: initial denaturation at 95 °C for 2 minutes, and 40 cycles of denaturation at 95 °C for 5 seconds, annealing at 55 °C for 10 seconds and extension at 72 °C for 15 seconds. For qPCR reactions, SensiFAST™ SYBR® No-ROX Kit (BIOLINE, BIO-98005) and CFX Connect Real-Time PCR Detection System (BIO-RAD) was used. Gene expression levels were calculated using the 2−ΔΔCt method and cDNA concentrations were normalized with Ef1-α (elongation factor 1-alpha) housekeeping gene. Primers used in this study are listed in Supplementary Table 5.

## Results

### Whole gene expression data-based PCA and heatmap clustering

**Figure 2:**
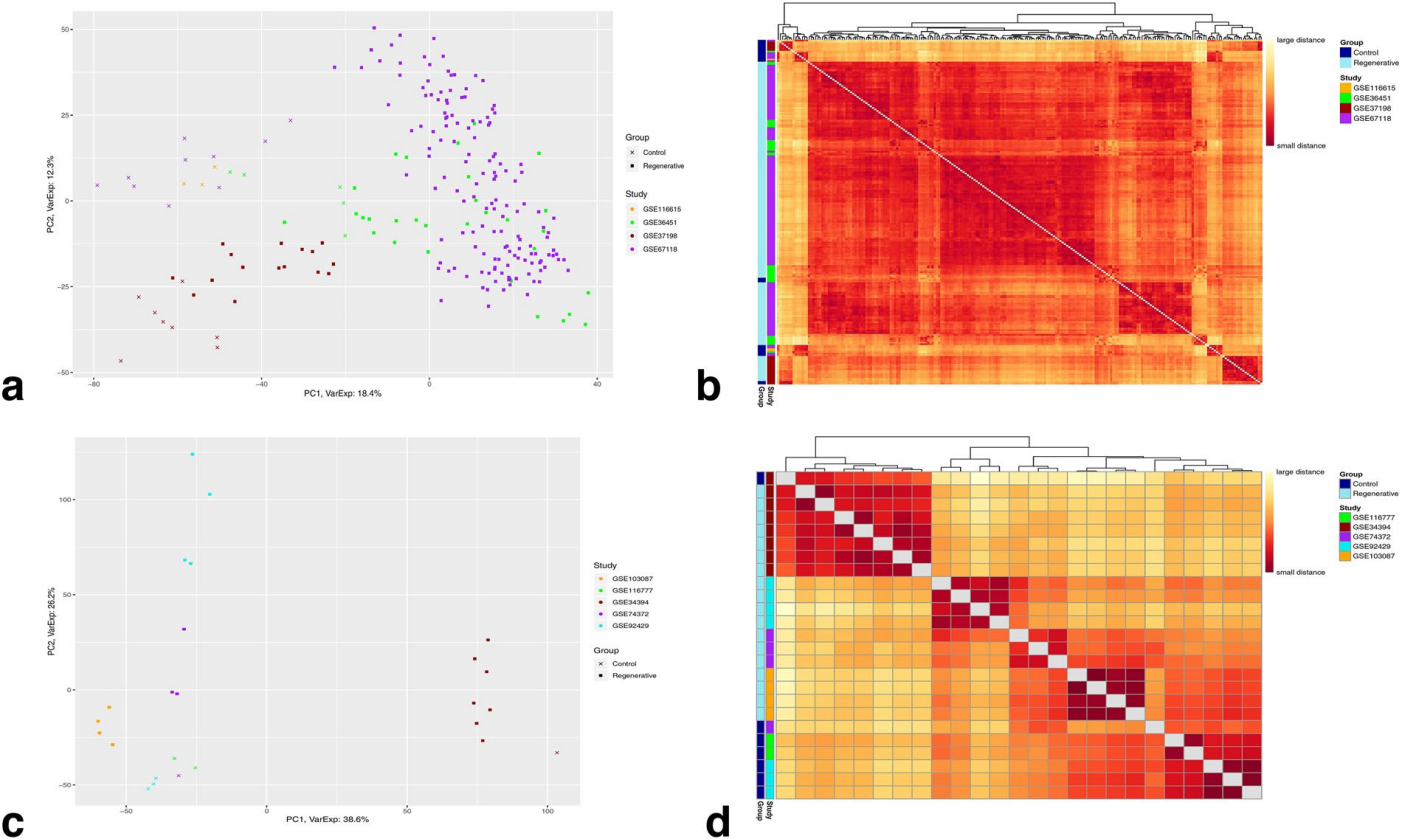
Clustering of the selected datasets. Whole gene expression data-based principal component analysis and sample-to-sample clustering heatmaps for control and regenerative samples of (**a,b**) Microarray quantile-normalized, log2-transformed, z-scored, data (4,322 genes, 220 samples) and of (**c,d**) RNA-seq logCPM (voom) counts (7,562 genes, 25 samples). (**a,c**) principle component analysis. (**b,d**) sample-to-sample clustering heatmap. The number of samples per group are; 26 control and 194 regenerative for microarray data; 7 control and 18 regenerative for RNA-Seq data.

The first principal component (PC1) as well as the sample-to-sample clustering heatmap of the z-scored, microarray data (4,322 genes) show a rough separation among the samples based on their group types; for control and regenerative samples (Fig. 2a,b) (PC1 = 18.4%, PC2 = 12.3%) and for the other two group pairs (Supplementary Figs. 3A,B and 4A,B) (PC1 = 18% and 21.8%, PC2 = 12.1% and 8.8%, respectively). However, PC2 seems to separate the samples based on their study origin. Therefore, the source of variation is likely explained by differential gene expression between the group types.

On the other hand, the separation among the samples displayed by PC1 in addition to the sample-to-sample clustering heatmap of the voom-counts, RNA-Seq data (7,562 genes) seems to be influenced by their study origin, conspicuously between GSE34394 and the rest; for control and regenerative samples (Fig. 2c,d) (PC1 = 38.6%, PC2 = 26.2%) and for the other two group pairs (Supplementary Figs. 3C,D and 4C,D) (PC1 = 58.2% and 43.6%, PC2 = 17.8% and 26.6%, respectively). PC2, however, seems to roughly separate the samples based on their group types.

In general, PCA and clustering of the gene expression data appears to emphasize an overall batch factor (study origin) which has a more dominant effect on how samples are separated than the time points (group types) in both microarray and RNA-Seq data.

### DEA-based PCA and heatmap clustering

DEA of the z-scored, microarray data resulted in 2,748 DE genes in regenerative vs. control, 2,092 DE genes in wound healing vs. control, and 3,166 DE genes in regenerative vs. wound healing, all having an adjusted p-value < 0.01. Furthermore, DEA of the voom-counts, RNA-Seq data yielded 2,992 DE genes in regenerative vs. control, while 423 genes and 2,371 genes were DE in wound healing vs. control and regenerative vs. control, respectively, all having an adjusted p-value < 0.01. The result of DEA for every comparison showed p-value enrichment near-zero peak corresponding to the number of DE genes from microarray data (Supplementary Fig. 5) and RNA-Seq data (Supplementary Fig. 6).

**Figure 3:**
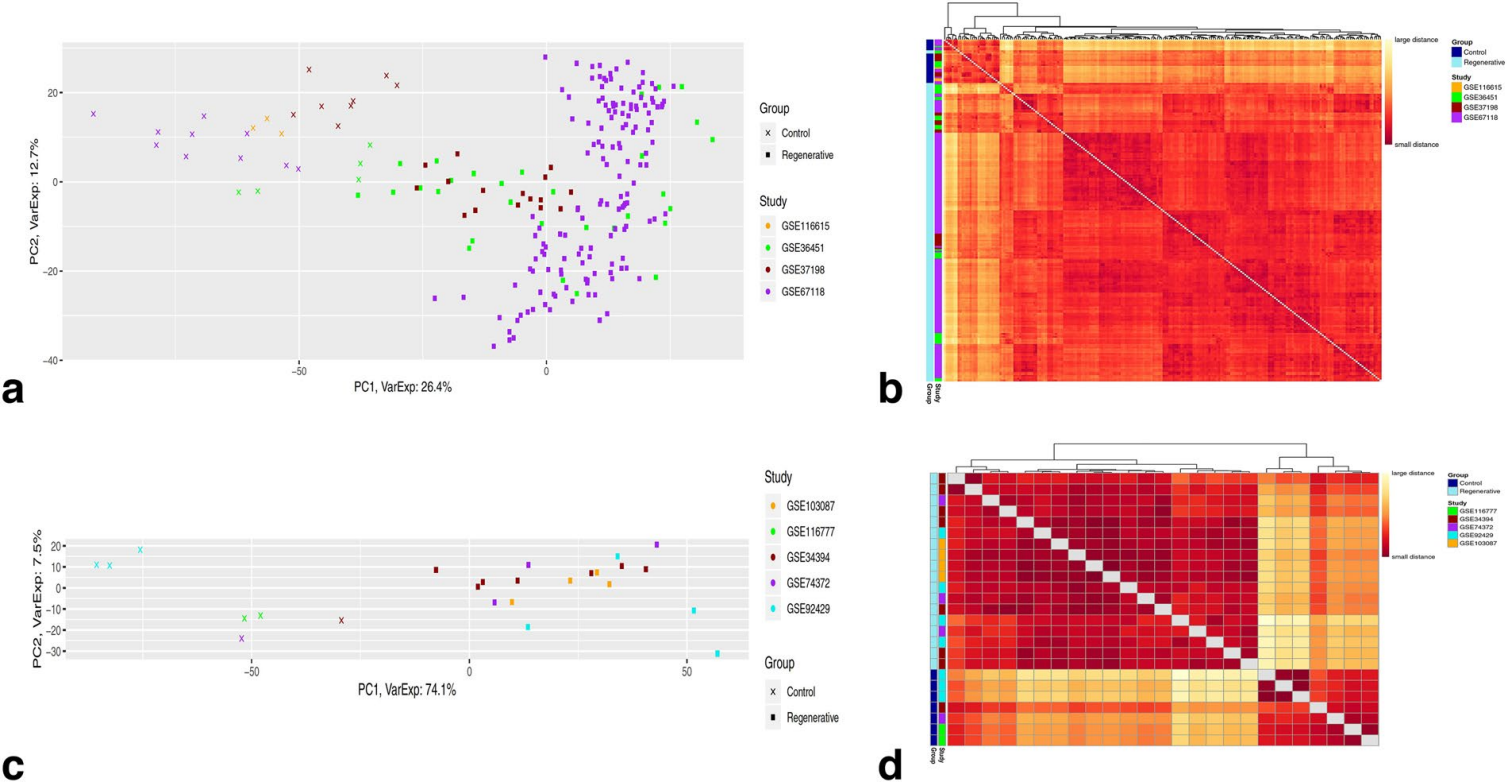
Clustering of the DE genes. DEA-based principal component analysis and sample-to-sample clustering heatmaps for regenerative vs. control comparison of (**a,b**) Microarray quantile-normalized, log2-transformed, z-scored data after DEA (2,748 DE genes, 220 samples) and of (**c,d**) RNA-seq logCPM (voom) counts data after DEA (2,992 DE genes, 25 samples). (**a,c**) principle component analysis. (**b,d**) sample-to-sample clustering heatmap. The number of samples per group are; 26 control and 194 regenerative for microarray data; 7 control and 18 regenerative for RNA-Seq data. The DE genes have an adjusted p-value < 0.01.

PC1 as well as the sample-to-sample clustering heatmap of the z-scored, microarray DEA-based data show a clear separation among the samples based on their group types; for regenerative vs. control (Fig. 3a,b) (PC1 = 26.4%, PC2 = 12.7%) and for the other two comparisons (Supplementary Figs. 7A,B and 8A,B) (PC1 = 30.8% and 31.7%, PC2 = 11.3% and 7.9%, respectively). However, PC2 indicates no strong separation of any type. Therefore, differential gene expression between the group types is evidently the dominant source of variation in this case. Likewise, the separation among the samples displayed by PC1 in addition to the sample-to-sample clustering heatmap of the voom-counts, RNA-Seq DEA-based data, unlike the whole gene expression data-based approach, is apparently based on their group types, while PC2 seems to point towards no particular separation of any type; for regenerative vs. control (Fig. 3c,d) (PC1 = 74.1%, PC2 = 7.5%) and for the other two comparisons (Supplementary Figs. 7C,D and 8C,D) (PC1 = 81.6% and 71%, PC2 = 6% and 6.2%, respectively). Thus, PC1 along with the heatmap clustering sufficiently and strongly demonstrate such high variation among the samples stems from differential expression between the group types.

Overall, the batch factor (study origin) has apparently been minimized during DEA as illustrated by the DEA-based PCA and clustering approach, which further demonstrated how group types are separated as a result of differential gene expression in both microarray and RNA-Seq data.

### Distribution of DE genes is evident among all three comparisons

**Figure 4:**
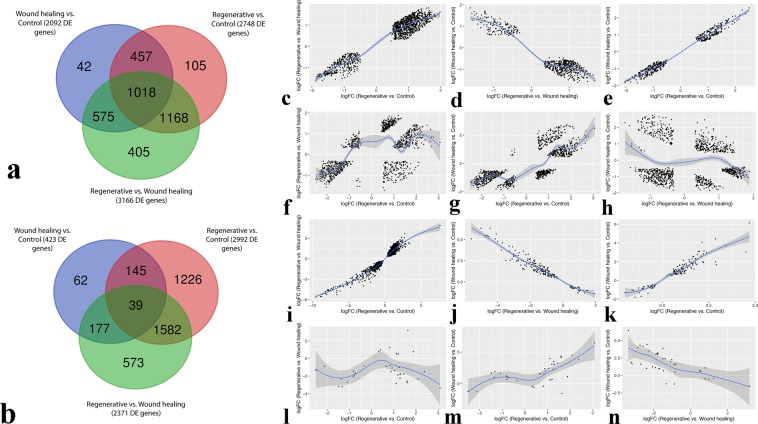
Comparison of DE genes. Venn Diagram of the distribution of significant genes (adjusted p-value < 0.01) among the three comparisons, from (**a**) Microarray data and (**b**) RNA-Seq data; (**a,b**) Each complete circle represents the number of differentially expressed genes of a certain comparison as resulted by DEA. (**c–h**) Scatter plots of the log-fold change of significant genes (adjusted p-value < 0.01) shared by two or more comparisons from microarray data (as shown in **a**). (**c**) Scatter plot of the logFC of the 1,168 DE genes shared by Regenerative vs. Control and Regenerative vs. Wound healing comparisons. (**d**) Scatter plot of the logFC of the 575 DE genes shared by Regenerative vs. Wound healing and Wound healing vs. Control comparisons. (**e**) Scatter plot of the logFC of the 457 DE genes shared by Regenerative vs. Control and Wound healing vs. Control comparisons. (**i–n**) Scatter plots of the log-fold change of significant genes (adjusted.p-value < 0.01) shared by two or more comparisons from RNA-Seq data (as shown in **b**). (**i**) Scatter plot of the logFC of the 1,582 DE genes shared by Regenerative vs. Control and Regenerative vs. Wound healing comparisons. (**j**) Scatter plot of the logFC of the 177 DE genes shared by Regenerative vs. Wound healing and Wound-healing vs. Control comparisons. (**k**) Scatter plot of the logFC of the 145 DE genes shared by Regenerative vs. Control and Wound healing vs. Control comparisons. (**f–h,l–n**) Pair-wise scatter plots of the logFC of the 1,018 and 39 DE genes shared by all three comparisons from microarray and RNA-Seq data, respectively; (**f,l**) Regenerative vs. Control and Regenerative vs. Wound healing, (**g,m**) Regenerative vs. Control and Wound healing vs. Control, (**H,N**) Regenerative vs. Wound healing and Wound healing vs. Control.

Although the distribution of DE genes is evident among comparisons (Fig. 4a,b), they may not necessarily share the same direction of gene regulation. In order to test this notion, correlations of the logFCs of the genes commonly DE in two or more comparisons were calculated in a pair-wise trend and visualized through scatter plots. Interestingly, the observed correlations within microarray data (Fig. 4c–h) share a similar pattern of gene regulation directionality with those in RNA-Seq data (Fig. 4i–n).

The Spearman correlation coefficient (r_s_) for logFCs of the genes commonly DE in regenerative vs. control and regenerative vs. wound healing in microarray and RNA-Seq data are 0.85 and 0.94, respectively (Fig. 4c,i). Likewise, the coefficients for logFCs of the genes commonly DE in regenerative vs. control and wound healing vs. control in microarray and RNA-Seq data are 0.96 and 0.93, respectively (Fig. 4e,k). On the other hand, the coefficients for logFCs of the 575 genes and 177 genes commonly DE in wound healing vs. control and regenerative vs. wound healing in microarray and RNA-Seq data are - 0.71 and - 0.95, respectively (Fig. 4d,j). The pair-wise correlations for the 1,018 genes and 39 genes DE in all three comparisons in microarray (Fig. 4f–h) and RNA-Seq (Fig. 4l–n) data, respectively, demonstrate that those genes follow the same trend as when they are shared by only the two corresponding comparisons. From microarray data, the 1,018 genes DE in regenerative vs. control and regenerative vs. wound healing are positively correlated (r_s_ = 0.52), in regenerative vs. control and wound healing vs. control are positively correlated (r_s_ = 0.77), and in regenerative vs. wound healing and wound healing vs. control are negatively correlated (r_s_ = −0.02). From RNA-Seq data, the 39 DE genes have an r_s_ = 0.03, r_s_ = 0.54, and r_s_ = −0.71 for the latter three comparisons, respectively.

### Some DE genes are commonly detected by the analyses of microarray and RNA-Seq data

**Figure 5:**
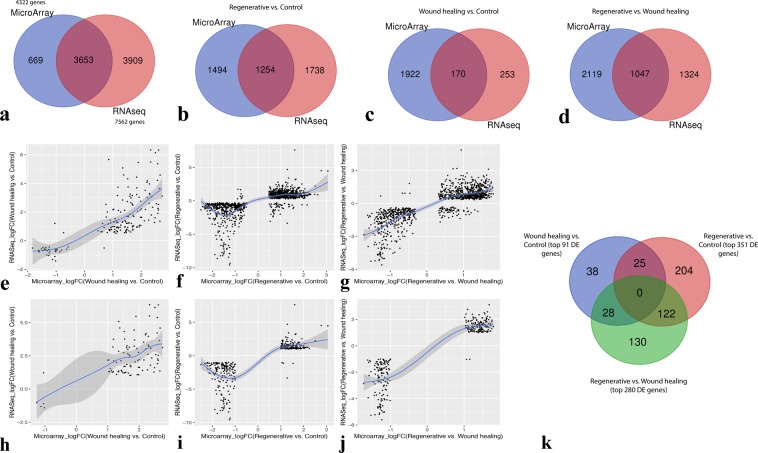
Identification of the common DE genes. (**a**) Venn Diagram of the initial number of genes used before differential expression analysis. This number for each platform is obtained just after the filtering and/or merging. (**b–d**) Venn diagrams of the distribution of DE genes (adjusted p-value < 0.01) between Microarray and RNA-seq data as well as (**e–g**) scatter plots of the log-fold change of the common DE genes between the two technologies for (**b,e**) wound healing vs. control, (**c,f**) regenerative vs. control, and (**d,g**) regenerative vs. wound healing. (**h–j**) Scatter plots of the log-fold change of the top DE genes (adjusted p-value < 0.01, logFC magnitudes >1) commonly identified by Microarray and RNA-seq technologies’ analyses for (**h**) wound healing vs. control (91 top DE genes), (**i**) regenerative vs. control (351 top DE genes), and (**j**) regenerative vs. wound healing (280 top DE genes). (**k**) Venn Diagram of the distribution of the top DE genes (adjusted p-value < 0.01, logFC magnitudes >1) commonly identified by the analyses of both technologies among the three comparisons.

Microarray and RNA-Seq DEA was carried out on the 4,322 and 7,562 genes, respectively. Since the same annotation source (AMBY_002a520748F) was used for the analyses from both technologies, a total of 3,653 genes are common between them (Fig. 5a). Therefore, differing numbers of DE genes per comparison that would be commonly identified by the analyses of both technologies is conceivable. Indeed, after merging the DE gene list (adjusted p-value < 0.01) of microarray data with that of RNA-Seq data for each comparison, we found 170, 1,254, and 1,047 DE genes commonly identified by both technologies in wound healing vs. control, regenerative vs. control, and regenerative vs. wound healing, respectively (Fig. 5b–d). Each set of those common DE genes per comparison were tested for their logFC correlation between both technologies and were visualized using scatter plots. The common DE genes of all three comparisons had positive correlations between the two technologies; r_s_ = 0.74 for wound healing vs. control, r_s_ = 0.71 for regenerative vs. control, and r_s_ = 0.77 for regenerative vs. wound healing (Fig. 5e–g).

In order to decrease the number of genes with low logFCs as well as those with opposite expression directionality between data of both technologies, the top DE genes can be extracted by a criterion. Firstly, genes with logFC magnitudes >1 are separately extracted from both microarray and RNA-Seq DE lists. Next, the resultant two lists are merged to yield the top DE genes commonly detected by DEA of both technologies. The extracted top DE genes are 91 genes in wound healing vs. control, 351 genes in regenerative vs. control, and 280 genes in regenerative vs. wound healing (Supplementary Table 6**)**. The correlation of the logFCs of those top DE genes between the two technologies is positive for wound healing vs. control (r_s_ = 0.44), regenerative vs. control (r_s_ = 0.72), and regenerative vs. wound healing (r_s_ = 0.76) (Fig. 5h–j). Those top DE genes were merged together to see whether they are distributed among the three comparisons. Indeed, some of them are commonly DE in more than one comparison. However, notably, the number of genes commonly DE in all three comparisons is zero, and the number of DE genes specific to each comparison is relatively high (Fig. 5k).

### GO enrichment of the top DE genes

**Figure 6:**
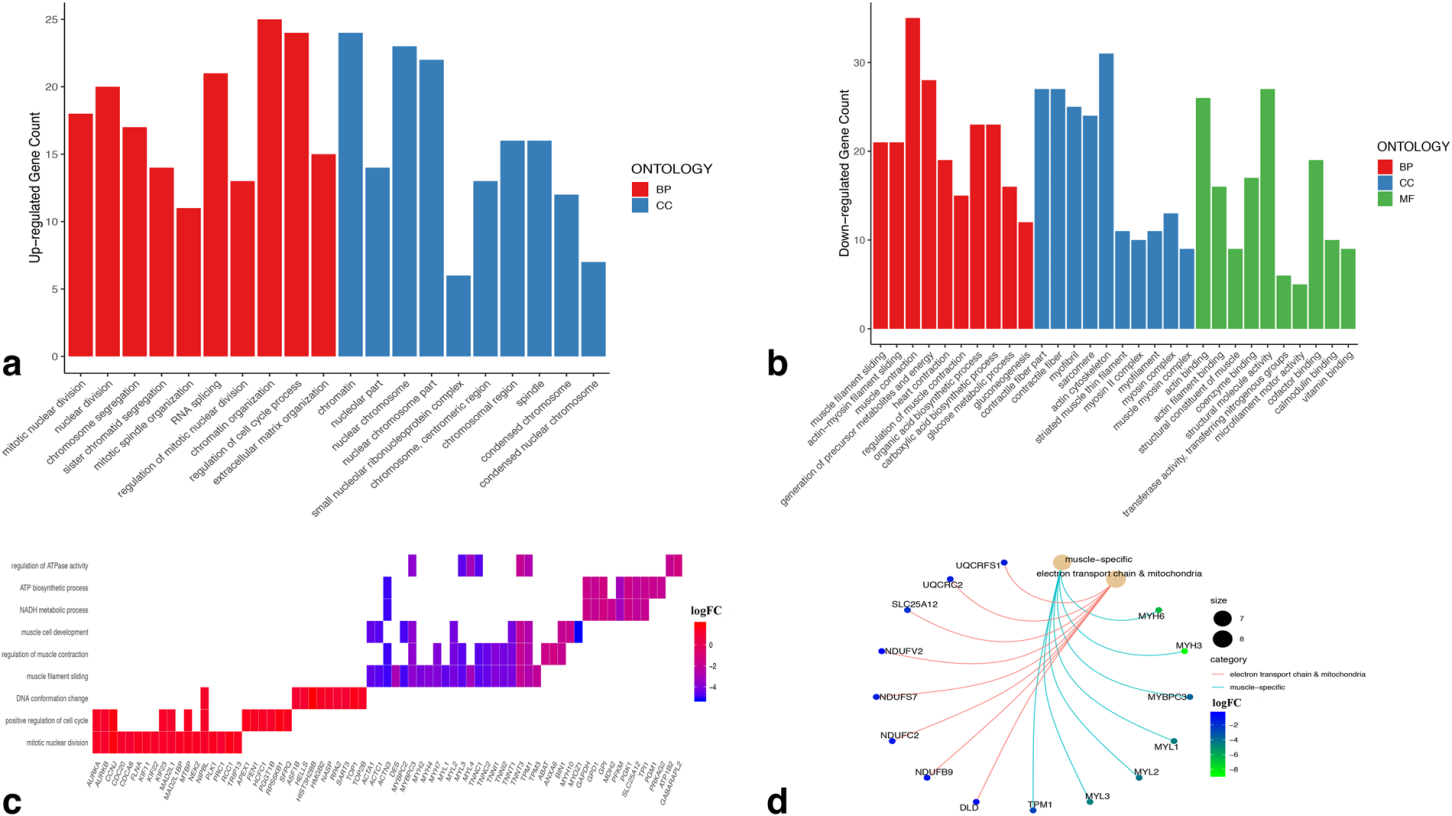
Gene ontology analysis of the DE genes. (**a**) top 10 GO terms of each of biological processes and cellular components enriched by the top 181 up-regulated genes in regenerative vs. control comparison commonly identified by both technologies’ analyses. (**b**) top 10 GO terms of each of biological processes, cellular components, and molecular functions enriched by the top 166 down-regulated genes in regenerative vs. control comparison commonly identified by both technologies’ analyses. (**c**) Biological processes related to cell cycle, muscle-tissues, and metabolism enriched by some of the top up-regulated and down-regulated genes in regenerative vs. control comparison. (**d**) some of the top DE muscle-specific genes and other DE metabolic genes being down-regulated in regenerative vs. control comparison at different rates.

Our top DE genes commonly detected by the DEA of each of the two technologies enriched a variety of GO terms for each comparison (Supplementary Tables 7–9). Biological processes such as mitotic nuclear division, regulation of cell cycle process, and ECM organization were found among the top 10 BPs enriched by the top 181 up-regulated regenerative vs. control genes (Fig. 6a). Those up-regulated genes also enriched several cellular components, such as chromatin and spindle. On the other hand, muscle filament sliding, muscle contraction, and generation of precursor metabolites and energy were detected among the top 10 biological process enriched by the top 166 down-regulated regenerative vs. control genes (Fig. 6b). Cellular components such as contractile fiber and actin cytoskeleton were enriched by those down-regulated genes. The latter genes also enriched several molecular functions, including actin filament binding and calmodulin binding.

### Heatmap of top 100 DE genes

**Figure 7:**
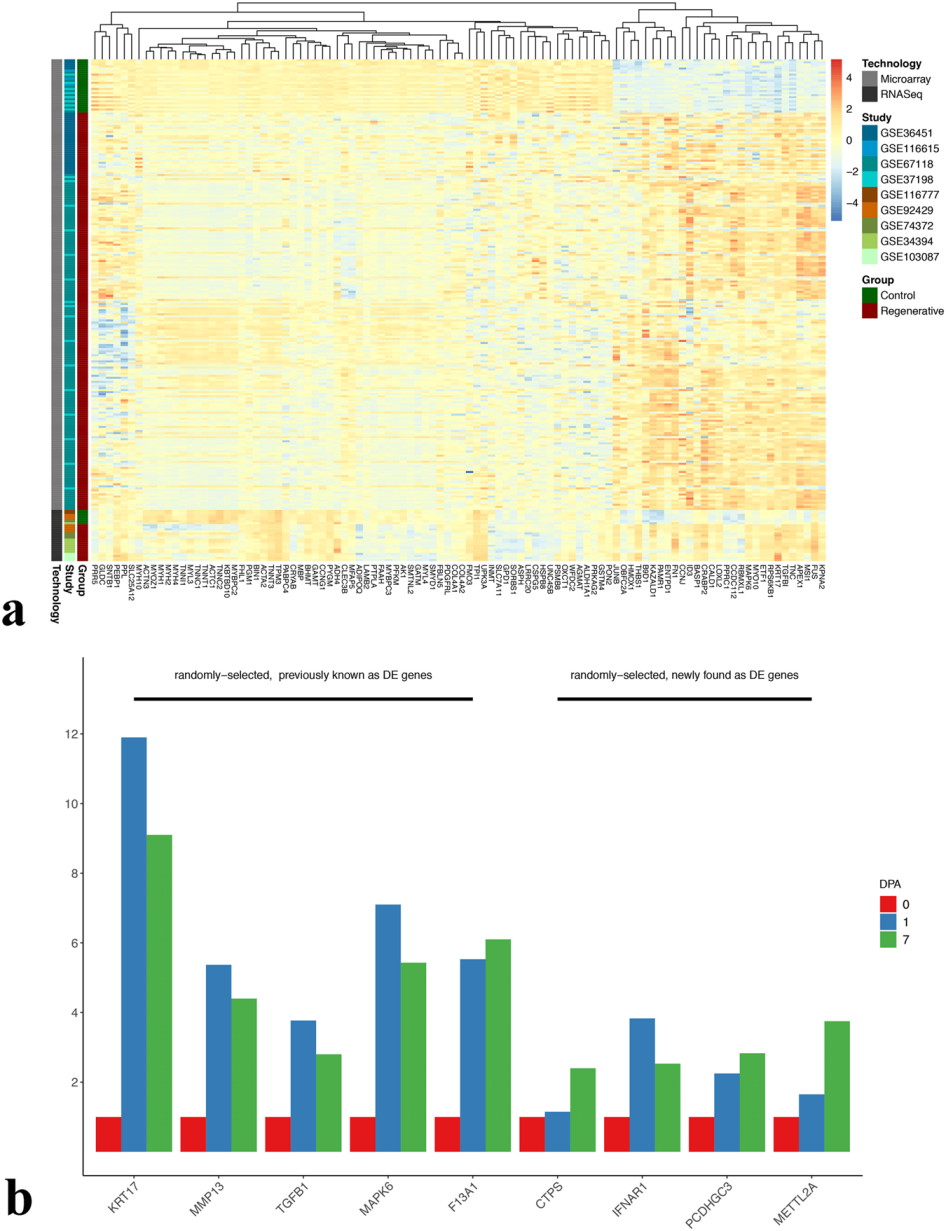
Visualization and qRT-PCR of DE genes. (**a**) clustered heatmap of the top 100 DE genes commonly detected by the analyses of both technologies in regenerative vs. control comparison. (**b**) qRT-PCR of 5 randomly-selected genes previously known to be DE, and 4 other randomly-selected genes uniquely detected as DE by our IDA. All genes are up-regulated at 1 and 7 dpa compared to day0. KRT17: Keratin 17, MMP13: Matrix Metallopeptidase13, TGFB1: transforming growth factor beta 1, MAPK6: Mitogen-Activated Protein Kinase 6, F13A1: Coagulation Factor XIII A Chain, CTPS: CTP Synthase, IFNAR1: Interferon Alpha And Beta Receptor Subunit 1, PCDHGC3: Protocadherin Gamma Subfamily C 3, METTL2A: Methyltransferase Like 2A.

In order to look at the most DE genes detected by the DEA of both microarray and RNA-Seq data in regenerative samples compared to the controls all in a single and interpretable plot, the top 100 DE genes were selected and visualized in a gene-wise hierarchically-clustered heatmap (Fig. 7a). The clustering shows an overall conspicuous clustering between regenerative and control samples from data of each technology.

### Validation of candidate DE genes by qRT-PCR

To test the accuracy of our analysis, qRT-PCR was conducted for several randomly-selected genes from the 351 regenerative vs. control genes commonly identified as DE by both microarray and RNA-Seq analyses. Two groups of genes were selected. The first one consists of 5 randomly-selected genes previously well known as DE in axolotl limb regeneration (*MAPK6, Keratin17, TGFB1, MMP13*), and *Coagulation factorXIII* which is more evidently expressed in *Cynops orientalis* limb and *Pleurodelinae* (newt) lens regeneration^57,82–86^. The second group consists of four randomly-selected genes from the ones uniquely found as DE by our methodology (*CTPS*, *IFNAR1*, *PCDHGC3*, *METTL2A*). The expression levels of all these genes were upregulated at day1 and day7 compared to day0 post-amputation (Fig. 7b), which is consistent with the output of our IDA.

## Discussion

To our knowledge, this is the first study describing an integrative analysis methodology by which publicly available microarray and RNA-Seq axolotl data were leveraged in order to identify DE genes marking the wound healing and regenerative phases of the axolotl limb.

Despite the more frequent and persistent application of “meta-analysis” in the literature in contrast to “merging”^43^, the latter has been applied in several studies^43,87–89^ and was chosen for the purpose of our study. It has been postulated that computing separate statistics and taking the average is often less powerful compared to aggregation of data as a first step and then deriving statistics from this data^42,43,45,90^.

While the merging method recognizes all samples coming from different datasets across different platforms as a single dataset when testing the same hypothesis, the existence of systematic biases may introduce unwanted batch effects (non-biological differences) during the analysis of gene signatures and, consequently, true biological differences can be masked among the conditions of interest^42,43,51,91^. Moreover, several intra-laboratory variables that may have ambiguous or absent GEO entries such as amputation site, size, feeding, and maintenance protocols of axolotls are amongst many factors which can influence how control and test samples cluster together, and probably contributing to the observed batch effect in whole gene expression data-based PCA and clustering heatmap (Fig. 2 and Supplementary Figs. 3 and 4). Nonetheless, preservation of true biological (gene-expression) differences between experimental groups were successfully attained after DEA and visualized through PCA and clustering heatmap (Fig. 3 and Supplementary Figs. 7 and 8), indicating an indecisive role of any intra-laboratory variables. Notably, minimization of batch effects and maximization of true gene-expression differences were mainly due to the application of transformation and normalization techniques during data processing^43,67^ along with accounting for the experiment source (study origin) for each sample while performing DEA^72^.

The consistency of the results obtained from each of microarray and RNA-Seq DEAs with one another indicates a fairly valid approach of integrative analysis that we took. First and foremost, the correlations of each pair-wise distribution of the DE genes among all three comparisons from microarray data (Fig. 4a,c–h) accord with those from RNA-Seq data (Fig. 4b,i–n). Positive correlations are always observed among the genes commonly DE in regenerative vs. control and regenerative vs. wound healing (Fig. 4c,f,i,l), as well as among those commonly DE in regenerative vs. control and wound healing vs. control (Fig. 4e,g,k,m). Further, negative correlations are always observed among the genes commonly DE in regenerative vs. wound healing and wound healing vs. control (Fig. 4d,h,j,n). This also suggests a possibly true underlying biological behavior of those genes in their respective comparisons (Supplementary Table 10). Secondly, DE genes commonly detected by DEA of each of the two technologies are always positively correlated between microarray and RNA-Seq data, along with their top DE genes, for every comparison (Fig. 5b–j). Several previous studies have also reported such strong positive correlations between microarray and RNA-Seq data^78,79,92,93^. Notably, a small number of genes have opposite gene expression direction, which is probably attributable to artifacts of gene expression technologies. Nevertheless, the number of those genes with an opposite direction was found significantly low for the top DE genes (Fig. 5h–j).

Some of the top DE genes detected by both technologies in wound healing vs. control comparison concur with previously identified genes in the wound healing response. In wound healing process, at around 6–8 hours post-amputation (hpa), epithelial cells tend to migrate to the amputation site to form the “wound epithelium (WE)” beneath which are cellular and extracellular debris as well as a damaged vasculature^57,94^. Basel cells of this wound epidermis lacking a basement membrane at 24 hpa are characterized with highly up-regulated thbs1 gene^56,95^, which was found as a top up-regulated gene in our wound healing vs. control list. Furthermore, matrix metalloproteinases activity is required to regulate the extracellular matrix and these proteins are enriched in the wound epidermis as soon as an injury takes place^96^. Our wound healing vs. control top DE genes list also included metalloproteinases such as *mmp1*, *mmp3*, *mmp19*, and their regulator *timp1*, in addition to the extracellular matrix-generating component *tnc*^56^. Complete list of the top DE genes in wound healing vs. control is presented in Supplementary Table 6 and GO terms enriched by them are documented in Supplementary Table 7.

Genes which were previously implicated in the regenerative process also concord with some of our top DE genes detected by both technologies in regenerative vs. control comparison. Following 2 dpa, processes such as DNA replication, mitosis, and cell cycle are enriched by a set of up-regulated genes the majority of which signify a transition phase in the limb regeneration program during 2–3 dpa interval^18^. After 3 dpa, those genes either undergo an increased rate of up-regulation or sustain a relatively constant expression until 28 dpa^18^. Concordantly, many gene ontology terms, particularly those related to cell cycle, were enriched by many of our top up-regulated regenerative vs. control genes, such as mitotic nuclear division, positive regulation of cell cycle, and DNA conformation change (Fig. 6c). This punctuated increase of cell cycle transcripts is, therefore, indicative of a striking change in the population of proliferative cells taking place at the stump of the distal limb^18^. Besides, earlier studies have reported significant reduction of muscle-specific genes over the course of limb regeneration^17,18,56,60,97^. During early response to limb amputation, the limb stump also undergoes muscle tissue remodeling along with diminished levels of muscle-specific transcripts^56^. The reduction in the expression of muscle-specific transcripts, however, becomes so significant by around 10 dpa which strongly implies complete absence of muscle tissues^18^. Therefore, depletion of muscle tissues in such an absolute manner is propounded to be an essential step towards the recruitment of progenitor cells and the initiation of blastemal outgrowth^56^. This observation is also in line with our top down-regulated regenerative vs. control genes which enriched many muscle-specific GO terms, including muscle filament sliding, regulation of muscle contraction, and muscle cell development (Fig. 6c). In addition to the reduction of muscle-specific transcripts by 10 dpa, expression levels of some transcripts associated with metabolic processes also markedly drop by around the same time^18,56^. Indeed, some of our top down-regulated regenerative vs. control genes enriched several GO terms associated with cellular metabolic processes, such as NADH metabolic process, ATP biosynthetic process, and regulation of ATPase activity (Fig. 6c).

Another interesting observation is that cellular metabolic genes are still expressed by the cells which do not die or undergo reprogramming. Therefore, non-absolute, moderate depletion of some metabolic genes in contrast to the complete reduction of muscle-specific genes during the regenerative phase was previously shown^56^. This relationship was also discovered in our regenerative vs. control comparison between top down-regulated muscle-specific genes and some of the metabolic genes functioning in electron transport chain and mitochondria, most of which are not among the top DE genes (Fig. 6d). As depicted, those metabolic genes are much less down-regulated than the muscle-specific genes during the regenerative phase.

When discussing “top DE genes” or “DE genes”, we refer to those genes commonly detected by the DEA of each of microarray and RNA-Seq. However, an important point to underline here is that two of the muscle-specific genes (*myh6*, *myh3*) and a metabolic gene (*ndufv2*) from Fig. 6d were detected DE (adjusted p-value < 0.01) only by RNA-Seq DEA. Since Affymetrix and Agilent data’s gene lists were merged together, the resultant microarray genes did not completely match those of RNA-Seq which were annotated using the Affymetrix annotation file. Consequently, many genes which were used for RNA-Seq DEA were not represented in the final 4,322 microarray gene list. This is, therefore, indicative of the fact that the reliability of our results may be extended to those DE genes specifically detected by only RNA-Seq DEA, and not to be limited to those commonly detected by the DEA of each of the two technologies. Excluding the Agilent dataset from microarray DEA could -probably- lead to less complicated downstream analysis. On the other hand, sample size is often a key for and a fundamental premise of having a successful and powerful integrative analysis; and in this study, this concept was further fulfilled by integrating the Agilent dataset.

In addition to the previously identified DE genes, we aimed to find a set of genes uniquely found as DE by our IDA in our top upregulated regenerative vs. control gene list. We checked for genes that have been overlooked, absent, inconsistently found as DE, or labeled insignificant by the studies used in the IDA. Interestingly, we found at least 23 upregulated genes which may be now considered as novel candidate genes enriched in axolotl limb regeneration (Supplementary Table 11). The differential expression of four of these genes were consistently validated by qRT-PCR (Fig. 7b). This finding gives credence to the usage of IDA as a powerful statistical approach to both enhance the confidence that a gene is DE and detect new DE genes that may have been overlooked by previous studies due to factors such as limited sample size and diminished statistical power.

There are some inherent limitations about this study that need to be addressed. Despite our successful minimization of batch effects across experiments, the merging methodology cannot guarantee their complete removal. In some cases, the quality of the deposited, original data could play a deterministic role in the acquisition of the most statistically-sound downstream results. The major limitation to underline, however, lies within the concept of data integration itself along with the gene-level data processing approach we took. Integrative analysis of different platforms provides statistical power through increased sample size at the expense of having a large number of genes for differential expression and downstream analyses. The process of merging the genes of Affymetrix platform with those of Agilent’s showed a decrease in the total number of microarray genes which were used for DEA. Moreover, the approach of annotating our integrated RNA-Seq data eliminated a plethora of transcripts which could be important in the limb regeneration process. Therefore, a “meta-analysis” approach which would utilize probeset-level information could solve the aforementioned issues. Nevertheless, since we rather chose the “merging” approach on the premise that it is well suited for both our experimental design and biomarker discovery, it was an absolute necessity to collapse information from probe/contig-level to gene-level. It is also plausible to conceive the RNA-Seq annotation process from the list of Affymetrix-Agilent genes instead of the whole Affymetrix annotation file, which would also lead to a decrease in the total number RNA-Seq genes to be used for DEA, not to mention the further decrease due to filtering steps. Therefore, in this study, we tried to sustain a trade-off between increased statistical power through integration of data and having the maximum possible number of genes to be analyzed.

## Conclusion

To the best of our current knowledge, this is the first study describing an integrative analysis of publicly available microarray and RNA-Seq axolotl data, with an aim to uncover DE genes signifying the wound healing and regenerative phases of the axolotl limb. The validity of our DEA methodology can be realized from observing the same pattern of gene expression directionality within data of each technology, and the positive correlation of the DE genes between both technologies. Our results included many DE genes enriched in a wide variety of biological processes in accord with those described in previous studies. Some genes were uniquely detected as DE by our IDA approach. qRT-PCR experiments (Fig. 7b) provide another layer of evidence to validate our computational findings. Future direction of this study would aim to explore the putative functions of the newly found DE genes during regeneration. Our methodology can be repeated with more axolotl data as we anticipate more will become available in near future.

## Supplementary information

Supplementary Dataset.
