# Supplementary material for: Integrative Analysis of Axolotl Gene Expression Data from Regenerative and Wound Healing Limb Tissues
Source: Sci Rep. 2019 Dec 30;9:20280. doi: 10.1038/s41598-019-56829-6 (PMC6937273; doi:10.1038/s41598-019-56829-6)
Supplement: Supplementary file 1 — Supplementary Dataset. [file 41598_2019_56829_MOESM1_ESM.pdf]

Integrative Analysis of Axolotl Gene Expression Data from Regenerative and Wound Healing Limb Tissues

Mustafa Sibai [1,*], Cüneyd Parlayan [2,3,*,#], Pelin Tuğlu [2], Gürkan Öztürk [2,4] , Turan Demircan [2,5#]

1) Graduate School of Engineering and Natural Sciences, Istanbul Medipol University, Istanbul,

Turkey.

2) Regenerative and Restorative Medicine Research Center, REMER, Istanbul Medipol University, Istanbul, Turkey.

3) Department of Biomedical Engineering, Faculty of Engineering, İstanbul Medipol University,

Istanbul, Turkey.

4) Department of Physiology, International School of Medicine, İstanbul Medipol University, Istanbul, Turkey.

5) Department of Medical Biology, School of Medicine, Mugla Sitki Kocman University, Mugla,

Turkey.

*These authors contributed equally

#To whom correspondence may be addressed:

Turan Demircan, PhD,

Mugla Sitki Kocman University, School of Medicine, Mentese, Mugla, Turkey.

Tel: +90-553-8068948 Fax: +90-252-2111345.

E-mail: turandemircan@mu.edu.tr

Cuneyd Parlayan, PhD,

Istanbul Medipol University, Faculty of Engineering, Istanbul, Turkey

Tel: +90-216-6815100, Fax: +90- 212-5212377.

E-mail: cparlayan@medipol.edu.tr

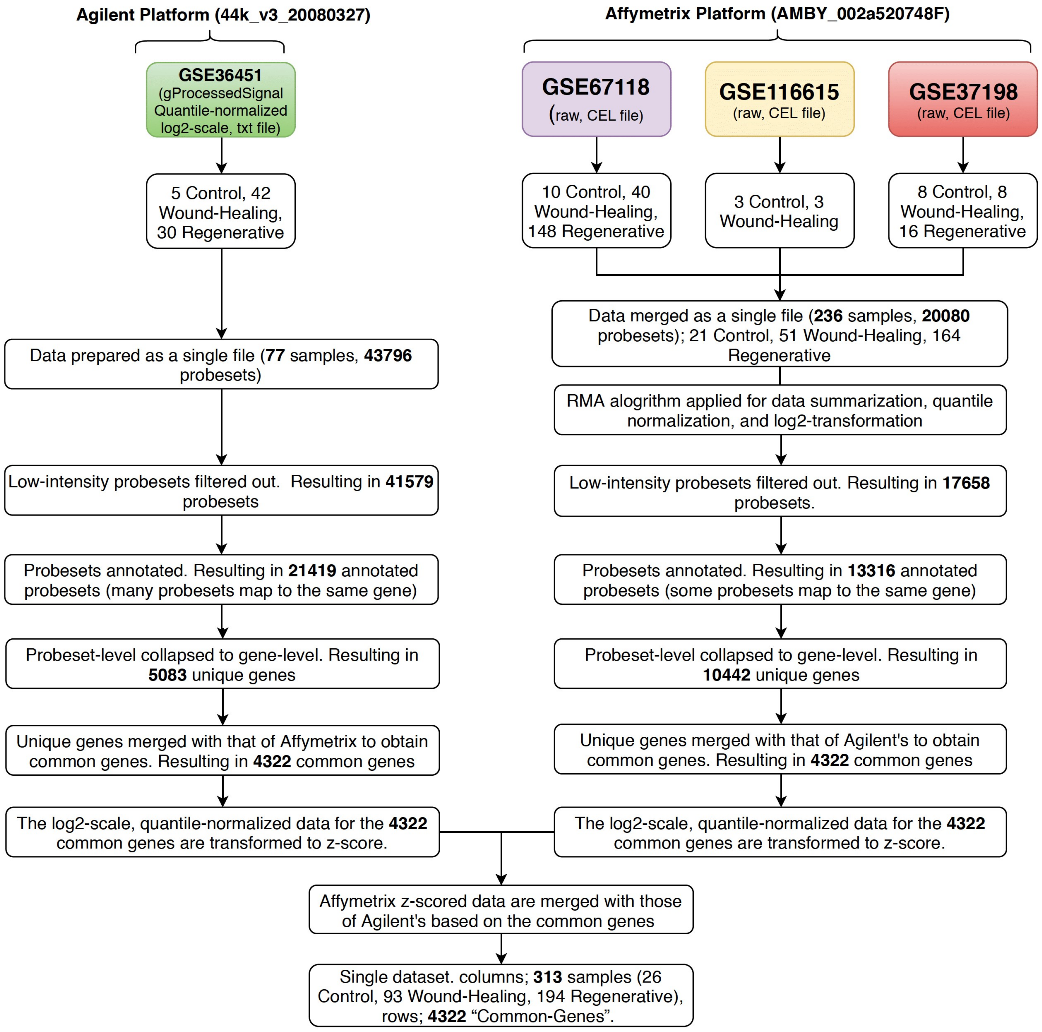

Supplementary Figure 1: Detailed workflow for Affymetrix and Agilent Microarray axolotl data processing prior to differential expression analysis.

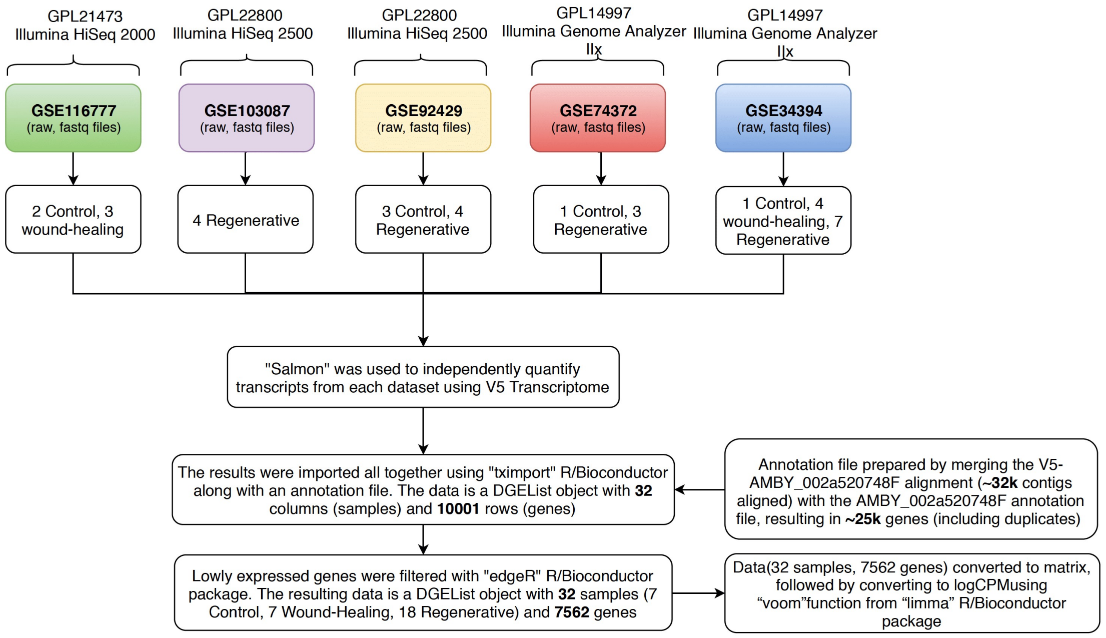

Supplementary Figure 2: Detailed workflow for Illumina RNA-Seq data processing prior to differential expression analysis.

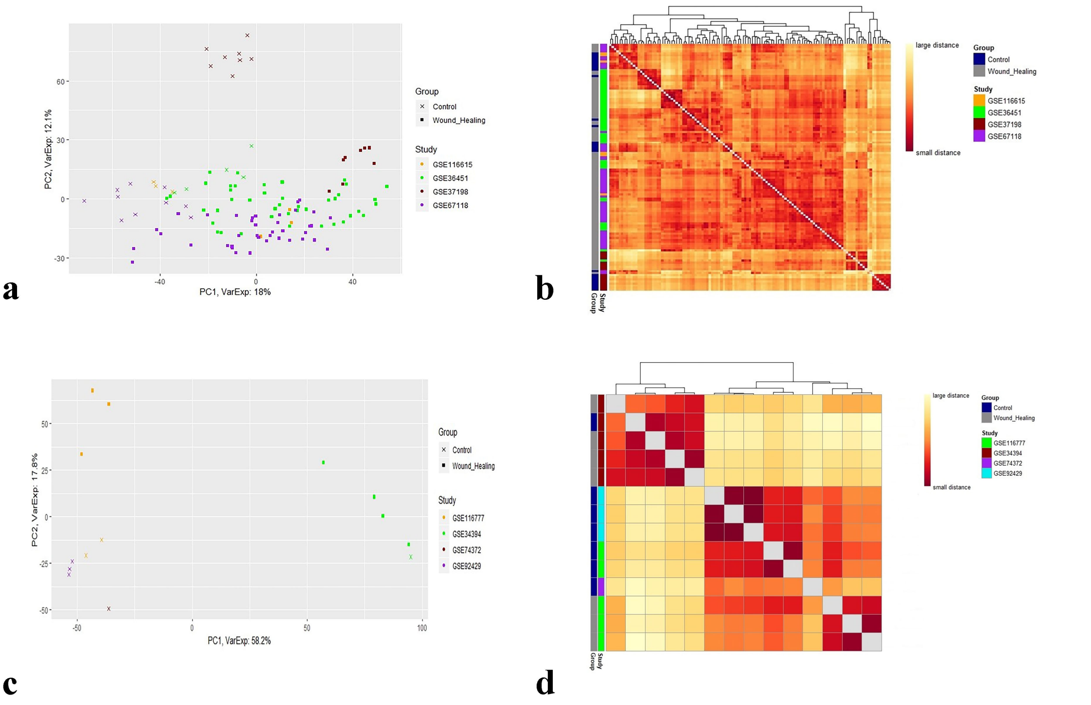

Supplementary Figure 3: Whole gene expression data-based principal component analysis and sample-to-sample clustering heatmaps for control and wound healing samples of A,B) Microarray quantile-normalized, log2-transformed, z-scored data (4,322 genes, 119 samples) and of C,D) RNA-seq logCPM (voom) counts data (7,562 genes, 14 samples)**.** A,C) principle component analysis. B,D) sample-to-sample clustering heatmap. The number of samples per group are; 26 Control and 93 wound healing for microarray data; 7 control and 7 wound healing for RNA-Seq data.

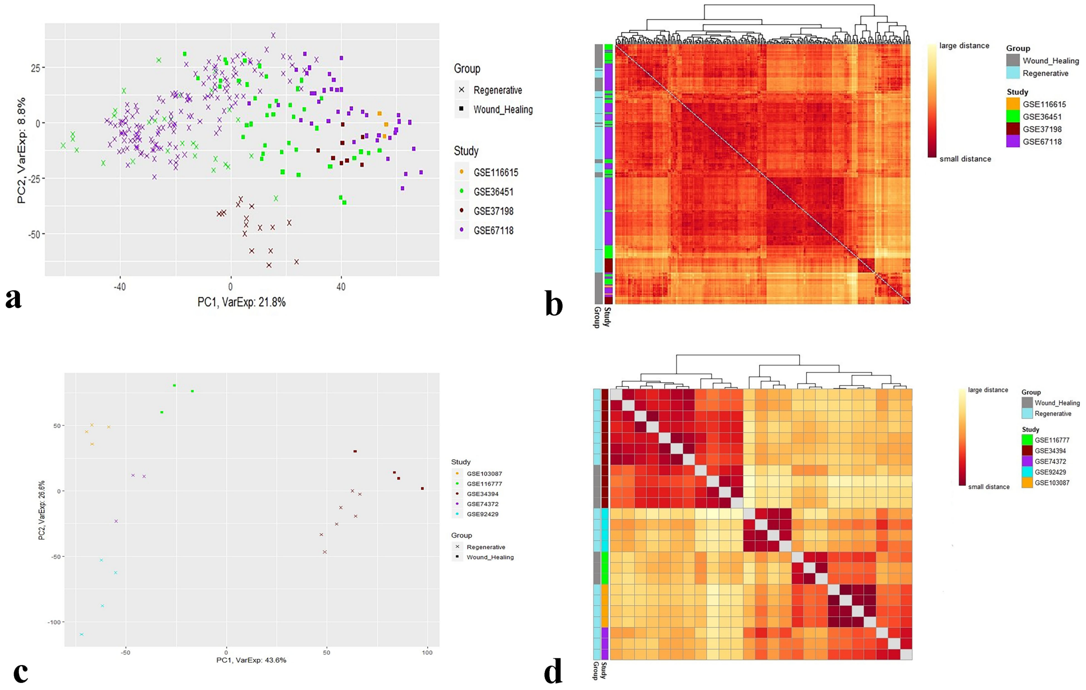

Supplementary Figure 4: Whole gene expression data-based principal component analysis and sample-to-sample clustering heatmaps for regenerative and wound healing samples of A,B) Microarray quantile-normalized, log2-transformed, z-scored data (4,322 genes, 287 samples) and of C,D) RNA-seq logCPM (voom) counts data (7,562 genes, 25)**.** A,C) principle component analysis. B,D) sample-to-sample clustering heatmap. The number of samples per group are; 194 regenerative and 93 wound healing for microarray data; 18 regenerative and 7 wound healing for RNA-Seq data.

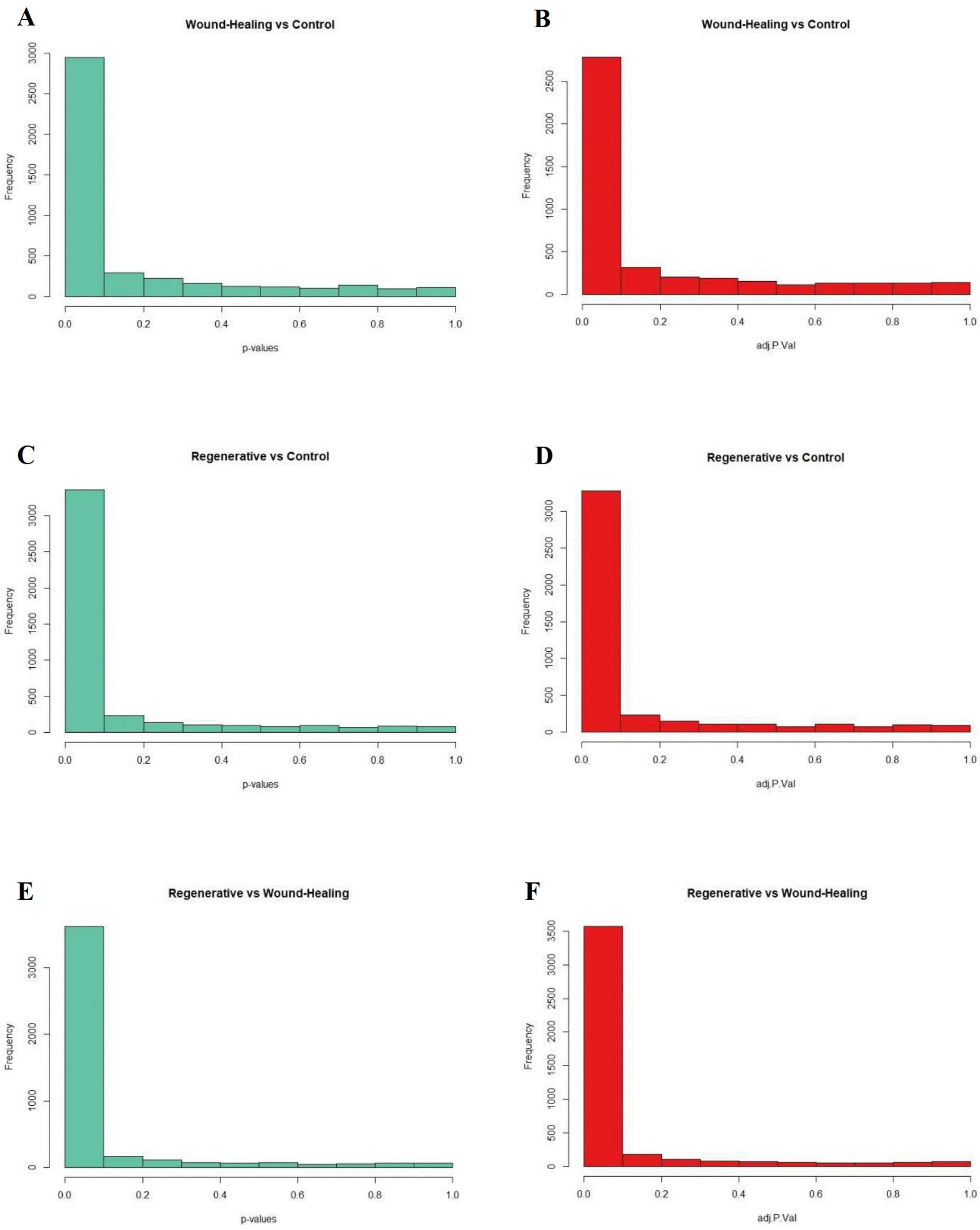

Supplementary Figure 5: Raw and adjusted p-values distribution of microarray DEA results. p-value histogram (left) and adjusted p-value histogram (right) were plotted after DEA on Microarray data for the following comparisons A,B) wound healing vs. control, C,D) regenerative vs. control, E,F) regenerative vs. wound healing.

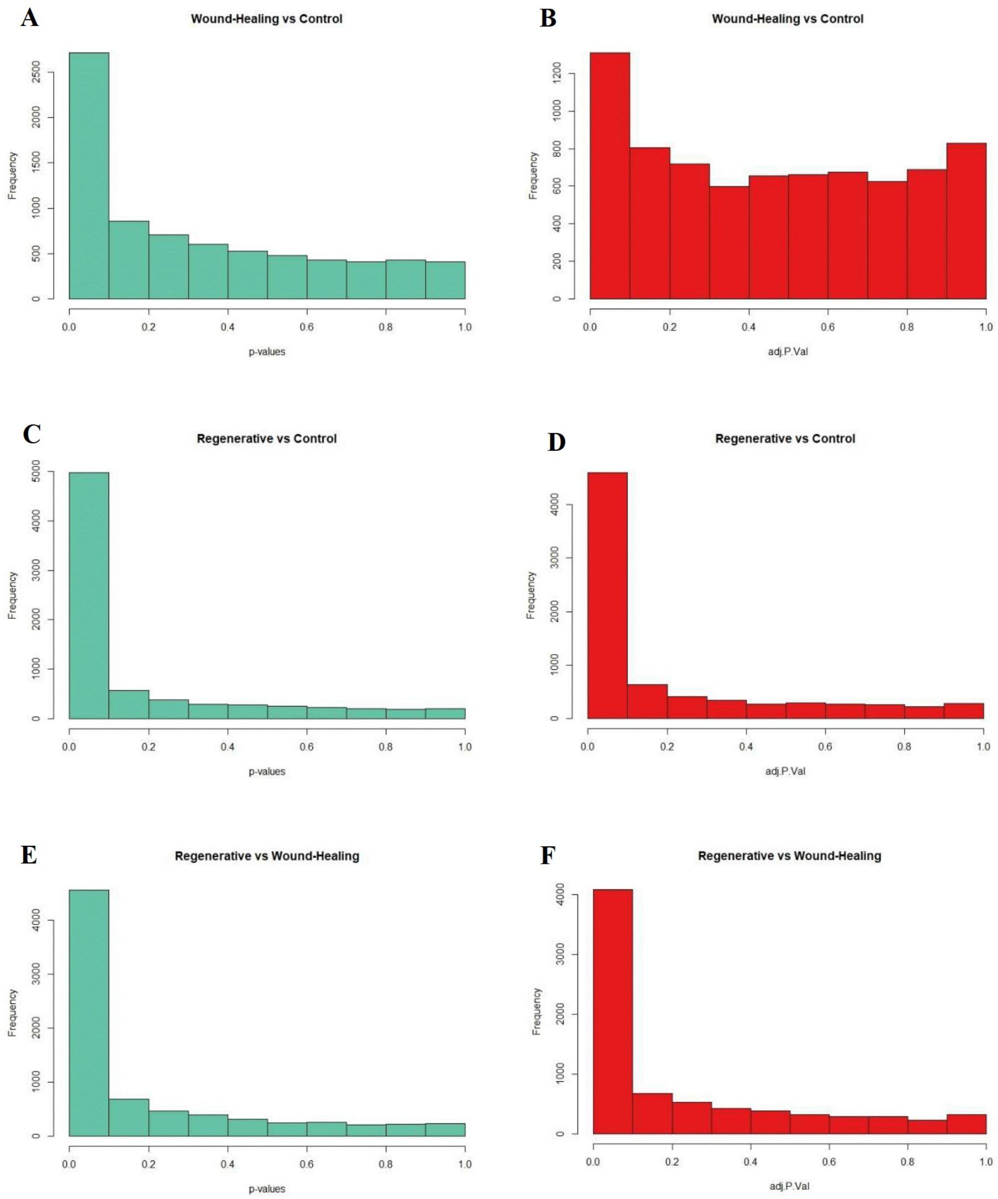

Supplementary Figure 6: Raw and adjusted p-values distribution of RNA-Seq DEA results. p-value histogram (left) and adjusted p-value histogram (right) were plotted after DEA on RNA-Seq data for the following comparisons A,B) wound healing vs. control, C,D) regenerative vs. control, E,F) regenerative vs. wound healing.

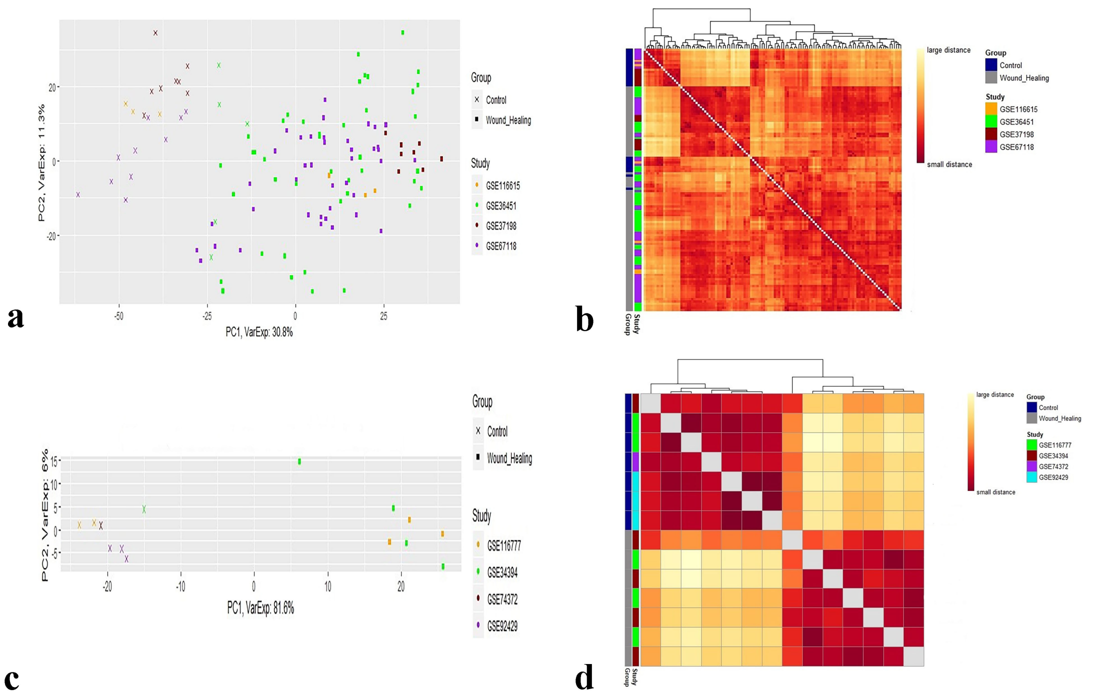

Supplementary Figure 7: DEA-based principal component analysis and sample-to-sample clustering heatmaps for wound healing vs. control comparison of A,B) Microarray quantile-normalized, log2-transformed, z-scored data after DEA (2,092 DE genes, 119 samples) and of C,D) RNA-seq logCPM (voom) counts data after DEA (423 DE genes, 14 samples). A,C) principle component analysis. B,D) sample-to-sample clustering heatmap. The number of samples per group are; 26 control and 93 wound healing for microarray data; 7 control and 7 wound healing for RNA-Seq data. The DE genes have an adjusted p-value < 0.01.

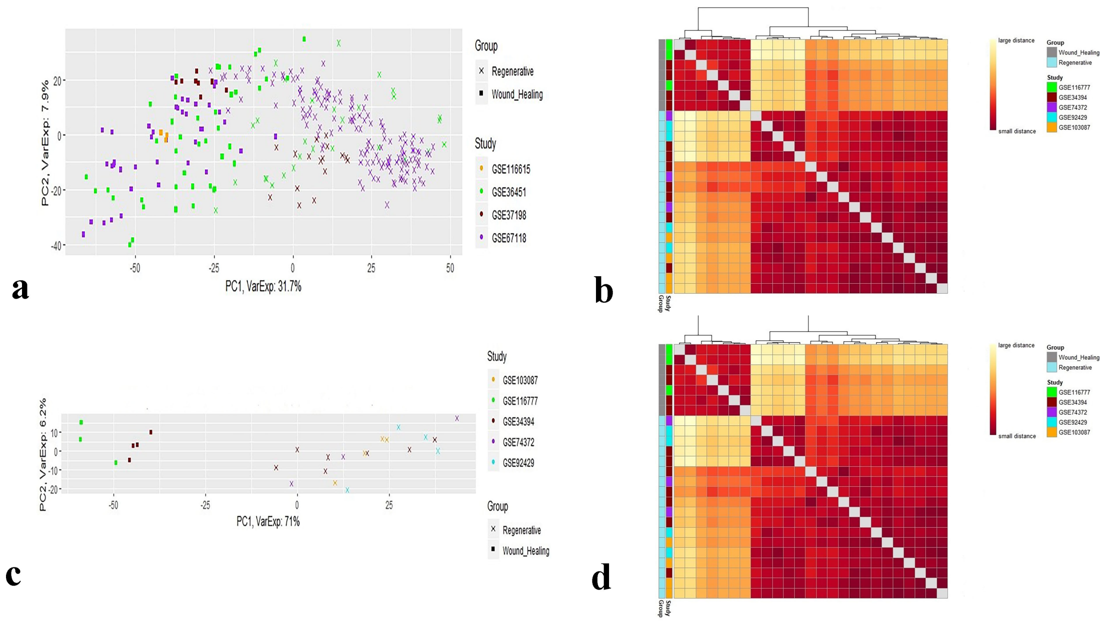

Supplementary Figure 8: DEA-based principal component analysis and sample-to-sample clustering heatmaps for wound healing vs. control comparison of A,B) Microarray quantile-normalized, log2-transformed, z-scored data after DEA (3,166 DE genes, 287 samples) and of C,D) RNA-seq logCPM (voom) counts data after DEA (2,371 DE genes, 25 samples)**.** A,C) principle component analysis. B,D) sample-to-sample clustering heatmap. The number of samples per group are; 194 regenerative and 93 wound healing for microarray data; 18 regenerative and 7 wound healing for RNA-Seq data. The DE genes have an adjusted p-value < 0.01.

Supplementary Table 1: Description of the selected microarray datasets for integrative analysis

GSE Accession	Year	Title	Publication	# of samples used for analysis	Total # of samples
GSE116615	2018	Comparative Transcriptomics of Limb Regeneration: Identification of Conserved Expression Changes Among Three Species of Ambystoma [microarray]	Dwaraka VB, Smith JJ, Woodcock MR, Voss SR. Comparative transcriptomics of limb regeneration: Identification of conserved expression changes among three species of Ambystoma. Genomics 2018 Aug 6. PMID: 30092345	6	6
GSE67118	2015	Gene Expression During the First 28 Days of Axolotl Limb Regeneration	Voss SR, Palumbo A, Nagarajan R, Gardiner DM et al. Gene expression during the first 28 days of axolotl limb regeneration I: Experimental design and global analysis of gene expression. Regeneration (Oxf) 2015 Jun;2(3):120-136. PMID: 27168937	198	198
GSE36451	2013	Comparative Transcriptional Profiling of the Axolotl Limb Identifies a Tripartite Regeneration-Specific Gene Program	Knapp D, Schulz H, Rascon CA, Volkmer M et al. Comparative transcriptional profiling of the axolotl limb identifies a tripartite regeneration-specific gene program. PLoS One 2013;8(5):e61352. PMID: 23658691	77	80
GSE37198	2012	Expression data from aquatic axolotl full thickness epithelial flank wounds, innervated limbs, and denervated limbs collected over seven days	James R. Monaghan, Antony Athippozhy, Ashley W. Seifert, Sri Putta, Arnold J. Stromberg, Malcolm Maden, David M. Gardiner, and S. Randal Voss. Gene expression patterns specific to the regenerating limb of the Mexican axolotl. Biology Open 2012 BIO20121594; Advance Online Publication August 8, 2012, doi:10.1242/bio.20121594.	32	44

GSE Accession	Platform	Study design	Type of raw/processed data used for analysis	Link GEO

GSE116615	GPL25286 [AMBY_002a520748F] Affymetrix Ambystoma mexicanum	Forelimbs were amputated mid-zeugopod. 1.0mm samples of heterogeneous tissue from regenerating limb tip were removed and used for RNA extraction. Three replicates samples were collected for each time point, with each replicate formed by pooling tissue from 5 different larvae.	CEL	https://www.ncbi.nlm.nih.gov/geo/query/acc.cgi?acc=GSE116615
GSE67118	GPL15153 Affymetrix Ambystoma mexicanum AMBY_002 20k array [CDF: AMBY_002a520748F]	The distal 1.0mm of heterogeneous tissue from regenerating limb tip were removed and used for RNA extraction. 10 samples were collected for each of 20 time points.	CEL	https://www.ncbi.nlm.nih.gov/geo/query/acc.cgi?acc=GSE67118
GSE36451	GPL15342 Agilent-019788 Ambystoma mexicanum 44k_v3_20080327	Regeneration of the axolotl forelimb lower arm was compared with the healing of a deep lateral injury in a high density timecourse (uncut, 3h, 6h, 9h, 12h, 24h, 36h, 52h, 72h, 120h, 168h, 288h and 528h after injury). Three independent biological replicates were performed using separate clutches of animals. Amputated and lateral wound samples were made as matched contralateral samples of four pooled animals per timepoint.	TXT	https://www.ncbi.nlm.nih.gov/geo/query/acc.cgi?acc=GSE36451
GSE37198	GPL15153 Affymetrix Ambystoma mexicanum AMBY_002 20k array [CDF: AMBY_002a520748F]	To determine the gene expression changes that take place during limb regeneration, flank wound healing, and an denervated amputated limb. Epidermal tissue and cells adhered to the epidermis were collected as samples. Two harvested samples was pooled for each animal. Four biological replicates were collected from uninjured epidermis (D0) and at 1, 3, and 7 days post injury.	CEL	https://www.ncbi.nlm.nih.gov/geo/query/acc.cgi?acc=GSE37198

Supplementary Table 2: Description of the selected RNA-Seq datasets for integrative analysis

GSE Accession	Year	Title	Publication	# of samples used for analysis	Total # of samples
GSE116777	2018	Comparative Transcriptomics of Limb Regeneration:Identification of Conserved Expression Changes Among Three Species of Ambystoma [RNA-Seq]	Dwaraka VB, Smith JJ, Woodcock MR, Voss SR. Comparative transcriptomics of limb regeneration: Identification of conserved expression changes among three species of Ambystoma. Genomics 2018 Aug 6. PMID: 30092345	5	18
GSE103087	2017	Identification of regenerative roadblocks via repeat deployment of limb regeneration in axolotls	Donald M. Bryant, Konstantinos Sousounis, Duygu Payzin-Dogru, Sevara Bryant, Aaron Gabriel W. Sandoval, Jose Martinez Fernandez, Rachelle Mariano, Rachel Oshiro, Alan Y. Wong, Nicholas D. Leigh, Kimberly Johnson & Jessica L. Whited. Identification of regenerative roadblocks via repeat deployment of limb regeneration in axolotls. npj Regenerative Medicine, 2, 30 (2017). doi:10.1038/s41536-017-0034-z	4	8
GSE92429	2016	A tissue-mapped axolotl de novo transcriptome enables identification of limb regeneration factors	Bryant DM, Johnson K, DiTommaso T, Tickle T et al. A Tissue-Mapped Axolotl De Novo Transcriptome Enables Identification of Limb Regeneration Factors. Cell Rep 2017 Jan 17;18(3):762-776. PMID: 28099853	7	42
GSE74372	2016	Small RNA and mRNA expression profiling during axolotl forelimb regeneration	King BL, Yin VP. A Conserved MicroRNA Regulatory Circuit Is Differentially Controlled during Limb/Appendage Regeneration. PLoS One 2016;11(6):e0157106. PMID: 27355827	4	8
GSE34394	2013	Three Distinct Phases of Regeneration-Specific Gene Expression in the Axolotl Blastema	Stewart R, Rascón CA, Tian S, Nie J et al. Comparative RNA-seq analysis in the unsequenced axolotl: the oncogene burst highlights early gene expression in the blastema. PLoS Comput Biol 2013;9(3):e1002936. PMID: 23505351	12	20

GSE Accession	Platform	Study design	Type of raw data used for analysis	GEO Link	ENA Link
GSE116777	GPL21473 Illumina HiSeq 2000 (Ambystoma mexicanum)	Forelimbs were amputated mid-zeugopod in all animals. 1.0mm samples of heterogeneous tissue from regenerating limb tips were removed and used for RNA extraction. Three replicates samples were collected for	Fastq	https://www.ncbi.nlm.nih.gov/geo/query/acc.cgi?acc=GSE116777	https://www.ebi.ac.uk/ena/data/view/PRJNA480225

GSE103087	GPL22800 Illumina HiSeq 2500 (Ambystoma mexicanum)	There are two conditions, with four biological replicates per condition. Condition 1 samples, "Multi-amp" in the sample names, are derived from axolotl limbs that have undergone six rounds of amputation-regeneration and mRNA was extracted at three days following the last amputation. Condition 2 samples, "Contol" in the sample names, are age- and size-matched sibling animals that have undergone a single amputation and mRNA was extracted at three days following this amputation.	Fastq	https://www.ncbi.nlm.nih.gov/geo/query/acc.cgi?acc=GSE103087	https://www.ebi.ac.uk/ena/data/view/PRJNA400170
GSE92429	GPL22800 Illumina HiSeq 2500 (Ambystoma mexicanum)	Profiled 42 samples across 16 tissues, from distinct locations among intact and regenerating limbs, relevant cell types, and other progenitor-rich samples using RNA-sequencing	Fastq	https://www.ncbi.nlm.nih.gov/geo/query/acc.cgi?acc=GSE92429	https://www.ebi.ac.uk/ena/data/view/PRJNA300706
GSE74372	GPL14997 Illumina Genome Analyzer IIx (Ambystoma mexicanum)	Characterized microRNA expression during Axolotl forelimb regeneration using small RNA sequencing. The same samples were assayed for mRNA expression using mRNA sequencing. Small RNA and mRNA gene expression profiling during 0, 3, 6 and 14 days post amputation.	Fastq	https://www.ncbi.nlm.nih.gov/geo/query/acc.cgi?acc=GSE74372	https://www.ebi.ac.uk/ena/data/view/PRJNA299879
GSE34394	GPL14997 Illumina Genome Analyzer IIx (Ambystoma mexicanum)	performed deep RNA sequencing of the blastema over a time course.Then, compare the expression patterns to those in a mouse digit amputation model to identify genes specific to the regenerative response	Fastq	https://www.ncbi.nlm.nih.gov/geo/query/acc.cgi?acc=GSE34394	https://www.ebi.ac.uk/ena/data/view/PRJNA149573

Supplementary Table 3: Detailed information on possible GSM accession, timepoint, amputation/injury site, and replicate number of each sample from Microarray data as repoted in GEO

**GSE Study**	**1. Control**: Intact (forelimb or hindlimb)-zero time point
	GSM3243805 forelimb 0hpa replicate 1
GSE116615	GSM3243806 forelimb 0hpa replicate 2
	GSM3243807 forelimb 0hpa replicate 3
	GSM1639337 forelimb T0 replicate 1
	GSM1639338 forelimb T0 replicate 2
	GSM1639339 forelimb T0 replicate 3
	GSM1639340 forelimb T0 replicate 4
GSE67118	GSM1639341 forelimb T0 replicate 5
	GSM1639342 forelimb T0 replicate 6
	GSM1639343 forelimb T0 replicate 7
	GSM1639344 forelimb T0 replicate 8
	GSM1639345 forelimb T0 replicate 9
	GSM1639346 forelimb T0 replicate 10
	GSM893962 mat-1_rep1
	GSM893963 mat-2_rep1
GSE36451	GSM894016 mat-2_rep3
	GSM894015 mat-1_rep3
	GSM893989 mat_rep2
	GSM913369 Flank wound at D0, biological rep1
	GSM913370 Flank wound at D0, biological rep2
	GSM913371 Flank wound at D0, biological rep3
GSE37198	GSM913372 Flank wound at D0, biological rep4
	GSM913373 Innervated limb at D0, biological rep1
	GSM913374 Innervated limb at D0, biological rep2
	GSM913375 Innervated limb at D0, biological rep3
	GSM913376 Innervated limb at D0, biological rep4
**GSE Study**	**2. Wound Healing**: injuries or amputations up to 48 hrs
	GSM3243808 forelimb 24hpa replicate 1
GSE116615	GSM3243809 forelimb 24hpa replicate 2
	GSM3243810 forelimb 24hpa replicate 3
	GSM1639347 forelimb T0.5 replicate 1
	GSM1639348 forelimb T0.5 replicate 2
	GSM1639349 forelimb T0.5 replicate 3
	GSM1639350 forelimb T0.5 replicate 4
	GSM1639351 forelimb T0.5 replicate 5
	GSM1639352 forelimb T0.5 replicate 6
	GSM1639353 forelimb T0.5 replicate 7
	GSM1639354 forelimb T0.5 replicate 8
	GSM1639355 forelimb T0.5 replicate 9
	GSM1639356 forelimb T0.5 replicate 10
	GSM1639357 forelimb T1 replicate 1
	GSM1639358 forelimb T1 replicate 2
	GSM1639359 forelimb T1 replicate 3
	GSM1639360 forelimb T1 replicate 4
	GSM1639361 forelimb T1 replicate 5
	GSM1639362 forelimb T1 replicate 6

	GSM1639363 forelimb T1 replicate 7
	GSM1639364 forelimb T1 replicate 8
	GSM1639365 forelimb T1 replicate 9
GSE67118	GSM1639366 forelimb T1 replicate 10
	GSM1639367 forelimb T1.5 replicate 1
	GSM1639368 forelimb T1.5 replicate 2
	GSM1639369 forelimb T1.5 replicate 3
	GSM1639370 forelimb T1.5 replicate 4
	GSM1639371 forelimb T1.5 replicate 5
	GSM1639372 forelimb T1.5 replicate 6
	GSM1639373 forelimb T1.5 replicate 7
	GSM1639374 forelimb T1.5 replicate 8
	GSM1639375 forelimb T1.5 replicate 9
	GSM1639376 forelimb T1.5 replicate 10
	GSM1639377 forelimb T2 replicate 1
	GSM1639378 forelimb T2 replicate 2
	GSM1639379 forelimb T2 replicate 3
	GSM1639380 forelimb T2 replicate 4
	GSM1639381 forelimb T2 replicate 5
	GSM1639382 forelimb T2 replicate 6
	GSM1639383 forelimb T2 replicate 7
	GSM1639384 forelimb T2 replicate 8
	GSM1639385 forelimb T2 replicate 9
	GSM1639386 forelimb T2 replicate 10
	GSM893964 3h_amp_rep1
	GSM893965 6h_amp_rep1
	GSM893966 9h_amp_rep1
	GSM893967 12h_amp_rep1
	GSM893968 24h_amp_rep1
	GSM893969 36h_amp_rep1
	GSM893970 52h_amp_rep1
	GSM893990 3h_amp_rep2
	GSM893991 6h_amp_rep2
	GSM893992 9h_amp_rep2
	GSM893993 12h_amp_rep2
	GSM893994 24h_amp_rep2
	GSM893995 36h_amp_rep2
	GSM893996 52h_amp_rep2
	GSM894017 3h_amp_rep3
	GSM894018 6h_amp_rep3
	GSM894019 9h_amp_rep3
	GSM894020 12h_amp_rep3
	GSM894021 24h_amp_rep3
	GSM894022 36h_amp_rep3
GSE36451	GSM894023 52h_amp_rep3
	GSM893976 3h_lwp_rep1
	GSM893977 6h_lwp_rep1
	GSM893978 9h_lwp_rep1
	GSM893979 12h_lwp_rep1
	GSM893980 24h_lwp_rep1

	GSM893981 36h_lwp_rep1
	GSM893982 52h_lwp_rep1
	GSM894002 3h_lwp_rep2
	GSM894003 6h_lwp_rep2
	GSM894004 9h_lwp_rep2
	GSM894005 12h_lwp_rep2
	GSM894006 24h_lwp_rep2
	GSM894007 36h_lwp_rep2
	GSM894008 52h_lwp_rep2
	GSM894029 3h_lwp_rep3
	GSM894030 6h_lwp_rep3
	GSM894031 9h_lwp_rep3
	GSM894032 12h_lwp_rep3
	GSM894033 24h_lwp_rep3
	GSM894034 36h_lwp_rep3
	GSM894035 52h_lwp_rep3
	GSM913381 Innervated limb at D1, biological rep1
	GSM913382 Innervated limb at D1, biological rep2
	GSM913383 Innervated limb at D1, biological rep3
GSE37198	GSM913384 Innervated limb at D1, biological rep4
	GSM913385 Flank wound at D1, biological rep1
	GSM913386 Flank wound at D1, biological rep2
	GSM913387 Flank wound at D1, biological rep3
	GSM913388 Flank wound at D1, biological rep4
**GSE Study**	**3. Regeneration**: amputations later than 48 hrs
	GSM1639387 forelimb T3 replicate 1
	GSM1639388 forelimb T3 replicate 2
	GSM1639389 forelimb T3 replicate 3
	GSM1639390 forelimb T3 replicate 4
	GSM1639391 forelimb T3 replicate 5
	GSM1639392 forelimb T3 replicate 6
	GSM1639393 forelimb T3 replicate 7
	GSM1639394 forelimb T3 replicate 8
	GSM1639395 forelimb T3 replicate 9
	GSM1639396 forelimb T3 replicate 10
	GSM1639397 forelimb T4 replicate 1
	GSM1639398 forelimb T4 replicate 2
	GSM1639399 forelimb T4 replicate 3
	GSM1639400 forelimb T4 replicate 4
	GSM1639401 forelimb T4 replicate 5
	GSM1639402 forelimb T4 replicate 6
	GSM1639403 forelimb T4 replicate 7
	GSM1639404 froelimb T4 replicate 8
	GSM1639405 forelimb T4 replicate 9
	GSM1639406 forelimb T4 replicate 10
	GSM1639407 forelimb T5 replicate 1
	GSM1639408 forelimb T5 replicate 2
	GSM1639409 forelimb T5 replicate 3
	GSM1639410 forelimb T5 replicate 4
	GSM1639411 forelimb T5 replicate 5

	GSM1639412 forelimb T5 replicate 6
	GSM1639413 forelimb T5 replicate 7
	GSM1639414 forelimb T5 replicate 8
	GSM1639415 forelimb T5 replicate 9
	GSM1639416 forelimb T5 replicate 10
	GSM1639417 forelimb T7 replicate 1
	GSM1639418 forelimb T7 replicate 2
	GSM1639419 forelimb T7 replicate 3
	GSM1639420 forelimb T7 replicate 4
	GSM1639421 forelimb T7 replicate 5
	GSM1639422 forelimb T7 replicate 6
	GSM1639423 forelimb T7 replicate 7
	GSM1639424 forelimb T7 replicate 8
	GSM1639425 forelimb T7 replicate 9
	GSM1639426 forelimb T7 replicate 10
	GSM1639427 forelimb T9 replicate 1
	GSM1639428 forelimb T9 replicate 2
	GSM1639429 forelimb T9 replicate 3
	GSM1639430 forelimb T9 replicate 4
	GSM1639431 forelimb T9 replicate 5
	GSM1639432 forelimb T9 replicate 6
	GSM1639433 forelimb T9 replicate 7
	GSM1639434 forelimb T9 replicate 8
	GSM1639435 forelimb T9 replicate 9
	GSM1639436 forelimb T9 replicate 10
	GSM1639437 forelimb T10 replicate 1
	GSM1639438 forelimb T10 replicate 2
	GSM1639439 forelimb T10 replicate 3
	GSM1639440 forelimb T10 replicate 4
	GSM1639441 forelimb T10 replicate 5
	GSM1639442 forelimb T10 replicate 6
	GSM1639443 forelimb T10 replicate 7
	GSM1639444 forelimb T10 replicate 8
	GSM1639445 forelimb T10 replicate 9
	GSM1639446 forelimb T10 replicate 10
	GSM1639447 forelimb T12 replicate 1
	GSM1639448 forelimb T12 replicate 2
	GSM1639449 forelimb T12 replicate 3
	GSM1639450 forelimb T12 replicate 4
	GSM1639451 forelimb T12 replicate 5
	GSM1639452 forelimb T12 replicate 6
	GSM1639453 forelimb T12 replicate 7
	GSM1639454 forelimb T12 replicate 8
	GSM1639455 forelimb T12 replicate 9
	GSM1639456 forelimb T12 replicate 10
	GSM1639457 forelimb T14 replicate 1
	GSM1639458 forelimb T14 replicate 2
	GSM1639459 forelimb T14 replicate 3
	GSM1639460 forelimb T14 replicate 4
GSE67118	GSM1639461 forelimb T14 replicate 5

GSM1639462 forelimb T14 replicate 6
GSM1639463 forelimb T14 replicate 7
GSM1639464 forelimb T14 replicate 8
GSM1639465 forelimb T14 replicate 9
GSM1639466 forelimb T14 replicate 10
GSM1639467 forelimb T16 replicate 1
GSM1639468 forelimb T16 replicate 2
GSM1639469 forelimb T16 replicate 3
GSM1639470 forelimb T16 replicate 4
GSM1639471 forelimb T16 replicate 5
GSM1639472 forelimb T16 replicate 6
GSM1639473 forelimb T16 replicate 7
GSM1639474 forelimb T16 replicate 8
GSM1639475 forelimb T16 replicate 9
GSM1639476 forelimb T18 replicate 1
GSM1639477 forelimb T18 replicate 2
GSM1639478 forelimb T18 replicate 3
GSM1639479 forelimb T18 replicate 4
GSM1639480 forelimb T18 replicate 5
GSM1639481 forelimb T18 replicate 6
GSM1639482 forelimb T18 replicate 7
GSM1639483 forelimb T18 replicate 8
GSM1639484 forelimb T18 replicate 9
GSM1639485 forelimb T18 replicate 10
GSM1639486 forelimb T20 replicate 1
GSM1639487 forelimb T20 replicate 2
GSM1639488 forelimb T20 replicate 3
GSM1639489 forelimb T20 replicate 4
GSM1639490 forelimb T20 replicate 5
GSM1639491 forelimb T20 replicate 6
GSM1639492 forelimb T20 replicate 7
GSM1639493 forelimb T20 replicate 8
GSM1639494 forelimb T20 replciate 9
GSM1639495 forelimb T20 replicate 10
GSM1639496 forelimb T22 replicate 1
GSM1639497 forelimb T22 replicate 2
GSM1639498 forelimb T22 replicate 3
GSM1639499 forelimb T22 replicate 4
GSM1639500 forelimb T22 replicate 5
GSM1639501 forelimb T22 replicate 6
GSM1639502 forelimb T22 replicate 7
GSM1639503 forelimb T22 replicate 8
GSM1639504 forelimb T22 replicate 9
GSM1639505 forelimb T22 replicate 10
GSM1639506 forelimb T24 replicate 1
GSM1639507 forelimb T24 replicate 2
GSM1639508 forelimb T24 replicate 3
GSM1639509 forelimb T24 replicate 4
GSM1639510 forelimb T24 replicate 5
GSM1639511 forelimb T24 replicate 6

	GSM1639512 forelimb T24 replicate 7
	GSM1639513 forelimb T24 replicate 8
	GSM1639514 forelimb T24 replicate 9
	GSM1639515 forelimb T24 replicate 10
	GSM1639516 forelimb T26 replicate 1
	GSM1639517 forelimb T26 replicate 2
	GSM1639518 forelimb T26 replicate 3
	GSM1639519 forelimb T26 replicate 4
	GSM1639520 forelimb T26 replicate 5
	GSM1639521 forelimb T26 replicate 6
	GSM1639522 forelimb T26 replicate 7
	GSM1639523 forelimb T26 replicate 8
	GSM1639524 forelimb T26 replicate 9
	GSM1639525 forelimb T26 replicate 10
	GSM1639526 forelimb T28 replicate 1
	GSM1639527 forelimb T28 replicate 2
	GSM1639528 forelimb T28 replicate 3
	GSM1639529 forelimb T28 replicate 4
	GSM1639530 forelimb T28 replicate 5
	GSM1639531 forelimb T28 replicate 6
	GSM1639532 forelimb T28 replicate 7
	GSM1639533 forelimb T28 replicate 8
	GSM1639534 forelimb T28 replicate 9
	GSM893971 72h_amp_rep1
	GSM893972 120h_amp_rep1
	GSM893973 168h_amp_rep1
	GSM893974 288h_amp_rep1
	GSM893975 528h_amp_rep1
	GSM893997 72h_amp_rep2
	GSM893998 120h_amp_rep2
	GSM893999 168h_amp_rep2
	GSM894000 288h_amp_rep2
	GSM894001 528h_amp_rep2
	GSM894024 72h_amp_rep3
	GSM894025 120h_amp_rep3
	GSM894026 168h_amp_rep3
	GSM894027 288h_amp_rep3
	GSM894028 528h_amp_rep3
GSE36451	GSM893983 72h_lwp_rep1
	GSM893984 120h_lwp_rep1
	GSM893985 168h_lwp_rep1
	GSM893986 288h_lwp_rep1
	GSM893987 528h_lwp_rep1
	GSM894009 72h_lwp_rep2
	GSM894010 120h_lwp_rep2
	GSM894011 168h_lwp_rep2
	GSM894012 288h_lwp_rep2
	GSM894013 528h_lwp_rep2
	GSM894036 72h_lwp_rep3
	GSM894037 120h_lwp_rep3

	GSM894038 168h_lwp_rep3
	GSM894039 288h_lwp_rep3
	GSM894040 528h_lwp_rep3
GSE37198	GSM913393 Innervated limb at D3, biological rep1
	GSM913394 Innervated limb at D3, biological rep2
	GSM913395 Innervated limb at D3, biological rep3
	GSM913396 Innervated limb at D3, biological rep4
	GSM913405 Innervated limb at D7, biological rep1
	GSM913406 Innervated limb at D7, biological rep2
	GSM913407 Innervated limb at D7, biological rep3
	GSM913408 Innervated limb at D7, biological rep4
	GSM913397 Flank wound at D3, biological rep1
	GSM913398 Flank wound at D3, biological rep2
	GSM913399 Flank wound at D3, biological rep3
	GSM913400 Flank wound at D3, biological rep4
	GSM913409 Flank wound at D7, biological rep1
	GSM913410 Flank wound at D7, biological rep2
	GSM913411 Flank wound at D7, biological rep3
	GSM913412 Flank wound at D7, biological rep4

Supplementary Table 4: Detailed information on possible GSM accession, timepoint, amputation/injury site, and replicate number of each sample from RNA-Seq data as reported in GEO

**GSE Study**	**1. Control**: Intact (forelimb or hindlimb)-zero time point
GSE116777	GSM3261803 SRV-0041 0hpa
	GSM3261804 SRV-0042 0hpa
GSE34394	GSM848198 Axolotl limb blastemal 0hr
GSE74372	GSM1919057 0dpa mRNA
GSE92429	GSM2429608 upperarm_10329_GTGAAA
	GSM2429609 upperarm_10326_ATGTCA
	GSM2429610 upperarm_10322_CAGATC
**GSE Study**	**2. Wound Healing**: injuries or amputations up to 48 hrs
GSE116777	GSM3261812 SRV-0044 24hpa
	GSM3261813 SRV-0045 24hpa
	GSM3261814 SRV-0046 24hpa
GSE34394	GSM848199 Axolotl limb blastemal 3hr
	GSM848200 Axolotl limb blastemal 6hr
	GSM848201 Axolotl limb blastemal 12hr
	GSM848202 Axolotl limb blastemal 1d
**GSE Study**	**3. Regeneration**: amputations later than 48 hrs
GSE34394	GSM848203 Axolotl limb blastemal 3d
	GSM848204 Axolotl limb blastemal 5d
	GSM848205 Axolotl limb blastemal 7d
	GSM848206 Axolotl limb blastemal 10d
	GSM848207 Axolotl limb blastemal 14d
	GSM848208 Axolotl limb blastemal 21d
	GSM848209 Axolotl limb blastemal 28d
GSE74372	GSM1919058 3dpa mRNA
	GSM1919059 6dpa mRNA
	GSM1919060 14dpa mRNA
GSE92429	GSM2429615 proximal_TGACCA_L008
	GSM2429616 proximal_CAGATC_L006
	GSM2429628 distal_GCCAAT_L006
	GSM2429629 distal_CGATGT_L008
GSE103087	GSM2753452 LIB014416_TRA00035334
	GSM2753453 LIB014416_TRA00035335
	GSM2753454 LIB014416_TRA00035336
	GSM2753455 LIB014416_TRA00035337

Supplementary Table 5. Primer list used in qRT-PCR

Primer name	Primer Sequence
EF1A forward	5'-CGCGAGTTCTAATAGGTTCTGATATT-3'
EF1A reverse	5'-ATGCAATTACTTTAGCGAGTACCAC-3'
MMP13 forward	5'-TGGAAGAAGACTGCGTTGA-3'
MMP13 reverse	5'-ACACTTTGAAGGCCCTTTG-3'
KRT17 forward	5'-CCAAGTGTTTTGCCCAGAAT-3'
KRT17 reverse	5'-GCCTGGAATCCAAGTCACAT-3'
F13A1 forward	5'-GGCCTCACTTCATTTCTTCG-3'
F13A1 reverse	5'-AGGCTTGGTGAACTCCACAC-3'
TGFB1 forward	5'-GGGGAACGGGTTCATATCAT-3'
TGFB1 reverse	5'-TCTCTGAATGAACTGGGATGG-3'
MAPK6 forward	5'-CAGTGGTGATGAGAGCTTCC-3'
MAPK6 reverse	5'-AAACGTGTCCAAGTAACTGGTG-3'
METTL21A forward	5'-CCTTCAACTTTGCCAGCCAT-3'
METTL21A reverse	5'-CAATCACAGCCAGCCTTTGT-3'
PCDHGC3 forward	5'-CGTTAATGTCCTGCACCTCC-3'
PCDHGC3 reverse	5'-CTGCTGGTGAACGAAAGAA-3'
IFNAR1 forward	5'-CACAGGACCAGCAGTTTTGT-3'
IFNAR1 reverse	5'-GACTGGTTTTCGGCTCTCTG-3'
CTPS forward	5'-GAACGGGGCCTCAAATTTGT-3'
CTPS reverse	5-'CCACTGAAGCTAGGAGGAGG-3'

Supplementary Table 6: **List of the top DE genes commonly identified by Microarray and RNA-seq data**

	Wound-Healing vs. Control	
**Symbol**	**logFC_Microarray**	**logFC_RNAseq**
MMP1	2,334361424	6,338005577
MMP3	2,613784506	6,326727984
MMP13	2,505942171	6,142324809
SERPINE1	2,33448261	5,499983443
DUSP5	1,900733092	5,461830138
SLC2A1	2,583964126	5,376636756
PTGS2	1,349113221	5,081598276
DUSP1	1,384630072	4,592839444
TCN1	2,6701994	4,586502325
MMP19	1,842536588	4,438045568
CYR61	1,524675274	4,323121128
TFPI2	2,577931481	4,213641401
JUNB	2,221756685	4,18203071
HMOX1	2,20457087	4,078211279
GADD45B	2,292961256	3,967008368
MPO	1,539231455	3,754991511
EGR1	1,183623733	3,741661794
PLBD1	1,849523153	3,690513073
TIMP1	2,651597185	3,640411179
TUBB6	2,145998088	3,51921625
ARG1	2,294109452	3,303208849
DGAT2	1,612931003	3,252060638
ANTXR2	1,844975053	3,223922826
SCG2	2,309067267	3,218333481
MMP2	1,166588789	3,157313017
DDIT4	1,689073456	3,148257645
THBS1	2,741572843	3,119318838
FGL2	1,603042676	3,109661211
ODC1	2,371673142	3,059811212
TNC	1,928430829	3,038263794
TGM1	2,211429497	3,001913471
SPSB4	1,643229066	2,987181157
CXCR4	1,345824199	2,844759447
SOCS1	1,979271742	2,757441497
CHIT1	1,50988719	2,735948537
SGK1	1,63809861	2,65765517
ARL4D	2,282158023	2,607778471
UGDH	2,534200029	2,581818431
GADD45G	1,684827707	2,561964354
GLUL	2,61045621	2,530108807
PHLDA2	1,707124853	2,483842377
OBFC2A	2,403141247	2,330756238
DLGAP4	2,594097381	2,257749664
CYBB	2,007559993	2,22545569

SMAD7	1,029288045	2,196126161
ZFAND5	2,153701831	2,183211463
RHOB	1,00919381	2,160984515
PPP1R14B	2,610945966	2,154678459
ARHGEF2	1,961960934	2,137000929
G0S2	1,41786803	2,11914662
MAT2A	1,194248207	2,109284438
UGCG	1,874832507	2,036689675
SQSTM1	1,277666454	2,001438828
MAPK6	2,591000214	1,915050242
LARP6	1,608874063	1,849560081
FAM110B	2,360501041	1,79436392
LGALS9	1,717625991	1,776964321
BMP2	1,548472922	1,703815048
SLC16A1	1,520238061	1,699210532
KIAA1737	1,741476731	1,685631249
SNX18	1,98102521	1,638333222
ZNRF1	2,012149822	1,633122284
ZYX	2,492173002	1,597628818
SEMA3F	1,079471461	1,544727872
SLC30A1	2,26847655	1,446118762
RND3	1,431083506	1,406607239
FAM108A1	1,115918282	1,391500219
BCL2L1	2,112853034	1,388564269
ZC3H11A	2,662591672	1,355679812
MXI1	1,350166451	1,346243753
TMEM63B	1,453640867	1,323548748
SLC20A1	1,035372778	1,314067927
CDKN1C	1,054397908	1,308419045
BASP1	1,178196837	1,297370601
KEAP1	1,333360173	1,295536181
ERO1L	2,473193008	1,274935767
CCNL1	1,141953243	1,265750639
CHD7	1,575588305	1,256585758
UBE2J1	1,535279	1,219634011
ACCS	-1,044734647	1,216692098
LMNB1	1,488273056	1,210726837
PELI1	1,655103458	1,20294839
SKI	1,282945387	1,093195775
ACTB	1,941734833	1,076181244
YWHAZ	1,987227655	1,071105742
TOP1	1,737250792	1,041115392
RRAS2	1,708469955	1,017611445
RMI1	-1,262418344	-1,029607406
CENPO	-1,042610701	-1,102455378
SLC25A12	-1,127310982	-1,310792154
ZNF367	-1,017944133	-1,411253607

	Regenerative vs. Control	
**Symbol**	**logFC_Microarray**	**logFC_RNAseq**
KAZALD1	1,60584757	7,606392103
MMP13	1,28751864	4,684782882
TNC	2,76899951	4,507054818
KRT17	3,059656959	4,433341211
PLBD1	1,335313996	3,405004145
TGFBI	1,65973202	3,250979687
PAMR1	1,629254917	3,170848422
F13A1	1,212030503	2,78544746
HIST3H2BB	1,226535435	2,752726323
LAMA1	1,304306191	2,670020033
CRABP2	1,922354318	2,519907225
SPSB4	1,255390854	2,378661784
MSI1	1,49324911	2,349822341
HAS2	1,086876414	2,327763108
MMP2	1,059033428	2,246131184
MAPK6	2,50893046	2,216563294
CXCR4	1,067219183	2,212249173
HMOX1	1,435034277	2,140113427
RCC1	1,305262112	2,114316205
MDK	1,084822228	2,110868401
TMEM35	1,227274134	2,093854739
CCNJ	1,613608596	2,024962826
THBS1	1,815119729	2,000311495
CALD1	1,755608975	1,986202794
B9D1	1,505093553	1,947534205
FAT1	1,066795372	1,877197689
ENTPD1	1,775105449	1,84689419
FN1	1,553184389	1,835956044
SMAD7	1,194322149	1,825610643
NEK2	1,02745336	1,813303252
KPNA2	1,427076531	1,813037336
MYO10	2,056775205	1,790043557
OBFC2A	1,71516334	1,776729504
COL12A1	1,074030507	1,763226013
KIF11	1,050111115	1,751192362
AURKA	1,072344938	1,742388078
ID3	1,697729526	1,7032721
FAM54A	1,257628986	1,687796398
SAFB	1,346742416	1,670871889
MBD3	1,328615086	1,655521106
PLK1	1,293625144	1,640839096
TFPI	1,169997882	1,633649003
JUNB	1,546643627	1,612122395
APEX1	1,513003124	1,592478909
BASP1	1,407396377	1,574297323

MTBP	1,341226826	1,558447412
RRM2	1,047933537	1,557171033
FEN1	1,260591674	1,548648846
CCDC112	1,488899459	1,547224799
HCFC1	1,091418951	1,543500584
KIF23	1,096763883	1,535794098
ETF1	1,907804983	1,523316608
TRIP13	1,329294518	1,519313346
RBMXL1	1,83538259	1,511374069
LOXL2	1,683217659	1,511148649
FUS	1,627373752	1,506398904
RPS6KB1	1,998922288	1,482751914
ANP32E	1,013212638	1,482296354
PPRC1	1,55319949	1,472802156
PCDHGC3	1,194529635	1,472670083
RRM1	1,186622498	1,466832234
KIF22	1,05279908	1,463444434
ANTXR2	1,157689592	1,455954835
CYBB	1,513514029	1,440987907
SLTM	1,839913086	1,423609321
DCTPP1	1,30169385	1,409175524
SLC37A2	1,2743202	1,407601065
CTHRC1	1,707279363	1,406558193
STMN1	1,044853214	1,39860325
TOP2B	1,315825097	1,398436393
MAD2L1	1,011679519	1,394810466
CSE1L	1,199614525	1,391365394
CNN2	1,458270201	1,379938557
BRD3	1,519527072	1,372571943
NOLC1	1,529308157	1,367652936
RHEBL1	1,216393753	1,360763512
PTBP1	1,3152891	1,35916821
TCF7L1	1,045569514	1,358976143
SULF1	1,663460656	1,347564214
POLR1C	1,133310331	1,310366692
TMPO	1,711141104	1,287855374
CLK3	1,702902437	1,28495491
IFNAR1	1,218780517	1,279103087
TUBB6	1,274342008	1,278964851
LGALS9	1,517271279	1,27866492
METTL9	1,64659179	1,276069061
MPHOSPH1(	1,188343856	1,275420823
HSPD1	1,170583865	1,267641238
CTPS	1,275947639	1,266660019
RRP9	1,171714652	1,264018926
RBM14	2,182063802	1,261296776
VKORC1L1	1,265380317	1,26102556

IQCB1	1,090665177	1,258186512
CBX3	2,00281647	1,249126017
AURKB	1,073467612	1,249033698
HELLS	1,006017115	1,248658128
NASP	1,211429737	1,243353349
DLGAP4	1,97032316	1,242348426
HNRNPM	2,129523597	1,239922224
RANBP1	2,053316532	1,233057498
TNFAIP1	1,474680898	1,230858043
TOP1	2,055539401	1,223524047
CDC20	1,222147319	1,217938645
SNRPA1	2,014592306	1,207469543
SNRPA	2,072271658	1,206671475
PLOD1	1,136888509	1,205371696
ABCF3	1,410062591	1,204513445
OSGIN2	1,679516823	1,201526877
TRA2B	1,863140128	1,201250461
DOCK7	1,171588948	1,199925219
BMP2	1,254383717	1,194350684
ODC1	1,429935845	1,188706636
MOSPD1	1,161348584	1,188387736
BAZ1B	1,067569702	1,18746786
RBM45	1,561955186	1,185808293
GART	1,512272912	1,177148744
HAUS1	1,061849766	1,1738079
RBMX	1,652647418	1,16925224
BICD2	1,135499607	1,168321326
ITGB1	1,120346445	1,167621019
HMGB2	1,574797886	1,164390471
MRTO4	1,713486271	1,162133672
PRC1	1,053242679	1,160453632
PWP2	1,276759182	1,15994105
ZRANB2	1,062857067	1,157887282
NIPBL	1,42940232	1,150073674
CHD7	1,250149626	1,14663182
FBL	1,719161401	1,146031477
EWSR1	1,674695735	1,141187012
FILIP1L	1,368991093	1,139620733
CTTNBP2NL	1,090690398	1,138005977
SFPQ	1,783635615	1,136416749
MFAP2	1,651450121	1,135887019
PRPF39	1,593177148	1,133896893
SNRNP40	1,845539179	1,132316844
THAP6	1,984875967	1,131816827
VIM	1,241452154	1,13048239
CDCA8	1,007398768	1,126471673
DHX15	1,798987664	1,125904559

PSPC1	1,98185145	1,123943803
MAD2L1BP	1,630793821	1,114912413
FLNA	1,826421336	1,11478458
TARDBP	2,028328068	1,113076138
NOL11	1,662138781	1,113013553
NUP160	1,112650114	1,104068885
SPATS2L	1,246248719	1,103367499
CIRH1A	1,370557103	1,101662692
PTCD3	1,481629023	1,100268844
NUP85	1,173534131	1,09527314
GPNMB	1,355905264	1,091575357
IPO11	1,703512458	1,09072166
COIL	1,125811926	1,087814235
ASF1B	1,045023567	1,082578269
PRR11	1,020036112	1,082327308
SART3	1,019727892	1,08211025
TMEM138	1,524606206	1,079523195
PGGT1B	1,349383345	1,078537287
ZNF862	1,575539173	1,077523498
MATR3	1,303474906	1,076468582
PAG1	1,236498991	1,075181881
ERO1L	2,249818466	1,071538641
PBRM1	1,416220211	1,071358301
JAG1	1,069589778	1,067033519
CTSC	1,025768569	1,064298507
METTL2A	1,898105627	1,062502764
RFC2	1,421526343	1,05409067
DKC1	1,666041292	1,047930323
USP1	1,018325585	1,045341957
CDV3	1,644914315	1,039617407
CSTF1	1,316345574	1,03723491
ZC3H11A	1,791644439	1,035901815
ZNF326	1,864956156	1,035226732
PRPF3	1,327773993	1,033609437
RPA2	1,003318854	1,031864745
TNPO3	1,278150791	1,02788666
SULT6B1	1,716591067	1,024087011
CLNS1A	1,466906716	1,009842298
SMARCC1	1,322774945	1,008908149
MCM3AP	1,192612513	1,008655458
UBE2I	1,370939224	1,004987207
MYEF2	1,680966286	1,003795741
ECHDC2	-1,296186257	-1,001090731
ROGDI	-1,107985429	-1,013062883
SNTA1	-1,843783592	-1,014938705
AIFM3	-1,398100987	-1,015427986
HLCS	-1,423441959	-1,026973484

ANKRD39	-1,425575027	-1,034801098
DCAF6	-2,213102194	-1,035050897
ATP5S	-1,833190149	-1,050563854
SYNJ2BP	-1,931695181	-1,051549547
PRDX1	1,05533778	-1,051735053
COX17	-1,751697447	-1,053856088
HINT2	-1,569588648	-1,078275286
COX7B	-1,819455822	-1,082756245
GOT1	-2,20425545	-1,112275321
PPP1R14A	-2,037135446	-1,131287547
VDAC1	-2,140958681	-1,133831928
ATP5G2	-1,693341879	-1,138098264
PARK7	-2,279856235	-1,138136014
GJB5	1,111139222	-1,141513261
ATP5I	-1,840790474	-1,147153062
TMSB15A	1,535796427	-1,15018179
GOT2	-1,361735323	-1,168972881
ACAT1	-1,752602147	-1,173036185
CECR1	-1,188084813	-1,186173261
PCGF5	-1,929122719	-1,195874295
MDH2	-2,332714762	-1,205988654
ITGB1BP3	-1,566275694	-1,208668611
ENPP5	-1,785744521	-1,220037704
ACADL	-1,803302639	-1,221774974
FKBP11	-1,394967118	-1,234980211
ACBD7	-1,140081654	-1,248099782
CD74	-1,402358357	-1,258144735
ANXA6	-1,531475071	-1,260928833
HEXB	-1,000828977	-1,280598421
PNO1	-1,80293508	-1,2826575
COX7A2L	-2,015605025	-1,30136179
BNIP3	-1,595705382	-1,328005352
SPA17	-1,020822579	-1,338175559
PKIG	-1,528432567	-1,34829085
RHAG	-1,02531363	-1,357239225
OAT	-2,49899907	-1,361631261
GABARAPL1	-1,23306412	-1,393249041
GYG1	-1,879544138	-1,414170209
GMPR	-2,075239994	-1,438041395
PGK1	-1,581851511	-1,445834828
GSTM4	-1,512452641	-1,451709935
FBLN5	-1,436611202	-1,492974251
PRR5	-2,115305255	-1,49514443
PRKAG2	-2,225927263	-1,509808663
ARHGAP18	-1,391679111	-1,509833552
EPAS1	-1,203717355	-1,510341272
COL4A1	-1,48759009	-1,517665758

SNTB1	-1,443965824	-1,539902939
COL1A1	-1,315496356	-1,551555892
FXYD1	-1,387079467	-1,570029071
IFI44L	-1,337303365	-1,591085899
MAP1LC3A	-1,048442385	-1,608903594
HEBP2	-1,16081149	-1,609816913
PPL	-1,426828953	-1,634860821
SORBS1	-1,469246802	-1,658002896
PEBP1	-1,748231343	-1,66451815
COL4A2	-1,578907558	-1,688522063
GPI	-1,069493774	-1,691381112
SERPINF1	-1,397281963	-1,696157991
CCL5	-1,109553031	-1,772032858
PGM1	-1,759154524	-1,789645729
PLAC9	-1,23178362	-1,792566557
ABAT	-1,372913922	-1,800724993
BIN1	-1,857033315	-1,805251946
PPARG	-1,366697216	-1,829973349
CCNG1	-1,817819725	-1,851649127
UPK3A	-1,480318153	-1,858910264
TPI1	-1,69430135	-1,888377751
CLEC3B	-1,531598746	-1,893222639
GLDC	-1,564249032	-1,912452571
MYH10	-1,575120031	-1,93208765
LRRC20	-2,089373822	-1,933362298
PMP22	-1,226902428	-1,973445497
GAPDH	-1,236287905	-1,97506573
GPD1	-1,821074145	-1,975225236
COL8A1	-1,382043145	-1,984775861
ASPH	-1,840188512	-1,985255629
SLC25A12	-2,19802149	-2,007739273
PABPC4	-1,459162145	-2,011856772
PIP5K1B	-1,131494653	-2,02168383
DPT	-1,308123183	-2,022602555
B2M	-1,105216673	-2,047815602
MR1	-1,140217793	-2,048429498
WFDC2	-1,795908237	-2,084519667
FMO3	-1,456471792	-2,221183265
TPPP3	-1,009791224	-2,223361269
CPT1A	-1,357922073	-2,23155879
GAMT	-1,826047131	-2,231630856
SATB1	-1,227075624	-2,273040443
TNNT3	-1,513997901	-2,339849816
PDGFRL	-1,800141675	-2,371839524
NFKBIE	-1,372821247	-2,402588329
RBP4	-1,103940731	-2,459892494
IL20RB	-1,010991128	-2,561217059

CSPG5	-2,141462953	-2,581255631
OXCT1	-1,869403588	-2,588758755
PSMB8	-1,620828601	-2,592896248
TPM3	-1,474841223	-2,617865293
CSRP2	-1,274203037	-2,620124343
ATP1B2	-1,072050551	-2,634122381
PON2	-1,954705364	-2,645227945
PYGM	-2,102987141	-2,650635686
SLC7A11	-1,868178941	-2,730479253
SLC2A9	-1,354612735	-2,748597191
LAMB2	-1,915486057	-2,857923058
CA2	-1,153542882	-2,917604918
FAM177B	-1,23535219	-2,920998358
MFAP5	-1,544481335	-2,960164026
FHL1	-1,456701548	-3,041851356
KRT6A	-1,037350883	-3,14055249
ACTA2	-1,56258874	-3,241846572
ALDH1A1	-1,427074822	-3,260108361
HSPB8	-1,759665694	-3,32357136
KRT5	1,276836757	-3,348271133
ITLN1	-1,012268432	-3,421464369
UNC45B	-2,001763417	-3,473905388
BHMT	-1,619235379	-3,492767861
RHCG	-1,117440496	-3,497517836
ADH4	-1,879124268	-3,533277119
XDH	-1,00091285	-3,640309512
CHRDL1	-1,350119239	-3,736435992
AGMAT	-1,518843667	-3,73840116
PTPLA	-1,719731661	-3,796454185
ADSSL1	-1,124744305	-3,866619431
GATM	-1,658542074	-3,886763213
TPM1	-1,398433837	-4,007238876
DES	-1,342677011	-4,509259662
MYL4	-1,743138709	-4,523873064
PFKM	-1,687934512	-4,553956394
CRISPLD2	-1,034256986	-4,557077964
KBTBD5	-1,215525597	-4,567147531
SMTNL2	-1,747093833	-4,757551504
INMT	-1,506190488	-4,824161123
PRPH	-1,066754349	-4,904548186
AK1	-1,844438209	-5,176738766
CRYAB	-1,678744326	-5,240546744
CACNG1	-1,30492096	-5,323867555
SMYD1	-1,814332152	-5,73188589
FAAH	-1,608238464	-5,754611085
SLC25A4	-1,24751953	-5,77807223
MYBPC3	-1,589077946	-5,896018351

MYH1	-1,498601798	-6,279124018
MYH4	-1,422995109	-6,515871214
TNNI1	-1,493765717	-6,527468247
MBP	-1,621325411	-6,653087835
MYH7	-1,297431291	-6,678383953
MYH2	-1,534959699	-6,727767502
TNNT1	-1,510615817	-6,875421716
MYH13	-1,244720679	-6,97257457
MYL2	-1,366521528	-7,105320813
KBTBD10	-1,500898816	-7,193339546
TNNI2	-1,369660522	-7,228379118
TNNC2	-1,478950273	-7,534047748
TNNC1	-1,580532451	-7,755150972
MYL3	-1,490827629	-7,786931108
ACTA1	-1,25025588	-7,871633305
MYL1	-1,235456453	-7,899108953
ACTC1	-1,509585867	-7,945791168
ACTN3	-1,894071237	-8,043620356
MYBPC2	-1,549730099	-8,198535133
ADIPOQ	-1,647000481	-8,213830687
CKM	-1,288476705	-8,420752802
MYOZ1	-1,781804064	-8,639080141
MYLPF	-1,226578963	-9,33208102
PVALB	-1,246047342	-9,578303621

Regenerative vs. Wound-Healing

Symbol	logFC_Microarray	logFC_RNAseq
LAMA1	1,681575881	3,107642499
FBLN1	1,095020252	2,753739025
CCNB1	1,63255645	2,571265222
CCNB3	1,546283843	2,504766175
PCDH18	1,053955619	2,41058588
AURKA	1,566892534	2,404717856
MSI1	1,225454798	2,390891775
TGFBI	1,406601655	2,348763563
MAD2L1	1,632499335	2,231539566
NEK2	1,24166499	2,174606905
KIFC1	1,701683946	2,162698956
CDCA7L	1,018397291	2,157764912
MCM3	1,269945481	2,139733989
PLK1	1,498920574	2,122319124
KIAA0101	1,604164713	2,117462647
SMC2	1,317620085	2,107918358
KIF11	1,488806204	2,102939225
KIF22	1,619286638	2,06396245
ST8SIA2	1,251549538	2,029740373
SAFB	1,605975885	1,999124019
TPX2	1,671174325	1,983177803

FAM54A	1,341149047	1,975071617
STMN1	1,760230349	1,943535247
ZNF367	1,055022489	1,936582169
CDCA5	1,54808286	1,934198947
UHRF1	1,34193544	1,923651937
CRABP2	1,214411766	1,912998733
KIF20A	1,589458139	1,902278368
BRD3	1,11809543	1,892648667
RCC1	1,176652784	1,87015458
CDCA8	1,616120254	1,844058795
CENPF	1,450620796	1,840154909
AURKB	1,751490107	1,833971373
CHEK1	1,110910967	1,822517304
PRC1	1,382568795	1,807828188
CCNA2	1,588922896	1,793986499
MTBP	1,056317552	1,789952822
HAUS1	1,475865613	1,769786394
HIST1H2BJ	1,303769005	1,765956358
FEN1	1,446256135	1,756839918
KIF23	1,527935828	1,719857591
RAD51AP1	1,41138275	1,718392642
MCM2	1,205625785	1,703898956
PBK	1,251373622	1,701420269
MCM6	1,303084192	1,694172193
GINS2	1,510611427	1,687254971
NASP	1,66945096	1,683201303
RRM2	1,721672665	1,662657458
CHAF1A	1,473689647	1,643965336
USP1	1,404084123	1,642606243
RNF168	1,37186688	1,628617206
NUSAP1	1,554603254	1,622824438
PRR11	1,348098887	1,604780366
CSE1L	1,490909407	1,600682611
PDCD11	1,428519593	1,595394439
NCAPH	1,294345584	1,588163322
IQCB1	1,22448104	1,583545017
NCAPD3	1,535563603	1,567952932
CTHRC1	1,498513087	1,556715265
APEX1	1,588553054	1,54079781
TOP2B	1,718954705	1,535247074
B9D1	1,042560637	1,533772027
RBM12B	1,040780775	1,531252867
ZWINT	1,407791174	1,528612024
NDC80	1,588936373	1,526717804
ASF1B	1,101933458	1,524324356
MDN1	1,359188809	1,499898511
PCNA	1,449532342	1,487801304

CDC20	1,588994418	1,485366743
RMI1	1,633736554	1,469359551
ZNF507	1,270105108	1,461192975
EZH2	1,212622337	1,456294924
DYNC1H1	1,794780477	1,45570769
TRIP13	1,575981926	1,452702682
POLA2	1,427674797	1,448620026
EED	1,335952851	1,444943132
CDC25A	1,232485998	1,435353769
HMGB3	1,120089002	1,43263268
TRMT11	1,610579804	1,422105307
COL12A1	1,23276962	1,422002676
PLOD1	1,247398935	1,417171654
CCDC112	1,04988406	1,411357176
LONP1	1,620066611	1,403597261
PRPF40A	1,361970397	1,402580444
SLBP	1,668452238	1,401156031
KIF2C	1,471599364	1,38214715
MCM7	1,426809354	1,376233619
MBD3	1,224976243	1,373415244
SASS6	1,10894975	1,364701689
NUCKS1	1,080829056	1,361647494
BCLAF1	1,470095658	1,357278767
ATAD2	1,479878554	1,356842314
HIST1H2AG	1,259899416	1,324345662
BUB1	1,129499582	1,309715306
PITRM1	1,090096091	1,299297319
TSN	1,203853085	1,291780273
NUP43	1,749672567	1,288698529
BAZ1B	1,462529754	1,287644852
IARS	1,493511565	1,272316047
NEIL3	1,296933523	1,269415689
KPNA2	1,187098237	1,267391697
CBX3	1,416455342	1,252487497
RPA2	1,467321078	1,250810459
ANKRD26	1,152019697	1,25017001
SKP2	1,47290261	1,247808328
POLR3C	1,492365526	1,239026958
CTPS	1,47203687	1,235372152
NCAPG	1,435187808	1,223220035
CDC7	1,659685804	1,220980925
SART3	1,41567608	1,214817793
NUP133	1,106193802	1,214372412
ACP6	1,405503778	1,213841863
PDS5A	1,396402401	1,210468233
DNAJC9	1,404903258	1,209918354
BUB1B	1,231534918	1,207481295

INCENP	1,516049392	1,201788168
PSMD8	1,226514128	1,19363282
TRIM28	1,196686185	1,189473793
SMC4	1,18582971	1,170658111
HELLS	1,269037234	1,156957424
RAD9A	1,46056916	1,147577854
PARP1	1,389916132	1,136469745
HSPA14	1,559926057	1,132243776
CHAF1B	1,395045943	1,12351374
LARP7	1,630962169	1,121571844
SMAD5	1,706437437	1,119467834
RRM1	1,72412682	1,10887192
TMEM138	1,433251602	1,10307947
MCM4	1,454125541	1,102349392
MIPEP	1,178053805	1,100664871
NIPBL	1,431063764	1,099564131
TMPO	1,190672629	1,097330309
SLTM	1,413772491	1,094038608
MTA3	1,5118393	1,089258527
DIS3	1,570052882	1,088546895
PSMD10	1,127284237	1,087950131
APPBP2	1,333530091	1,085959974
NOL9	1,648724454	1,082700549
VRK1	1,345297011	1,079032858
ZBTB8A	1,239900055	1,075126664
ETV4	1,285161818	1,067140835
RFC4	1,520152148	1,047572266
MED4	1,615917662	1,046729455
ANKRD10	1,618808576	1,040224139
RFC2	1,627676725	1,029193094
RAD51	1,368162526	1,028939706
MCM3AP	1,477451611	1,028584852
WDR77	1,488619856	1,021031959
SRRM2	1,132398273	1,019494312
LRPPRC	1,398210814	1,005411721
PCGF5	-1,271493486	-1,001303762
PNO1	-1,001035867	-1,011449177
UBE2E1	1,156582425	-1,016765167
TMUB2	-1,197159952	-1,018581813
RPL14	1,06442255	-1,033652655
COX7A2L	-1,326865172	-1,038946203
OAT	-1,186655574	-1,052620042
PGK1	-1,011204939	-1,065710778
STX5	-1,619340505	-1,077180457
XBP1	-1,115822324	-1,117953851
ITGB1BP3	-1,178040502	-1,119528767
GPD1	-1,040810741	-1,125560294

PELI2	-1,563328446	-1,128725881
PTP4A3	-1,54740307	-1,178902422
ACADL	-1,150827201	-1,193026938
FBP1	-1,359000948	-1,21063746
FAM110B	-1,260664396	-1,211746282
DNAJB5	-1,379458475	-1,21378182
GAMT	-1,272552277	-1,217602864
HSD17B8	-1,098231844	-1,218502721
RND3	-1,056552294	-1,224422548
RNF14	-1,162899882	-1,256237766
CITED2	-1,158451192	-1,259280611
FXYD1	-1,281654431	-1,26051334
BCL2L1	-1,592180197	-1,262573518
RCAN1	-1,233293642	-1,290724068
CD74	-1,07800085	-1,323454506
KEAP1	-1,396304198	-1,33075948
SEMA3F	-1,180584697	-1,359787994
PGM1	-1,343472361	-1,364050493
PRDX6	-1,261915889	-1,379846008
CDKN1B	-1,146621373	-1,387396099
SGMS1	-1,253390758	-1,405462237
EGFR	-1,233600462	-1,422693152
GYG1	-1,329368708	-1,431997309
RIOK3	-1,054898709	-1,43625904
ARHGAP18	-1,331230939	-1,465156247
CCNG1	-1,427373779	-1,495833788
PNRC1	-1,113404133	-1,578437307
SERPINF1	-1,37502794	-1,635488673
SPA17	-1,346828321	-1,668771597
PABPC4	-1,150850513	-1,672948637
GSTM4	-1,297345798	-1,679095506
WSB1	-1,183414766	-1,692795993
DNAJB1	-1,2317327	-1,710719244
MFAP5	-1,24756762	-1,714414565
ZFAND5	-1,242491225	-1,721018779
PLAC9	-1,237803655	-1,72321973
UGDH	-1,047207662	-1,726010915
TPM3	-1,256302409	-1,757641978
PPL	-1,104309198	-1,782759917
USP2	-1,426977025	-1,788861294
OXCT1	-1,225412082	-1,809633415
FCN1	-1,024830218	-1,813528054
LAMB2	-1,323908694	-1,835546153
FHL1	-1,282555714	-1,837307948
PON2	-1,246695713	-1,853805839
RHAG	-1,330402945	-1,903382974
SYT16	-1,528937678	-1,952187312

HBA2	-1,094989424	-2,000657536
SGK1	-1,24077182	-2,045396718
MYC	-1,087659943	-2,059241774
CSRP2	-1,212564077	-2,068316945
G0S2	-1,182025184	-2,07139454
PMP22	-1,238827998	-2,097494008
SOCS1	-1,072763951	-2,143696401
PYGM	-1,411676167	-2,149744439
TNNT3	-1,337531669	-2,231012559
LARP6	-1,580453161	-2,250463532
SCG2	-1,233280839	-2,271756419
MMP19	-1,324476516	-2,352029938
BHMT	-1,30093442	-2,383416653
TPPP3	-1,154838061	-2,389808746
PTPLA	-1,33004948	-2,448568291
HSPB8	-1,071767196	-2,537248098
UCMA	-1,070852439	-2,552982313
SQSTM1	-1,241272581	-2,572967918
ACTA2	-1,338806312	-2,60763373
IRF1	-1,184983287	-2,619729752
GFPT2	-1,01200468	-2,650625357
MYL4	-1,289977575	-2,68116652
PDLIM7	-1,14067578	-2,709895962
CYR61	-1,323059389	-2,728154652
FGL2	-1,462132139	-2,735059744
ARL4D	-1,088345309	-2,755944915
TPM1	-1,251366711	-2,796482174
ALDOA	-1,066482265	-2,848949291
GADD45B	-1,72506605	-2,854287579
SMTNL2	-1,419310004	-2,88689641
AGMAT	-1,170943998	-2,981235596
ARG1	-1,106117494	-3,007478461
FGFBP1	-1,183561384	-3,067809264
TFPI2	-1,138566889	-3,292959877
HBZ	-1,109821725	-3,341589226
AQP4	-1,214599306	-3,380177723
PFKM	-1,417616518	-3,5355142
DES	-1,288766034	-3,579917766
DUSP1	-1,19217523	-3,698381715
MYBPC3	-1,379809348	-3,731048672
AK1	-1,437474793	-3,762280958
EGR1	-1,07853509	-3,763053099
KBTBD5	-1,229927474	-3,777580701
CRYAB	-1,604894043	-3,933910079
HBE1	-1,171460297	-4,006521892
MYH1	-1,248258852	-4,017824114
FAAH	-1,330166301	-4,076964349

TNNI1	-1,154403428	-4,085297192
SLC25A4	-1,093958214	-4,090428108
TNNT1	-1,221512013	-4,108639043
PTGS2	-1,008457828	-4,149433074
MYL2	-1,247295011	-4,183669956
KBTBD10	-1,283872922	-4,415883333
TNNI2	-1,267088126	-4,478292988
MYL1	-1,208658053	-4,543844011
TNNC2	-1,300646577	-4,58311859
MYL3	-1,27998798	-4,590534562
SERPINE1	-1,345779118	-4,62905436
ACTC1	-1,286907341	-4,632952545
ACTA1	-1,26185373	-4,672032915
TNNC1	-1,33456375	-4,68683922
DUSP5	-1,33846471	-4,713807022
ADIPOQ	-1,271351292	-4,795042812
MYH7	-1,231788442	-4,915274638
MMP3	-1,161137698	-5,016746785
CKM	-1,198204618	-5,025329225
MYBPC2	-1,296441783	-5,098959152
MYOZ1	-1,303214295	-5,189963945
ACTN3	-1,418657167	-5,226718197
MBP	-1,531137968	-5,266839799
PVALB	-1,232827434	-5,599308655

Supplementary Table 7 : Gene ontology terms enriched by the top 91 DE genes in wound-healing vs. control comparison, commonly identified by the analyses of both technologies

GO terms enriched by the top 86 up-regulated genes

ONTOLOGY	ID	Description	pvalue	p.adjust	geneID	Count
BP	GO:0010942	positive regulation of cell death	3,3767E-08	8,1445E-05	ARHGEF2/BCL2L1/BMP2/CYR61/DDIT4/DUSP1/EGR1/G0S2/GADD45B/GADD45G/HMOX1/LGALS9/MMP3/PTGS2/RHOB/SQSTM1/THBS1/YWHAZ	18
BP	GO:0019221	cytokine-mediated signaling pathway	9,0149E-08	0,00010872	ARG1/BCL2L1/CXCR4/EGR1/HMOX1/JUNB/LMNB1/MMP1/MMP2/MMP3/PELI1/PTGS2/SOCS1/SQSTM1/TIMP1/YWHAZ	16
BP	GO:0032963	collagen metabolic process	6,2247E-07	0,00050046	ARG1/LARP6/MMP1/MMP13/MMP19/MMP2/MMP3	7
BP	GO:0007162	negative regulation of cell adhesion	9,7048E-07	0,00053711	ARG1/BMP2/DUSP1/LGALS9/PELI1/SERPINE1/SMAD7/SOCS1/THBS1/TNC	10
BP	GO:0008284	positive regulation of cell proliferation	1,3461E-06	0,00053711	ARG1/BCL2L1/BMP2/CYR61/EGR1/GLUL/HMOX1/LGALS9/MMP2/ODC1/PELI1/PTGS2/SCG2/TGM1/THBS1/TIMP1/TNC	17
BP	GO:0030334	regulation of cell migration	1,4271E-06	0,00053711	ARHGEF2/BMP2/CYR61/DUSP1/HMOX1/LGALS9/MMP3/PTGS2/RHOB/RND3/RRAS2/SEMA3F/SERPINE1/SGK1/SMAD7/THBS1/TIMP1	17
BP	GO:0001666	response to hypoxia	1,6679E-06	0,00053711	BMP2/CXCR4/CYBB/DDIT4/EGR1/HMOX1/MMP2/MMP3/PTGS2/SLC2A1/THBS1	11
BP	GO:0001944	vasculature development	3,6943E-06	0,00074256	CHD7/CYBB/CYR61/EGR1/HMOX1/JUNB/MMP19/MMP2/PTGS2/RHOB/SCG2/SERPINE1/SMAD7/THBS1/ZFAND5	15
BP	GO:0022617	extracellular matrix disassembly	4,541E-06	0,00078235	MMP1/MMP13/MMP19/MMP2/MMP3/TIMP1	6
BP	GO:0001817	regulation of cytokine production	7,7742E-06	0,00114584	ARG1/ARHGEF2/CYBB/EGR1/HMOX1/LGALS9/PELI1/PTGS2/SERPINE1/SMAD7/SOCS1/THBS1/UBE2J1	13
BP	GO:0035239	tube morphogenesis	8,076E-06	0,00114584	BMP2/CHD7/CYBB/CYR61/HMOX1/JUNB/MMP19/MMP2/PTGS2/RHOB/SCG2/SERPINE1/SKI/SMAD7/THBS1/TNC	16
BP	GO:0038066	p38MAPK cascade	1,2566E-05	0,00151544	BMP2/DUSP1/GADD45B/GADD45G/LGALS9	5
BP	GO:0001101	response to acid chemical	1,5653E-05	0,00165388	ARG1/BCL2L1/CYBB/DGAT2/DUSP1/EGR1/MMP2/PTGS2/SOCS1/TNC	10
BP	GO:0002704	negative regulation of leukocyte mediated immunity	1,5771E-05	0,00165388	ARG1/HMOX1/LGALS9/SMAD7	4

BP	GO:0040017	positive regulation of locomotion	1,7838E-05	0,00179268	ARHGEF2/BMP2/CYR61/HMOX1/LGALS9/PTGS2/RHOB/RRAS2/SCG2/SEMA3F/SERPINE1/THBS1	12
BP	GO:0009636	response to toxic substance	2,2537E-05	0,00217439	ACTB/ARG1/BCL2L1/CYBB/DUSP1/EGR1/HMOX1/MPO/PTGS2/RHOB/SLC30A1/TNC	12
BP	GO:0001816	cytokine production	2,3604E-05	0,00218973	ARG1/ARHGEF2/CYBB/EGR1/HMOX1/LGALS9/PELI1/PTGS2/SERPINE1/SMAD7/SOCS1/THBS1/UBE2J1	13
BP	GO:0007565	female pregnancy	2,6606E-05	0,00237679	ARG1/JUNB/LGALS9/MMP2/PTGS2/SLC2A1/TIMP1	7
BP	GO:0051707	response to other organism	3,1624E-05	0,00252617	ARG1/BCL2L1/BMP2/CHD7/CHIT1/CXCR4/DDIT4/JUNB/LGALS9/MPO/ODC1/PELI1/PTGS2/SERPINE1	14
BP	GO:0043207	response to external biotic stimulus	3,2467E-05	0,00252617	ARG1/BCL2L1/BMP2/CHD7/CHIT1/CXCR4/DDIT4/JUNB/LGALS9/MPO/ODC1/PELI1/PTGS2/SERPINE1	14
BP	GO:2001237	negative regulation of extrinsic apoptotic signaling pathway	3,6342E-05	0,00265379	ARHGEF2/BCL2L1/HMOX1/SCG2/SERPINE1/THBS1	6
BP	GO:0042035	regulation of cytokine biosynthetic process	3,7408E-05	0,00265379	CYBB/EGR1/HMOX1/THBS1/UBE2J1	5
BP	GO:1902041	regulation of extrinsic apoptotic signaling pathway via death domain receptors	3,7408E-05	0,00265379	ARHGEF2/BCL2L1/HMOX1/SERPINE1/THBS1	5
BP	GO:0035690	cellular response to drug	4,1777E-05	0,00281946	ACTB/ARG1/ARHGEF2/CYBB/DDIT4/EGR1/HMOX1/MMP3/PTGS2/RHOB	10
BP	GO:0006954	inflammatory response	5,0774E-05	0,00310109	BMP2/CXCR4/CYBB/HMOX1/LGALS9/MMP3/PTGS2/SCG2/SERPINE1/THBS1/TIMP1/ZYX	12
BP	GO:0002695	negative regulation of leukocyte activation	5,9276E-05	0,00340411	ARG1/HMOX1/LGALS9/PELI1/SMAD7/SOCS1	6
BP	GO:0042089	cytokine biosynthetic process	6,5955E-05	0,00357144	CYBB/EGR1/HMOX1/THBS1/UBE2J1	5
BP	GO:0042107	cytokine metabolic process	6,5955E-05	0,00357144	CYBB/EGR1/HMOX1/THBS1/UBE2J1	5
BP	GO:0050900	leukocyte migration	6,8112E-05	0,00357144	CXCR4/DUSP1/HMOX1/LGALS9/MMP1/SCG2/SERPINE1/SLC16A1/THBS1	9
BP	GO:0050868	negative regulation of T cell activation	7,3266E-05	0,00368423	ARG1/LGALS9/PELI1/SMAD7/SOCS1	5
BP	GO:0050920	regulation of chemotaxis	7,3318E-05	0,00368423	CXCR4/DUSP1/LGALS9/SCG2/SEMA3F/SERPINE1/THBS1	7
BP	GO:0044706	multi-multicellular organism process	7,7665E-05	0,00382304	ARG1/JUNB/LGALS9/MMP2/PTGS2/SLC2A1/TIMP1	7
BP	GO:0097237	cellular response to toxic substance	8,104E-05	0,00390938	ACTB/ARG1/CYBB/EGR1/HMOX1/MPO/PTGS2/RHOB	8

BP	GO:0071229	cellular response to acid chemical	9,1989E-05	0,00426687	BCL2L1/CYBB/DGAT2/EGR1/MMP2/SOCS1/TNC	7
BP	GO:1901654	response to ketone	9,1989E-05	0,00426687	ARG1/BCL2L1/CYBB/DDIT4/DUSP1/THBS1/TNC	7
BP	GO:0001701	in utero embryonic development	0,00016927	0,00605743	BCL2L1/BMP2/CDKN1C/CHD7/CYR61/JUNB/KEAP1/SLC30A1/ZFAND5	9
BP	GO:1904019	epithelial cell apoptotic process	0,00017077	0,00605743	BCL2L1/HMOX1/SCG2/SERPINE1/THBS1	5
BP	GO:0008285	negative regulation of cell proliferation	0,00035371	0,01079757	ARG1/BMP2/CDKN1C/DUSP1/HMOX1/LGALS9/MXI1/PELI1/PTGS2/SCG2/SKI/THBS1	12
BP	GO:0010035	response to inorganic substance	0,00037527	0,01079757	ARG1/CYBB/DUSP1/HMOX1/JUNB/MMP3/MPO/PTGS2/RHOB/SLC30A1/THBS1	11
BP	GO:0060324	face development	0,00037932	0,01079757	CHD7/MMP2/SKI/ZFAND5	4
BP	GO:0010565	regulation of cellular ketone metabolic process	0,00045674	0,01251887	BMP2/DGAT2/EGR1/ODC1/PTGS2	5
BP	GO:0040013	negative regulation of locomotion	0,00046911	0,01271327	DUSP1/HMOX1/RHOB/SEMA3F/SERPINE1/SMAD7/THBS1/TIMP1	8
BP	GO:0007178	transmembrane receptor protein serine/threonine kinase signaling pathway	0,00048538	0,01281996	BMP2/CDKN1C/CYR61/EGR1/SKI/SMAD7/THBS1/ZYX	8
BP	GO:1904035	regulation of epithelial cell apoptotic process	0,00051378	0,01318339	HMOX1/SCG2/SERPINE1/THBS1	4
BP	GO:0001934	positive regulation of protein phosphorylation	0,00055282	0,01388967	ARHGEF2/BMP2/CXCR4/CYR61/DUSP5/EGR1/GADD45B/GADD45G/LGALS9/PTGS2/SOCS1/SQSTM1/THBS1	13
BP	GO:0033687	osteoblast proliferation	0,00069752	0,01617702	BMP2/CYR61/JUNB	3
BP	GO:0043200	response to amino acid	0,0007133	0,01631468	ARG1/BCL2L1/CYBB/MMP2/SOCS1	5
BP	GO:0043086	negative regulation of catalytic activity	0,00071698	0,01631468	CDKN1C/DUSP1/DUSP5/GADD45B/PPP1R14B/PTGS2/SERPINE1/SMAD7/SOCS1/TFPI2/THBS1/TIMP1	12
BP	GO:0030509	BMP signaling pathway	0,00080324	0,01793897	BMP2/CYR61/EGR1/SKI/SMAD7	5
BP	GO:0071230	cellular response to amino acid stimulus	0,00087901	0,01927431	BCL2L1/CYBB/MMP2/SOCS1	4
BP	GO:0032101	regulation of response to external stimulus	0,00089597	0,01929951	ARG1/CXCR4/DUSP1/LGALS9/MMP3/PTGS2/SCG2/SEMA3F/SERPINE1/THBS1/ZYX	11
BP	GO:0001935	endothelial cell proliferation	0,00090139	0,01929951	ARG1/BMP2/HMOX1/SCG2/THBS1	5
BP	GO:0072593	reactive oxygen species metabolic process	0,00098332	0,02044628	CYBB/CYR61/DDIT4/MMP3/MPO/PTGS2/THBS1	7
BP	GO:0003006	developmental process involved in reproduction	0,00099811	0,02057647	BASP1/BCL2L1/CDKN1C/CHD7/CYR61/JUNB/MMP19/PTGS2/TNC/UBE2J1	10
BP	GO:0031670	cellular response to nutrient	0,00103331	0,02094401	CYBB/HMOX1/PTGS2/TNC	4

BP	GO:0071772	response to BMP	0,00118573	0,02302033	BMP2/CYR61/EGR1/SKI/SMAD7	5
BP	GO:0071773	cellular response to BMP stimulus	0,00118573	0,02302033	BMP2/CYR61/EGR1/SKI/SMAD7	5
BP	GO:0001649	osteoblast differentiation	0,00125356	0,02308081	BMP2/CYR61/JUNB/RRAS2/SKI/TNC	6
BP	GO:0002886	regulation of myeloid leukocyte mediated immunity	0,00158967	0,02700205	ARG1/HMOX1/LGALS9	3
BP	GO:0034405	response to fluid shear stress	0,00158967	0,02700205	PTGS2/SMAD7/TFPI2	3
BP	GO:0031960	response to corticosteroid	0,00168903	0,0280962	ARG1/CYBB/DDIT4/DUSP1/PTGS2	5
BP	GO:0009611	response to wounding	0,00172999	0,02858044	ACTB/ARG1/CYR61/HMOX1/SERPINE1/TFPI2/THBS1/TIMP1/TNC/YWHAZ	10
BP	GO:0090049	regulation of cell migration involved in sprouting angiogenesis	0,00178491	0,02908915	HMOX1/PTGS2/THBS1	3
BP	GO:0031667	response to nutrient levels	0,00189254	0,03023043	ARG1/CYBB/GLUL/HMOX1/MPO/PTGS2/SLC16A1/SLC2A1/TNC	9
BP	GO:0071470	cellular response to osmotic stress	0,00199449	0,03103679	ARHGEF2/PTGS2/SLC2A1	3
BP	GO:0007566	embryo implantation	0,00221878	0,03283245	MMP2/PTGS2/TIMP1	3
BP	GO:0050777	negative regulation of immune response	0,00237789	0,03455097	ARG1/HMOX1/LGALS9/SMAD7	4
BP	GO:0000302	response to reactive oxygen species	0,00271425	0,03873832	ARG1/DUSP1/HMOX1/MMP3/MPO/RHOB	6
BP	GO:0097327	response to antineoplastic agent	0,00284352	0,04010858	ARG1/DDIT4/EGR1/HMOX1	4
BP	GO:1901699	cellular response to nitrogen compound	0,00301581	0,0413303	ACTB/ARG1/ARHGEF2/BCL2L1/CYBB/EGR1/MMP2/MMP3/PTGS2/SOCS1	10
BP	GO:0046890	regulation of lipid biosynthetic process	0,00314265	0,0426292	BMP2/CYR61/DGAT2/EGR1/PTGS2	5
BP	GO:0061448	connective tissue development	0,00330319	0,04353719	BMP2/CYR61/DGAT2/EGR1/MMP13/TIMP1	6
BP	GO:0030217	T cell differentiation	0,00354459	0,0458356	CHD7/EGR1/LGALS9/SMAD7/SOCS1	5
BP	GO:0002042	cell migration involved in sprouting angiogenesis	0,00357259	0,0458356	HMOX1/PTGS2/THBS1	3
BP	GO:0030952	establishment or maintenance of cytoskeleton polarity	0,00374679	0,04658374	RHOB/RND3	2
BP	GO:0032461	positive regulation of protein oligomerization	0,00374679	0,04658374	MMP1/MMP3	2
BP	GO:0046697	decidualization	0,00374679	0,04658374	JUNB/PTGS2	2
BP	GO:0072215	regulation of metanephros development	0,00374679	0,04658374	BASP1/EGR1	2
CC	GO:0031012	extracellular matrix	5,2662E-05	0,01190166	CYR61/MMP1/MMP13/MMP19/MMP2/MMP3/SERPINE1/TFPI2/THBS1/TIMP1/TNC	11
MF	GO:0004222	metalloendopeptidase activity	0,00013381	0,0373335	MMP1/MMP13/MMP19/MMP2/MMP3	5
**GO terms enriched by the top 4 down-regulated genes**						
**ONTOLOGY**	**ID**	**Description**	**pvalue**	**p.adjust**	**geneID**	**Count**
MF	GO:0015556	carboxylate transmembrane transporter a	0,00462828	0,04516037	SLC25A12	1
MF	GO:0005326	neurotransmitter transporter activity	0,01016099	0,04516037	SLC25A12	1

Supplementary Table 8: Gene ontology terms enriched by the top 351 DE genes in regenerative vs. control comparison, commonly identified by the analyses of both technologies						
**GO terms enriched by the top 181 up-regulated genes**						
**ONTOLOGY**	**ID**	**Description**	**pvalue**	**p.adjust**	**geneID**	**Count**
BP	GO:0140014	mitotic nuclear division	6,0398E-08	8,77793E-05	AURKA/AURKB/CCNJ/CDC20/CDCA8/FLNA/KIF11/KIF22/KIF23/MAD2L1/MAD2L1BP/MTBP/NEK2/NIPBL/PLK1/PRC1/RCC1/TRIP13	18
BP	GO:0000280	nuclear division	6,5167E-08	8,77793E-05	AURKA/AURKB/CCNJ/CDC20/CDCA8/FLNA/KIF11/KIF22/KIF23/MAD2L1/MAD2L1BP/MTBP/NEK2/NIPBL/PLK1/PRC1/RCC1/RPA2/TOP2B/TRIP13	20
BP	GO:0007059	chromosome segregation	2,2754E-07	0,000149307	AURKB/CDC20/CDCA8/FEN1/KIF22/KIF23/MAD2L1/NEK2/NIPBL/PLK1/PRC1/RCC1/SFPQ/TOP1/TOP2B/TRIP13/UBE2I	17
BP	GO:0000819	sister chromatid segregation	2,9059E-07	0,000149307	AURKB/CDC20/CDCA8/FEN1/KIF22/KIF23/MAD2L1/NEK2/NIPBL/PLK1/PRC1/SFPQ/TOP2B/TRIP13	14
BP	GO:0007052	mitotic spindle organization	3,6409E-07	0,000149307	AURKA/AURKB/CDC20/FLNA/KIF11/KIF23/NEK2/PLK1/PRC1/RCC1/STMN1	11
BP	GO:0008380	RNA splicing	3,8796E-07	0,000149307	CLK3/CLNS1A/CSTF1/DHX15/FUS/HNRNPM/MPHOSPH10/PRPF3/PRPF39/PTBP1/RBMX/RBMXL1/SART3/SFPQ/SNRNP40/SNRPA/SNRPA1/TARDBP/TRA2B/ZNF326/ZRANB2	21
BP	GO:0007088	regulation of mitotic nuclear division	8,1773E-07	0,000275371	AURKA/AURKB/CCNJ/CDC20/KIF11/MAD2L1/MAD2L1BP/MTBP/NEK2/NIPBL/PLK1/RCC1/TRIP13	13

BP	GO:0006325	chromatin organization	2,2244E-06	0,00043043	ANP32E/ASF1B/AURKA/AURKB/BAZ1B/BRD3/CBX3/CHD7/FBL/HCFC1/HELLS/HIST3H2BB/HMGB2/LOXL2/MBD3/NASP/NIPBL/PBRM1/RBM14/SAFB/SART3/SFPQ/SMARCC1/TCF7L1/TOP1	25
BP	GO:0010564	regulation of cell cycle process	9,5211E-06	0,001508813	APEX1/AURKA/AURKB/CBX3/CCNJ/CDC20/FEN1/GPNMB/HAUS1/KIF11/KIF23/MAD2L1/MAD2L1BP/MTBP/NEK2/NIPBL/PLK1/PRC1/RBM14/RCC1/RPA2/RRM2/SFPQ/TRIP13	24
BP	GO:0030198	extracellular matrix organization	1,9083E-05	0,002570432	COL12A1/FN1/HAS2/ITGB1/KAZALD1/LAMA1/LOXL2/MFAP2/MMP13/MMP2/PLOD1/SULF1/TGFBI/THBS1/TNC	15
BP	GO:0051983	regulation of chromosome segregation	2,0935E-05	0,002588695	AURKB/CDC20/FEN1/MAD2L1/NEK2/NIPBL/PLK1/SFPQ/TRIP13	9
BP	GO:1903311	regulation of mRNA metabolic process	5,0836E-05	0,004891144	APEX1/HNRNPM/MYEF2/PTBP1/RBMX/RBMXL1/SAFB/SART3/SLTM/SNRPA/TARDBP/TRA2B/VIM	13
BP	GO:0044772	mitotic cell cycle phase transition	0,00010414	0,008760777	APEX1/AURKA/AURKB/CDC20/GPNMB/HAUS1/ITGB1/MAD2L1/MAD2L1BP/MTBP/NASP/NEK2/PLK1/RCC1/RPA2/RPS6KB1/RRM2/TRIP13	18
BP	GO:0007094	mitotic spindle assembly checkpoint	0,00022892	0,015417687	AURKB/CDC20/MAD2L1/PLK1/TRIP13	5
BP	GO:0031577	spindle checkpoint	0,00022892	0,015417687	AURKB/CDC20/MAD2L1/PLK1/TRIP13	5
BP	GO:0071173	spindle assembly checkpoint	0,00022892	0,015417687	AURKB/CDC20/MAD2L1/PLK1/TRIP13	5
BP	GO:0071174	mitotic spindle checkpoint	0,00022892	0,015417687	AURKB/CDC20/MAD2L1/PLK1/TRIP13	5
BP	GO:1905819	negative regulation of chromosome separation	0,00037004	0,020344809	AURKB/CDC20/MAD2L1/PLK1/TRIP13	5
BP	GO:0031145	anaphase-promoting complex-dependent catabolic process	0,00042923	0,023126661	AURKA/AURKB/CDC20/MAD2L1/PLK1	5

BP	GO:0001503	ossification	0,00056362	0,027855528	BMP2/CTHRC1/FBL/GPNMB/ID3/JAG1/JUNB/KAZALD1/MMP13/MMP2/NIPBL/RBMX/TNC	13
BP	GO:0071103	DNA conformation change	0,00067542	0,031922416	ASF1B/HELLS/HIST3H2BB/HMGB2/NASP/NIPBL/RPA2/SART3/TOP1/TOP2B	10
BP	GO:0071824	protein-DNA complex subunit organization	0,00071188	0,032306827	ANP32E/ASF1B/HELLS/HIST3H2BB/HMGB2/NASP/PBRM1/RPA2/SART3/SMARCC1	10
BP	GO:0048514	blood vessel morphogenesis	0,0008756	0,037442171	CHD7/CYBB/FN1/GPNMB/HAS2/HMOX1/ITGB1/JAG1/JUNB/LAMA1/LOXL2/MMP2/SMAD7/SULF1/TGFBI/THBS1	16
BP	GO:0001649	osteoblast differentiation	0,00094285	0,038485583	BMP2/CTHRC1/FBL/GPNMB/ID3/JAG1/JUNB/RBMX/TNC	9
BP	GO:0007127	meiosis I	0,00106458	0,042240906	AURKA/PLK1/RPA2/TOP2B/TRIP13	5
BP	GO:0035024	negative regulation of Rho protein signal transduction	0,00111083	0,043272105	ITGB1/STMN1/TNFAIP1	3
BP	GO:0034502	protein localization to chromosome	0,00112437	0,043272105	AURKB/DKC1/MTBP/NIPBL/PLK1/RPA2	6
BP	GO:0035239	tube morphogenesis	0,00123685	0,046930658	BMP2/CHD7/CTHRC1/CYBB/FN1/GPNMB/HAS2/HMOX1/ITGB1/JAG1/JUNB/LAMA1/LOXL2/MMP2/NIPBL/SMAD7/SULF1/TGFBI/THBS1/TNC	20
BP	GO:0045787	positive regulation of cell cycle	0,00132296	0,049500732	APEX1/AURKA/AURKB/CCNJ/FEN1/HCFC1/KIF23/MAD2L1/MTBP/NIPBL/PGGT1B/RPS6KB1/SFPQ	13

CC	GO:0000785	chromatin	1,2256E-08	4,03217E-06	ANP32E/ASF1B/BAZ1B/CBX3/HELLS/HIST3H2BB/HMGB2/JUNB/KIF22/LOXL2/MBD3/MTBP/NASP/NIPBL/PBRM1/PLK1/RBMX/RCC1/RPA2/SFPQ/SMARCC1/TARDBP/TMPO/TOP2B	24
CC	GO:0044452	nucleolar part	3,6423E-07	3,40189E-05	COIL/DKC1/FBL/IPO11/MPHOSPH10/NOL11/NOLC1/POLR1C/PSPC1/PWP2/RRP9/SMAD7/TOP1/UBE2I	14
CC	GO:0000228	nuclear chromosome	4,0004E-07	3,40189E-05	ANP32E/APEX1/ASF1B/AURKA/AURKB/BAZ1B/CBX3/FEN1/HMGB2/JUNB/MBD3/NASP/NEK2/NIPBL/PBRM1/PLK1/RBMX/RCC1/RPA2/SMARCC1/TARDBP/TOP1/UBE2I	23
CC	GO:0044454	nuclear chromosome part	4,136E-07	3,40189E-05	ANP32E/APEX1/ASF1B/AURKA/AURKB/BAZ1B/CBX3/FEN1/HMGB2/JUNB/MBD3/NASP/NIPBL/PBRM1/PLK1/RBMX/RCC1/RPA2/SMARCC1/TARDBP/TOP1/UBE2I	22
CC	GO:0005732	small nucleolar ribonucleoprotein complex	1,0598E-06	6,97368E-05	DKC1/FBL/MPHOSPH10/NOLC1/RRP9/SNRNP40	6
CC	GO:0000775	chromosome, centromeric region	2,0765E-06	0,000113859	AURKA/AURKB/BAZ1B/CBX3/CDCA8/HELLS/KIF22/MAD2L1/MTBP/NEK2/NUP160/NUP85/PLK1	13
CC	GO:0098687	chromosomal region	6,8741E-06	0,000299166	APEX1/AURKA/AURKB/BAZ1B/CBX3/CDCA8/FEN1/HELLS/KIF22/MAD2L1/MTBP/NEK2/NUP160/NUP85/PLK1/RPA2	16
CC	GO:0005819	spindle	7,2745E-06	0,000299166	AURKA/AURKB/CBX3/CDC20/CDCA8/HAUS1/IQCB1/KIF11/KIF22/KIF23/MAD2L1/MAD2L1BP/NEK2/NUP85/PLK1/PRC1	16

CC	GO:0000793	condensed chromosome	8,7524E-06	0,000319949	AURKA/AURKB/BAZ1B/CBX3/HMGB2/MAD2L1/NEK2/NUP85/PLK1/RCC1/RPA2/UBE2I	12
CC	GO:0000794	condensed nuclear chromosome	3,3933E-05	0,001116391	AURKA/AURKB/NEK2/PLK1/RCC1/RPA2/UBE2I	7
CC	GO:0005681	spliceosomal complex	7,5907E-05	0,00202787	DHX15/HNRNPM/MYEF2/PRPF3/PRPF39/RBMX/RBMXL1/SNRNP40/SNRPA/SNRPA1/TRA2B	11
CC	GO:0005635	nuclear envelope	7,605E-05	0,00202787	BICD2/CBX3/CSE1L/CYB/IPO11/MAD2L1/MAD2L1BP/MATR3/MCM3AP/NUP160/NUP85/RANBP1/RCC1/RRM1/TMPO/TRA2B/UBE2I	17
CC	GO:0001650	fibrillar center	8,0129E-05	0,00202787	COIL/DKC1/FBL/IPO11/NOLC1/PSPC1/SMAD7/TOP1/UBE2I	9
CC	GO:0015030	Cajal body	0,00014166	0,00332905	COIL/DKC1/FBL/NOLC1/PRPF3/SART3	6
CC	GO:0000922	spindle pole	0,00017545	0,003848233	AURKA/AURKB/CDC20/HAUS1/KIF11/MAD2L1/NEK2/PLK1/PRC1	9
CC	GO:0120114	Sm-like protein family complex	0,00019984	0,004076949	CLNS1A/PRPF3/PRPF39/SART3/SNRNP40/SNRPA/SNRPA1	7
CC	GO:0062023	collagen-containing extracellular matrix	0,00021066	0,004076949	COL12A1/CTHRC1/CTSC/F13A1/FN1/KAZALD1/LAMA1/LOXL2/MFAP2/MMP2/TGFBI/THBS1/TNC	13
CC	GO:0016604	nuclear body	0,00024572	0,004491229	APEX1/BASP1/BAZ1B/CLK3/COIL/DHX15/DKC1/FBL/HNRNPM/KIF22/NOLC1/PRPF3/PSPC1/RBM14/RPA2/SART3/SFPQ/SLTM/SNRNP40/SNRPA1/TARDBP/UBE2I	22
CC	GO:0072686	mitotic spindle	0,00026795	0,004639683	AURKA/AURKB/IQCB1/KIF11/KIF22/KIF23/MAD2L1	7

CC	GO:0000790	nuclear chromatin	0,00031763	0,00522507	ANP32E/ASF1B/CBX3/HMGB2/JUNB/MBD3/NASP/NIPBL/PBRM1/RBMX/RCC1/SMARCC1/TARDBP	13
CC	GO:0097525	spliceosomal snRNP complex	0,00033766	0,005290048	PRPF3/PRPF39/SART3/SNRNP40/SNRPA/SNRPA1	6
CC	GO:0031012	extracellular matrix	0,00037665	0,00563263	COL12A1/CTHRC1/CTSC/F13A1/FN1/HNRNPM/KAZALD1/LAMA1/LOXL2/MFAP2/MMP13/MMP2/TGFBI/THBS1/TNC	15
CC	GO:0000776	kinetochore	0,00042032	0,006012358	AURKB/KIF22/MAD2L1/MTBP/NEK2/NUP160/NUP85/PLK1	8
CC	GO:0030532	small nuclear ribonucleoprotein complex	0,00057583	0,007893646	PRPF3/PRPF39/SART3/SNRNP40/SNRPA/SNRPA1	6
CC	GO:0000779	condensed chromosome, centromeric region	0,00063974	0,008419001	AURKA/AURKB/CBX3/MAD2L1/NEK2/NUP85/PLK1	7
CC	GO:0005876	spindle microtubule	0,00067586	0,008552287	AURKA/AURKB/KIF11/PLK1/PRC1	5
CC	GO:0005643	nuclear pore	0,00142563	0,017371596	BICD2/MAD2L1/MCM3AP/NUP160/NUP85/RANBP1	6
CC	GO:0051233	spindle midzone	0,00192891	0,022664676	AURKA/AURKB/CDCA8/PLK1	4
CC	GO:0032040	small-subunit processome	0,00220415	0,025005709	FBL/MPHOSPH10/PWP2/RRP9	4
CC	GO:0016607	nuclear speck	0,00235333	0,02580816	APEX1/BASP1/CLK3/DHX15/KIF22/PRPF3/PSPC1/RBM14/SART3/SFPQ/SNRNP40/SNRPA1/TARDBP	13
CC	GO:0000780	condensed nuclear chromosome, centromeric region	0,00273027	0,02807062	AURKA/AURKB/PLK1	3
CC	GO:0005721	pericentric heterochromatin	0,00273027	0,02807062	BAZ1B/CBX3/HELLS	3
CC	GO:0005875	microtubule associated complex	0,00417409	0,041614427	AURKA/AURKB/CDCA8/HAUS1/KIF11/KIF22/KIF23	7
CC	GO:0016363	nuclear matrix	0,00443417	0,042907096	HNRNPM/MATR3/PSPC1/SFPQ/VIM/ZNF326	6
CC	GO:0044420	extracellular matrix component	0,00492183	0,046265235	COL12A1/LAMA1/MFAP2/TNC	4
**GO terms enriched by the top 166 down-regulated genes**						

ONTOLOGY	ID	Description	pvalue	p.adjust	geneID	Count
BP	GO:0030049	muscle filament sliding	6,0077E-29	7,74098E-26	ACTA1/ACTC1/ACTN3/DES/MYBPC2/MYBPC3/MYH2/MYH4/MYH7/MYL1/MYL2/MYL3/MYL4/TNNC1/TNNC2/TNNI1/TNNI2/TNNT1/TNNT3/TPM1/TPM3	21
BP	GO:0033275	actin-myosin filament sliding	6,0077E-29	7,74098E-26	ACTA1/ACTC1/ACTN3/DES/MYBPC2/MYBPC3/MYH2/MYH4/MYH7/MYL1/MYL2/MYL3/MYL4/TNNC1/TNNC2/TNNI1/TNNI2/TNNT1/TNNT3/TPM1/TPM3	21
BP	GO:0006936	muscle contraction	1,14E-25	9,80398E-23	ABAT/ACTA1/ACTA2/ACTC1/ACTN3/ANXA6/ASPH/BIN1/CRYAB/DES/FXYD1/GAMT/MYBPC2/MYBPC3/MYH1/MYH13/MYH2/MYH4/MYH7/MYL1/MYL2/MYL3/MYL4/MYLPF/SNTA1/SNTB1/SORBS1/TNNC1/TNNC2/TNNI1/TNNI2/TNNT1/TNNT3/TPM1/TPM3	35
BP	GO:0006091	generation of precursor metabolites and energy	7,8789E-13	2,55563E-10	ACAT1/ACTN3/ADH4/ADIPOQ/ALDH1A1/ASPH/BNIP3/COX17/COX7A2L/COX7B/GAPDH/GLDC/GPD1/GPI/GYG1/MDH2/OXCT1/PARK7/PFKM/PGK1/PGM1/PRKAG2/PYGM/SLC25A12/SLC25A4/SORBS1/TPI1/XDH	28
BP	GO:0060047	heart contraction	7,9337E-13	2,55563E-10	ACTC1/ASPH/BIN1/DES/EPAS1/FXYD1/MYBPC3/MYH7/MYL1/MYL2/MYL3/MYL4/PEBP1/SNTA1/TNNC1/TNNI2/TNNT1/TNNT3/TPM1	19

BP	GO:0006937	regulation of muscle contraction	2,0214E-11	4,73562E-09	ABAT/ACTN3/ANXA6/BIN1/MYBPC3/MYH7/MYL2/MYL3/TNNC1/TNNC2/TNNI1/TNNI2/TNNT1/TNNT3/TPM1	15
BP	GO:0016053	organic acid biosynthetic process	2,2731E-10	3,44568E-08	ABAT/ACADL/ACTN3/ADIPOQ/BHMT/CD74/CSPG5/GAMT/GAPDH/GATM/GOT1/GOT2/GPD1/GPI/GSTM4/OAT/PARK7/PFKM/PGK1/PGM1/PRKAG2/SLC25A12/TPI1	23
BP	GO:0046394	carboxylic acid biosynthetic process	2,2731E-10	3,44568E-08	ABAT/ACADL/ACTN3/ADIPOQ/BHMT/CD74/CSPG5/GAMT/GAPDH/GATM/GOT1/GOT2/GPD1/GPI/GSTM4/OAT/PARK7/PFKM/PGK1/PGM1/PRKAG2/SLC25A12/TPI1	23
BP	GO:0006006	glucose metabolic process	3,0181E-10	4,32091E-08	ACTN3/ADIPOQ/CPT1A/GAPDH/GOT1/GOT2/GPD1/GPI/MDH2/PFKM/PGK1/PGM1/RBP4/SLC25A12/SORBS1/TPI1	16
BP	GO:0006094	gluconeogenesis	5,5543E-10	7,50037E-08	ADIPOQ/GAPDH/GOT1/GOT2/GPD1/GPI/MDH2/PGK1/PGM1/RBP4/SLC25A12/TPI1	12
BP	GO:0044282	small molecule catabolic process	5,821E-10	7,50037E-08	ABAT/ACADL/ACAT1/ACTN3/ADH4/ADIPOQ/ALDH1A1/CPT1A/ECHDC2/FAAH/GAPDH/GLDC/GOT1/GOT2/GPI/HEXB/OAT/OXCT1/PFKM/PGK1/PGM1/SLC25A12/TPI1	23
BP	GO:0006734	NADH metabolic process	2,46E-09	2,5358E-07	ACTN3/GAPDH/GPD1/GPI/MDH2/PFKM/PGK1/SLC25A12/TPI1	9
BP	GO:0046031	ADP metabolic process	7,9084E-09	7,54814E-07	ACTN3/AK1/GAPDH/GPD1/GPI/PFKM/PGK1/PGM1/PRKAG2/SLC25A12/TPI1	11
BP	GO:0030239	myofibril assembly	1,7206E-08	1,47799E-06	ACTA1/ACTC1/MYBPC2/MYBPC3/MYH10/MYL2/MYOZ1/TNNT1/TNNT3/TPM1	10

BP	GO:0061061	muscle structure development	1,9725E-08	1,63973E-06	ACTA1/ACTC1/ACTN3/BIN1/CRYAB/EPAS1/FHL1/MYBPC2/MYBPC3/MYH10/MYH7/MYL2/MYL3/MYLPF/MYOZ1/PGK1/RBP4/SMYD1/TNNC1/TNNI1/TNNT1/TNNT3/TPM1/UNC45B	24
BP	GO:0019320	hexose catabolic process	2,1278E-08	1,71358E-06	ACTN3/ALDH1A1/GAPDH/GPI/PFKM/PGK1/PGM1/SLC25A12/TPI1	9
BP	GO:0016052	carbohydrate catabolic process	3,0873E-08	2,41091E-06	ACTN3/ALDH1A1/GAPDH/GPD1/GPI/HEXB/PFKM/PGK1/PGM1/PRKAG2/PYGM/SLC25A12/TPI1	13
BP	GO:0010927	cellular component assembly involved in morphogenesis	6,2684E-08	3,75668E-06	ACTA1/ACTC1/MYBPC2/MYBPC3/MYH10/MYL2/MYOZ1/PMP22/TNNT1/TNNT3/TPM1	11
BP	GO:0044283	small molecule biosynthetic process	7,5188E-08	4,40361E-06	ABAT/ACADL/ACAT1/ACTN3/ADIPOQ/BHMT/CD74/CSPG5/GAMT/GAPDH/GATM/GOT1/GOT2/GPD1/GPI/GSTM4/MDH2/OAT/PARK7/PFKM/PGK1/PGM1/PRKAG2/RBP4/SLC25A12/TPI1/XDH	27
BP	GO:0055008	cardiac muscle tissue morphogenesis	9,5506E-08	5,27973E-06	ACTC1/MYBPC2/MYBPC3/MYH7/MYL2/MYL3/TNNC1/TNNI1/TPM1	9
BP	GO:0006754	ATP biosynthetic process	2,8659E-07	1,25178E-05	ACTN3/GAPDH/GPD1/GPI/PFKM/PGK1/PGM1/PRKAG2/SLC25A12/TPI1	10
BP	GO:0043467	regulation of generation of precursor metabolites and energy	1,3063E-06	4,20786E-05	ACTN3/BNIP3/COX17/COX7A2L/GPD1/PARK7/PRKAG2/SLC25A12/SORBS1	9
BP	GO:0072521	purine-containing compound metabolic process	1,5208E-06	4,77944E-05	ACAT1/ACTN3/ADSSL1/AK1/COX7A2L/GAMT/GAPDH/GMPR/GPD1/GPI/MYH4/MYH7/PARK7/PFKM/PGK1/PGM1/PRKAG2/SLC25A12/SLC2A9/TPI1/XDH	21
BP	GO:0055001	muscle cell development	2,0313E-06	5,88177E-05	ACTA1/ACTC1/BIN1/MYBPC2/MYBPC3/MYH10/MYL2/MYOZ1/TNNT1/TNNT3/TPM1	11

BP	GO:0050879	multicellular organismal movement	2,9426E-06	7,98214E-05	ACTN3/MYH7/TNNC1/TNNC2/TNNI2/TNNT1/TNNT3	7
BP	GO:0050881	musculoskeletal movement	2,9426E-06	7,98214E-05	ACTN3/MYH7/TNNC1/TNNC2/TNNI2/TNNT1/TNNT3	7
BP	GO:0097435	supramolecular fiber organization	3,2149E-06	8,62995E-05	ACTA1/ACTC1/ARHGAP18/BIN1/COL1A1/CRYAB/DES/DPT/FBLN5/MFAP5/MYBPC2/MYBPC3/MYH10/MYL2/MYOZ1/PARK7/SORBS1/TNNT1/TNNT3/TPM1/TPM3/TPPP3	22
BP	GO:0014706	striated muscle tissue development	3,5583E-06	9,35685E-05	ACTA1/ACTC1/ACTN3/MYBPC2/MYBPC3/MYH10/MYH7/MYL2/MYL3/MYLPF/RBP4/SMYD1/TNNC1/TNNI1/TPM1	15
BP	GO:0046434	organophosphate catabolic process	4,6237E-06	0,000116818	ACAT1/ACTN3/GAPDH/GPD1/GPI/PFKM/PGK1/PGM1/PRKAG2/SLC25A12/TPI1/XDH	12
BP	GO:0009636	response to toxic substance	5,1625E-06	0,000125298	ABAT/ACTC1/ADIPOQ/BNIP3/CCL5/COL1A1/CRYAB/FBLN5/GOT2/INMT/MAP1LC3A/MBP/OXCT1/PARK7/PEBP1/PON2/RBP4/SLC7A11	18
BP	GO:0043462	regulation of ATPase activity	6,7875E-06	0,000160471	ATP1B2/GABARAPL2/MYBPC3/MYL3/MYL4/TNNC1/TNNT3/TPM1	8
BP	GO:0046390	ribose phosphate biosynthetic process	7,2546E-06	0,000166921	ACAT1/ACTN3/ADSSL1/AK1/GAPDH/GPD1/GPI/PFKM/PGK1/PGM1/PRKAG2/SLC25A12/TPI1	13
BP	GO:0062013	positive regulation of small molecule metabolic process	1,6113E-05	0,000346023	ACTN3/ADIPOQ/CPT1A/GPD1/PARK7/PPARG/SLC25A12/SORBS1	8
BP	GO:0031032	actomyosin structure organization	2,3254E-05	0,000483273	ACTA1/ACTC1/MYBPC2/MYBPC3/MYH10/MYL2/MYOZ1/SORBS1/TNNT1/TNNT3/TPM1	11
BP	GO:0033238	regulation of cellular amine metabolic process	3,2152E-05	0,000622984	ABAT/BHMT/PARK7/PEBP1/SLC7A11	5

BP	GO:0005977	glycogen metabolic process	4,5781E-05	0,000854902	GYG1/PFKM/PGM1/PRKAG2/PYGM/SORBS1	6
BP	GO:0006073	cellular glucan metabolic process	4,5781E-05	0,000854902	GYG1/PFKM/PGM1/PRKAG2/PYGM/SORBS1	6
BP	GO:0062012	regulation of small molecule metabolic process	5,7366E-05	0,001051239	ACADL/ACTN3/ADIPOQ/BHMT/COX7A2L/CPT1A/GPD1/PARK7/PPARG/PRKAG2/SLC25A12/SLC7A11/SORBS1	13
BP	GO:0015980	energy derivation by oxidation of organic compounds	6,8537E-05	0,001226523	ACTN3/BNIP3/GPD1/GYG1/MDH2/PARK7/PFKM/PGM1/PRKAG2/PYGM/SLC25A12/SORBS1	12
BP	GO:0014823	response to activity	8,2108E-05	0,001410609	ADIPOQ/ADSSL1/COL4A2/GOT2/OXCT1/PEBP1	6
BP	GO:0032781	positive regulation of ATPase activity	0,00010735	0,0017847	ATP1B2/GABARAPL2/MYBPC3/MYL3/MYL4/TPM1	6
BP	GO:0052548	regulation of endopeptidase activity	0,00011297	0,001866194	ASPH/BIN1/CRYAB/GAPDH/GPI/MBP/PARK7/PEBP1/PPARG/PSMB8/SERPINF1/WFDC2/XDH	13
BP	GO:0010038	response to metal ion	0,00017697	0,002797852	ABAT/ACTA1/B2M/BNIP3/CA2/GOT1/GPI/MAP1LC3A/PARK7/PEBP1/SERPINF1/SLC25A12/TNNC1	13
BP	GO:0045471	response to ethanol	0,00021126	0,00331959	ABAT/ACTC1/ADIPOQ/GOT2/OXCT1/PEBP1/RBP4	7
BP	GO:0034764	positive regulation of transmembrane transport	0,00024907	0,003820596	ADIPOQ/ATP1B2/CA2/COX17/FXYD1/ITLN1/PARK7/SORBS1	8
BP	GO:0090087	regulation of peptide transport	0,00044907	0,006358535	ABAT/ADIPOQ/CA2/CCL5/CD74/CPT1A/GAPDH/GPI/MBP/MYH10/OXCT1/PARK7/PFKM/PKIG/RBP4/SLC25A4/SLC7A11	17
BP	GO:0015893	drug transport	0,00046223	0,006509145	ABAT/CA2/NFKBIE/PARK7/RHAG/SLC25A12/SLC25A4/SLC7A11	8

BP	GO:2000181	negative regulation of blood vessel morphogenesis	0,00060463	0,008200752	COL4A2/PGK1/PPARG/SERPINF1/SYNJ2BP/XDH	6
BP	GO:0038065	collagen-activated signaling pathway	0,00078714	0,010296765	COL1A1/COL4A1/COL4A2	3
BP	GO:1901615	organic hydroxy compound metabolic process	0,00085125	0,01107919	ABAT/ACADL/ACTN3/ADH4/ALDH1A1/EPAS1/GOT1/GPD1/PARK7/PRKAG2/RBP4/SLC25A12/SLC7A11/TPI1	14
BP	GO:0050714	positive regulation of protein secretion	0,00095551	0,012189879	ABAT/GAPDH/GPI/MBP/MYH10/OXCT1/PFKM/RBP4	8
BP	GO:1904706	negative regulation of vascular smooth muscle cell proliferation	0,00103607	0,013088005	ADIPOQ/PPARG/TPM1	3
BP	GO:0010951	negative regulation of endopeptidase activity	0,00125737	0,015578074	BIN1/CRYAB/GAPDH/GPI/PARK7/PEBP1/SERPINF1/WFDC2	8
BP	GO:0030073	insulin secretion	0,00132598	0,016011631	ABAT/CCL5/CPT1A/OXCT1/PARK7/PFKM/RBP4/SLC25A4	8
BP	GO:0055093	response to hyperoxia	0,00132964	0,016011631	BNIP3/COL1A1/PPARG	3
BP	GO:0034308	primary alcohol metabolic process	0,00144895	0,017367125	ADH4/ALDH1A1/GPD1/PARK7/RBP4	5
BP	GO:0035902	response to immobilization stress	0,00167062	0,019480468	GOT1/GPI/PPARG	3
BP	GO:0006820	anion transport	0,00187742	0,021598673	ABAT/CA2/CPT1A/FXYD1/GOT2/NFKBIE/PPARG/RHAG/SLC25A12/SLC25A4/SLC2A9/SLC7A11/VDAC1	13
BP	GO:0048871	multicellular organismal homeostasis	0,00189214	0,021671353	ABAT/ACADL/ACTN3/ADIPOQ/ALDH1A1/B2M/CA2/EPAS1/GATM/IL20RB/RBP4/RHAG	12
BP	GO:0043470	regulation of carbohydrate catabolic process	0,00197938	0,022186624	ACTN3/GPD1/PRKAG2/SLC25A12	4
BP	GO:0033500	carbohydrate homeostasis	0,00198879	0,022186624	ADIPOQ/GPI/OXCT1/PARK7/PFKM/PPARG/RBP4/SERPINF1	8
BP	GO:0042593	glucose homeostasis	0,00198879	0,022186624	ADIPOQ/GPI/OXCT1/PARK7/PFKM/PPARG/RBP4/SERPINF1	8

BP	GO:0000422	autophagy of mitochondrion	0,00207926	0,022898527	BNIP3/GABARAPL2/MAP1LC3A/PARK7/VDAC1	5
BP	GO:0061726	mitochondrion disassembly	0,00207926	0,022898527	BNIP3/GABARAPL2/MAP1LC3A/PARK7/VDAC1	5
BP	GO:0046324	regulation of glucose import	0,00223166	0,024368542	ADIPOQ/ITLN1/PRKAG2/SORBS1	4
BP	GO:0072347	response to anesthetic	0,00223166	0,024368542	ABAT/GOT2/GPI/MAP1LC3A	4
BP	GO:1904659	glucose transmembrane transport	0,00246022	0,026198327	ADIPOQ/ITLN1/PRKAG2/SLC2A9/SORBS1	5
BP	GO:0033032	regulation of myeloid cell apoptotic process	0,00250491	0,026455512	ADIPOQ/CCL5/SLC7A11	3
BP	GO:0009914	hormone transport	0,00252747	0,026476745	ABAT/ADIPOQ/CCL5/CPT1A/OXCT1/PARK7/PFKM/RBP4/SLC25A4	9
BP	GO:0043279	response to alkaloid	0,00266862	0,027817491	ABAT/GOT2/GPI/MAP1LC3A/PPARG	5
BP	GO:0010817	regulation of hormone levels	0,00267704	0,027817491	ABAT/ADH4/ADIPOQ/ALDH1A1/CCL5/CPT1A/OXCT1/PARK7/PEBP1/PFKM/RBP4/SLC25A4	12
BP	GO:0051223	regulation of protein transport	0,00274933	0,028453945	ABAT/ADIPOQ/CCL5/CPT1A/GAPDH/GPI/MBP/MYH10/OXCT1/PARK7/PFKM/PKIG/RBP4/SLC25A4/SLC7A11	15
BP	GO:0150063	visual system development	0,00287459	0,029396123	CHRDL1/COL4A1/COL8A1/CRYAB/LAMB2/MYH10/RBP4/SERPINF1/SLC7A11/UNC45B	10
BP	GO:0010939	regulation of necrotic cell death	0,00300285	0,029648801	BNIP3/HEBP2/SLC25A4	3
BP	GO:0042135	neurotransmitter catabolic process	0,00300285	0,029648801	ABAT/BHMT/GLDC	3
BP	GO:0072207	metanephric epithelium development	0,00300285	0,029648801	ACAT1/ADIPOQ/LAMB2	3
BP	GO:0072243	metanephric nephron epithelium development	0,00300285	0,029648801	ACAT1/ADIPOQ/LAMB2	3
BP	GO:0048880	sensory system development	0,00319677	0,031087094	CHRDL1/COL4A1/COL8A1/CRYAB/LAMB2/MYH10/RBP4/SERPINF1/SLC7A11/UNC45B	10

BP	GO:0033028	myeloid cell apoptotic process	0,00355746	0,033580845	ADIPOQ/CCL5/SLC7A11	3
BP	GO:0042391	regulation of membrane potential	0,00392745	0,036276094	ABAT/ATP1B2/BIN1/BNIP3/FHL1/FXYD1/GOT1/HEBP2/PARK7/SNTA1	10
BP	GO:0010907	positive regulation of glucose metabolic process	0,00417065	0,037579578	ACTN3/SLC25A12/SORBS1	3
BP	GO:0042572	retinol metabolic process	0,00417065	0,037579578	ADH4/ALDH1A1/RBP4	3
BP	GO:0106106	cold-induced thermogenesis	0,0043836	0,038953613	ACADL/ACTN3/ADIPOQ/ALDH1A1/EPAS1/GATM	6
BP	GO:0120161	regulation of cold-induced thermogenesis	0,0043836	0,038953613	ACADL/ACTN3/ADIPOQ/ALDH1A1/EPAS1/GATM	6
BP	GO:0060359	response to ammonium ion	0,00450135	0,03986245	ABAT/GLDC/GOT2/GPI/MAP1LC3A	5
BP	GO:0097164	ammonium ion metabolic process	0,0046232	0,040557865	ABAT/ACADL/AGMAT/BHMT/CPT1A/PARK7/PEBP1	7
BP	GO:0070206	protein trimerization	0,00464283	0,040557865	ADIPOQ/CD74/COL1A1/ITLN1	4
BP	GO:1990845	adaptive thermogenesis	0,00544835	0,046801317	ACADL/ACTN3/ADIPOQ/ALDH1A1/EPAS1/GATM	6
BP	GO:0042594	response to starvation	0,00554369	0,047304907	ACAT1/ADSSL1/GABARAPL2/MAP1LC3A/MYH13/OXCT1/PPARG	7
BP	GO:0032387	negative regulation of intracellular transport	0,00556404	0,047321883	ADIPOQ/CRYAB/PARK7/PKIG	4
BP	GO:0044270	cellular nitrogen compound catabolic process	0,00581787	0,049317924	ACADL/ACAT1/ACTN3/BHMT/GAPDH/GPD1/GPI/PABPC4/PFKM/PGK1/PGM1/PRKAG2/SLC25A12/TPI1/XDH	15
BP	GO:0006979	response to oxidative stress	0,00591663	0,049990695	ADIPOQ/BNIP3/COL1A1/CRYAB/EPAS1/FBLN5/MAP1LC3A/PARK7/PEBP1/PON2/SLC7A11/TPM1	12

CC	GO:0044449	contractile fiber part	1,6447E-19	4,73664E-17	ACTA1/ACTA2/ACTC1/BIN1/CRYAB/DES/MYBPC2/MYBPC3/MYH1/MYH13/MYH2/MYH4/MYH7/MYL1/MYL2/MYL3/MYL4/MYLPF/MYOZ1/TNNC1/TNNC2/TNNI1/TNNI2/TNNT1/TNNT3/TPM1/TPM3	27
CC	GO:0043292	contractile fiber	9,7961E-19	1,41064E-16	ACTA1/ACTA2/ACTC1/BIN1/CRYAB/DES/MYBPC2/MYBPC3/MYH1/MYH13/MYH2/MYH4/MYH7/MYL1/MYL2/MYL3/MYL4/MYLPF/MYOZ1/TNNC1/TNNC2/TNNI1/TNNI2/TNNT1/TNNT3/TPM1/TPM3	27
CC	GO:0030016	myofibril	5,1265E-17	4,51619E-15	ACTA1/ACTC1/BIN1/CRYAB/DES/MYBPC2/MYBPC3/MYH1/MYH13/MYH2/MYH4/MYH7/MYL1/MYL2/MYL3/MYL4/MYOZ1/TNNC1/TNNC2/TNNI1/TNNI2/TNNT1/TNNT3/TPM1/TPM3	25
CC	GO:0030017	sarcomere	6,2725E-17	4,51619E-15	ACTA1/ACTC1/BIN1/CRYAB/DES/MYBPC2/MYBPC3/MYH1/MYH2/MYH4/MYH7/MYL1/MYL2/MYL3/MYL4/MYOZ1/TNNC1/TNNC2/TNNI1/TNNI2/TNNT1/TNNT3/TPM1/TPM3	24
CC	GO:0015629	actin cytoskeleton	1,0525E-14	6,06229E-13	ACTA1/ACTA2/ACTC1/ACTN3/BIN1/CRYAB/MYBPC2/MYBPC3/MYH1/MYH10/MYH13/MYH2/MYH4/MYH7/MYL1/MYL2/MYL3/MYL4/MYLPF/MYOZ1/PGM1/SMTNL2/SORBS1/TNNC1/TNNC2/TNNI1/TNNI2/TNNT1/TNNT3/TPM1/TPM3	31
CC	GO:0005865	striated muscle thin filament	1,9595E-13	9,40582E-12	ACTA1/MYBPC2/MYBPC3/TNNC1/TNNC2/TNNI1/TNNI2/TNNT1/TNNT3/TPM1/TPM3	11

CC	GO:0016460	myosin II complex	3,0929E-13	1,17272E-11	MYBPC3/MYH1/MYH10/MYH13/MYH2/MYH4/MYH7/MYL1/MYL3/MYLPF	10
CC	GO:0036379	myofilament	3,2576E-13	1,17272E-11	ACTA1/MYBPC2/MYBPC3/TNNC1/TNNC2/TNNI1/TNNI2/TNNT1/TNNT3/TPM1/TPM3	11
CC	GO:0016459	myosin complex	3,7519E-13	1,20061E-11	MYBPC2/MYBPC3/MYH1/MYH10/MYH13/MYH2/MYH4/MYH7/MYL1/MYL2/MYL3/MYL4/MYLPF	13
CC	GO:0005859	muscle myosin complex	1,2186E-12	3,50949E-11	MYBPC3/MYH1/MYH13/MYH2/MYH4/MYH7/MYL1/MYL3/MYLPF	9
CC	GO:0032982	myosin filament	7,5316E-10	1,97191E-08	MYBPC2/MYBPC3/MYH1/MYH10/MYH13/MYH2/MYH4/MYH7	8
CC	GO:0031672	A band	7,9263E-07	1,90231E-05	CRYAB/MYBPC2/MYBPC3/MYH1/MYL2/MYL3/MYL4	7
CC	GO:0044420	extracellular matrix component	2,3387E-06	5,1811E-05	COL1A1/COL4A1/COL4A2/COL8A1/FBLN5/LAMB2/MFAP5	7
CC	GO:0032432	actin filament bundle	2,2807E-05	0,00046917	ACTA1/CRYAB/MYH10/MYH7/SORBS1/TPM1/TPM3	7
CC	GO:0042641	actomyosin	3,3688E-05	0,000646819	ACTA1/ACTC1/MYH10/MYH7/SORBS1/TPM1/TPM3	7
CC	GO:0043209	myelin sheath	5,7845E-05	0,001041202	CA2/CRYAB/GOT2/GPI/MBP/MDH2/PEBP1/PMP22/SLC25A12/SLC25A4	10
CC	GO:0031674	I band	6,7954E-05	0,001151228	ACTC1/BIN1/CRYAB/DES/MYBPC2/MYBPC3/MYH7/MYL3/MYOZ1	9
CC	GO:0098644	complex of collagen trimers	7,7727E-05	0,00124363	COL1A1/COL4A1/COL4A2/COL8A1	4
CC	GO:0001725	stress fiber	0,00011857	0,001707386	ACTA1/MYH10/MYH7/SORBS1/TPM1/TPM3	6
CC	GO:0097517	contractile actin filament bundle	0,00011857	0,001707386	ACTA1/MYH10/MYH7/SORBS1/TPM1/TPM3	6

CC	GO:0062023	collagen-containing extracellular matrix	0,00029563	0,004054367	ADIPOQ/ANXA6/CLEC3B/COL1A1/COL4A1/COL4A2/COL8A1/DPT/FBLN5/LAMB2/MFAP5/SERPINF1	12
CC	GO:0042383	sarcolemma	0,0006306	0,008255128	BIN1/CACNG1/DES/FXYD1/GOT2/SNTA1/SNTB1	7
CC	GO:0030018	Z disc	0,00115769	0,014496348	BIN1/CRYAB/DES/MYBPC2/MYBPC3/MYH7/MYOZ1	7
CC	GO:0031307	integral component of mitochondrial outer membrane	0,00130846	0,015143927	BNIP3/CPT1A/SYNJ2BP	3
CC	GO:0031012	extracellular matrix	0,00131458	0,015143927	ADIPOQ/ANXA6/CLEC3B/COL1A1/COL4A1/COL4A2/COL8A1/CRISPLD2/DPT/FBLN5/LAMB2/MFAP5/SERPINF1	13
CC	GO:0005884	actin filament	0,0015456	0,017120488	ACTA1/ACTC1/ACTN3/SMTNL2/TPM1/TPM3	6
CC	GO:0031306	intrinsic component of mitochondrial outer membrane	0,00164411	0,017507359	BNIP3/CPT1A/SYNJ2BP	3
CC	GO:0005581	collagen trimer	0,0017021	0,017507359	ADIPOQ/COL1A1/COL4A1/COL4A2/COL8A1	5
CC	GO:0030315	T-tubule	0,00245574	0,024388082	BIN1/CACNG1/FXYD1/GOT2	4
CC	GO:0031594	neuromuscular junction	0,00260649	0,025022321	DES/LAMB2/MYH10/SNTA1/SYNJ2BP	5
MF	GO:0003779	actin binding	4,5165E-12	1,74338E-09	ACTN3/ADSSL1/ANXA6/BIN1/MYBPC2/MYBPC3/MYH1/MYH10/MYH13/MYH2/MYH4/MYH7/MYL2/MYL3/MYL4/MYOZ1/SNTA1/SNTB1/SORBS1/TNNC1/TNNC2/TNNI1/TNNI2/TNNT3/TPM1/TPM3	26
MF	GO:0051015	actin filament binding	2,8097E-09	5,42273E-07	ADSSL1/ANXA6/BIN1/MYBPC2/MYBPC3/MYH1/MYH10/MYH13/MYH2/MYH4/MYH7/MYL4/TNNC1/TNNC2/TPM1/TPM3	16
MF	GO:0008307	structural constituent of muscle	8,5612E-09	1,10154E-06	ACTN3/ASPH/MYBPC2/MYBPC3/MYL1/MYL2/MYL3/MYLPF/TPM1	9

MF	GO:0050662	coenzyme binding	9,8025E-08	9,45938E-06	ABAT/ACADL/ACAT1/ACBD7/ADH4/AIFM3/ALDH1A1/FMO3/GAPDH/GLDC/GOT1/GOT2/GPD1/HLCS/OAT/PYGM/XDH	17
MF	GO:0005198	structural molecule activity	1,5661E-07	1,20903E-05	ACTA1/ACTN3/ADIPOQ/ASPH/COL1A1/COL4A1/COL4A2/COL8A1/CRYAB/DES/DPT/FBLN5/KRT6A/LAMB2/MBP/MFAP5/MYBPC2/MYBPC3/MYL1/MYL2/MYL3/MYLPF/PPL/PRPH/SNTA1/SNTB1/TPM1	27
MF	GO:0016769	transferase activity, transferring nitrogenous groups	2,9674E-07	1,90904E-05	ABAT/GAPDH/GATM/GOT1/GOT2/OAT	6
MF	GO:0000146	microfilament motor activity	8,5527E-06	0,000418627	MYH10/MYH13/MYH2/MYH4/MYH7	5
MF	GO:0048037	cofactor binding	8,6762E-06	0,000418627	ABAT/ACADL/ACAT1/ACBD7/ADH4/AIFM3/ALDH1A1/FMO3/GAPDH/GLDC/GOT1/GOT2/GPD1/GSTM4/HEBP2/HLCS/OAT/PYGM/XDH	19
MF	GO:0005516	calmodulin binding	2,5453E-05	0,001002267	MBP/MYH1/MYH10/MYH13/MYH2/MYH4/MYH7/SNTA1/SNTB1/SPA17	10
MF	GO:0019842	vitamin binding	2,5965E-05	0,001002267	ABAT/ADH4/GLDC/GOT1/GOT2/HLCS/OAT/PYGM/RBP4	9
MF	GO:0030170	pyridoxal phosphate binding	4,1582E-05	0,001337543	ABAT/GLDC/GOT1/GOT2/OAT/PYGM	6
MF	GO:0070279	vitamin B6 binding	4,1582E-05	0,001337543	ABAT/GLDC/GOT1/GOT2/OAT/PYGM	6
MF	GO:0017022	myosin binding	6,5709E-05	0,001951061	ACTA1/ACTC1/MYBPC3/MYL2/MYL3/MYL4	6
MF	GO:0005201	extracellular matrix structural constituent	8,9543E-05	0,002468839	ADIPOQ/COL1A1/COL4A1/COL4A2/COL8A1/DPT/FBLN5/LAMB2/MFAP5	9
MF	GO:0008483	transaminase activity	0,0001114	0,002866646	ABAT/GOT1/GOT2/OAT	4

MF	GO:0005509	calcium ion binding	0,00022812	0,005503429	ACTN3/ANXA6/ASPH/CLEC3B/FBLN5/ITLN1/MYL1/MYL2/MYL3/MYL4/MYLPF/PVALB/SLC25A12/TNNC1/TNNC2/TNNT1/TNNT3	17
MF	GO:0030020	extracellular matrix structural constituent conferring tensile strength	0,00089923	0,019283494	COL1A1/COL4A1/COL4A2/COL8A1	4
MF	GO:0043531	ADP binding	0,00089923	0,019283494	ACTA1/MYH10/PGK1/PRKAG2	4
MF	GO:0003774	motor activity	0,00127188	0,025839145	MYH1/MYH10/MYH13/MYH2/MYH4/MYH7/MYL3	7
MF	GO:0003785	actin monomer binding	0,00212657	0,041042796	MYL2/MYL3/MYL4	3

Supplementary Table 9: Gene ontology terms enriched by the top 280 DE genes in regenerative vs. wound-healing comparison, commonly identified by the analyses of both technologies

GO terms enriched by the top 150 up-regulated genes						
**ONTOLOGY**	**ID**	**Description**	**pvalue**	**p.adjust**	**geneID**	**Count**
BP	GO:0000280	nuclear division	3,1046E-29	5,3026E-26	AURKA/AURKB/BUB1/BUB1B/CCNA2/CCNB1/CCNB3/CDC20/CDCA5/CDCA8/CENPF/CHEK1/INCENP/KIF11/KIF22/KIF23/KIF2C/KIFC1/MAD2L1/MTBP/NCAPD3/NCAPG/NCAPH/NDC80/NEK2/NIPBL/NUSAP1/PDS5A/PLK1/PRC1/RAD51/RCC1/RMI1/RPA2/SMC2/SMC4/TOP2B/TPX2/TRIP13/ZWINT	40
BP	GO:0140014	mitotic nuclear division	5,7191E-28	4,8841E-25	AURKA/AURKB/BUB1/BUB1B/CCNA2/CCNB1/CCNB3/CDC20/CDCA5/CDCA8/CENPF/CHEK1/INCENP/KIF11/KIF22/KIF23/KIF2C/KIFC1/MAD2L1/MTBP/NCAPD3/NCAPG/NCAPH/NDC80/NEK2/NIPBL/NUSAP1/PDS5A/PLK1/PRC1/RCC1/SMC2/SMC4/TPX2/TRIP13/ZWINT	36
BP	GO:0098813	nuclear chromosome segregation	1,381E-26	5,8968E-24	AURKB/BUB1/BUB1B/CCNB1/CDC20/CDCA5/CDCA8/CENPF/DYNC1H1/FEN1/INCENP/KIF22/KIF23/KIF2C/KIFC1/MAD2L1/NCAPD3/NCAPG/NCAPH/NDC80/NEK2/NIPBL/NUSAP1/PDS5A/PLK1/PRC1/RMI1/SMC2/SMC4/TOP2B/TRIP13/ZWINT	32
BP	GO:0000819	sister chromatid segregation	2,3876E-26	8,1561E-24	AURKB/BUB1/BUB1B/CCNB1/CDC20/CDCA5/CDCA8/CENPF/FEN1/INCENP/KIF22/KIF23/KIF2C/KIFC1/MAD2L1/NCAPD3/NCAPG/NCAPH/NDC80/NEK2/NIPBL/NUSAP1/PDS5A/PLK1/PRC1/SMC2/SMC4/TOP2B/TRIP13/ZWINT	30
BP	GO:0010564	regulation of cell cycle process	9,3048E-20	1,4448E-17	APEX1/AURKA/AURKB/BUB1/BUB1B/CBX3/CCNA2/CCNB1/CCNB3/CDC20/CDC25A/CDC7/CDCA5/CENPF/CHEK1/DYNC1H1/EED/EZH2/FEN1/HAUS1/INCENP/KIF11/KIF20A/KIF23/MAD2L1/MTA3/MTBP/NDC80/NEK2/NIPBL/NUSAP1/PCNA/PLK1/PRC1/PRPF40A/RAD51/RCC1/RPA2/RRM2/TPX2/TRIP13	41
BP	GO:0071103	DNA conformation change	1,1248E-18	1,601E-16	ASF1B/CCNB1/CDCA5/CHAF1A/CHAF1B/GINS2/HELLS/HIST1H2BJ/HMGB3/MCM2/MCM4/MCM6/MCM7/NASP/NCAPD3/NCAPG/NCAPH/NIPBL/NUSAP1/RAD51/RPA2/SART3/SMC2/SMC4/TOP2B	25
BP	GO:0006260	DNA replication	4,1727E-16	5,0907E-14	CCNA2/CDC7/CHAF1A/CHAF1B/CHEK1/FEN1/GINS2/MCM2/MCM3/MCM4/MCM6/MCM7/NASP/NUCKS1/PCNA/PDS5A/POLA2/RAD51/RFC2/RFC4/RMI1/RPA2/RRM1/RRM2/SLBP	25
BP	GO:0007088	regulation of mitotic nuclear division	6,5663E-16	7,4768E-14	AURKA/AURKB/BUB1/BUB1B/CCNA2/CCNB1/CCNB3/CDC20/CDCA5/CENPF/CHEK1/KIF11/MAD2L1/MTBP/NDC80/NEK2/NIPBL/NUSAP1/PLK1/RCC1/TRIP13	21
BP	GO:0051983	regulation of chromosome segregation	1,1471E-13	1,0885E-11	AURKB/BUB1/BUB1B/CCNB1/CDC20/CDCA5/CENPF/DYNC1H1/FEN1/KIF2C/MAD2L1/NDC80/NEK2/NIPBL/PLK1/TRIP13	16
BP	GO:0007052	mitotic spindle organization	2,3213E-12	1,652E-10	AURKA/AURKB/CCNB1/CDC20/DYNC1H1/KIF11/KIF23/KIFC1/NDC80/NEK2/PLK1/PRC1/RCC1/STMN1/TPX2	15
BP	GO:1903046	meiotic cell cycle process	3,408E-12	2,2388E-10	AURKA/BUB1/BUB1B/CDC20/CDC25A/NCAPD3/NCAPH/PLK1/RAD51/RMI1/RPA2/SMC2/SMC4/TOP2B/TRIP13	15
BP	GO:0007094	mitotic spindle assembly checkpoint	1,9122E-11	8,8271E-10	AURKB/BUB1/BUB1B/CCNB1/CDC20/CENPF/MAD2L1/NDC80/PLK1/TRIP13	10
BP	GO:0071174	mitotic spindle checkpoint	1,9122E-11	8,8271E-10	AURKB/BUB1/BUB1B/CCNB1/CDC20/CENPF/MAD2L1/NDC80/PLK1/TRIP13	10
BP	GO:0006325	chromatin organization	4,1786E-11	1,6221E-09	ASF1B/ATAD2/AURKA/AURKB/BAZ1B/BRD3/CBX3/CCNA2/CCNB1/CHAF1A/CHAF1B/CHEK1/EED/EZH2/HELLS/HIST1H2AG/HIST1H2BJ/HMGB3/MBD3/MCM2/MTA3/NASP/NIPBL/NUCKS1/RNF168/SAFB/SART3/TRIM28/UHRF1/VRK1	30
BP	GO:1905819	negative regulation of chromosome separation	5,9007E-11	2,1444E-09	AURKB/BUB1/BUB1B/CCNB1/CDC20/CENPF/MAD2L1/NDC80/PLK1/TRIP13	10
BP	GO:0140013	meiotic nuclear division	8,0562E-11	2,8667E-09	AURKA/BUB1/BUB1B/CDC20/NCAPH/PLK1/RAD51/RMI1/RPA2/SMC2/SMC4/TOP2B/TRIP13	13

BP	GO:0033044	regulation of chromosome organization	1,2953E-10	4,3669E-09	ATAD2/AURKB/BUB1/BUB1B/CCNB1/CDC20/CDCA5/CENPF/CHEK1/EED/FEN1/MAD2L1/MCM2/NDC80/NEK2/NIPBL/PARP1/PLK1/SART3/TRIM28/TRIP13	21
BP	GO:1905818	regulation of chromosome separation	8,9136E-09	2,0032E-07	AURKB/BUB1/BUB1B/CCNB1/CDC20/CENPF/MAD2L1/NDC80/PLK1/TRIP13	10
BP	GO:0051310	metaphase plate congression	2,7733E-08	5,996E-07	CCNB1/CDCA5/CDCA8/CENPF/DYNC1H1/KIF22/KIF2C/KIFC1/NDC80	9
BP	GO:0016572	histone phosphorylation	1,596E-07	3,0978E-06	AURKA/AURKB/BAZ1B/CCNA2/CCNB1/CHEK1/VRK1	7
BP	GO:0050000	chromosome localization	1,8909E-07	3,5884E-06	CCNB1/CDCA5/CDCA8/CENPF/DYNC1H1/KIF22/KIF2C/KIFC1/NDC80	9
BP	GO:0061982	meiosis I cell cycle process	5,3739E-07	9,8694E-06	AURKA/CDC25A/PLK1/RAD51/RMI1/RPA2/TOP2B/TRIP13	8
BP	GO:0034502	protein localization to chromosome	5,7109E-07	1,0377E-05	AURKB/BUB1B/CDCA5/EZH2/MTBP/NDC80/NIPBL/PLK1/RPA2	9
BP	GO:0031145	anaphase-promoting complex-dependent catabolic process	9,3982E-07	1,6549E-05	AURKA/AURKB/BUB1B/CCNB1/CDC20/MAD2L1/PLK1	7
BP	GO:0065004	protein-DNA complex assembly	1,0579E-06	1,8438E-05	ASF1B/CENPF/CHAF1A/CHAF1B/HELLS/HIST1H2BJ/MCM2/NASP/PARP1/RAD51/RPA2/SART3	12
BP	GO:2001020	regulation of response to DNA damage stimulus	2,2249E-06	3,7256E-05	BCLAF1/CHEK1/PARP1/PCNA/PSMD10/RAD51/RAD51AP1/RAD9A/RNF168/RPA2/TRIM28/USP1	12
BP	GO:0071478	cellular response to radiation	3,4238E-06	5,5168E-05	AURKB/CDC25A/CHEK1/NIPBL/NUCKS1/PARP1/PBK/PCNA/RAD51/RAD51AP1/RAD9A	11
BP	GO:0071824	protein-DNA complex subunit organization	6,7603E-06	0,00010497	ASF1B/CENPF/CHAF1A/CHAF1B/HELLS/HIST1H2BJ/MCM2/NASP/PARP1/RAD51/RPA2/SART3	12
BP	GO:0042769	DNA damage response, detection of DNA damage	1,506E-05	0,00021874	PARP1/PCNA/RFC2/RFC4/RPA2/USP1	6
BP	GO:0007131	reciprocal meiotic recombination	3,2237E-05	0,00044049	RAD51/RMI1/RPA2/TOP2B/TRIP13	5
BP	GO:0009314	response to radiation	4,6682E-05	0,0006328	AURKB/CDC25A/CHEK1/FEN1/NIPBL/NUCKS1/PARP1/PBK/PCNA/RAD51/RAD51AP1/RAD9A/RNF168/RRM1/USP1	15
BP	GO:0000079	regulation of cyclin-dependent protein serine/threonine kinase activity	0,00035488	0,00401414	CCNA2/CCNB1/CCNB3/CDC25A/PLK1/PSMD10	6
BP	GO:0104004	cellular response to environmental stimulus	0,00046855	0,00501616	AURKB/CDC25A/CHEK1/NIPBL/NUCKS1/PARP1/PBK/PCNA/RAD51/RAD51AP1/RAD9A	11
BP	GO:0045814	negative regulation of gene expression, epigenetic	0,00056142	0,00588288	ATAD2/EED/EZH2/HELLS/MBD3/TRIM28	6
BP	GO:0030397	membrane disassembly	0,00068536	0,00705178	CCNB1/PLK1/VRK1	3
BP	GO:0051081	nuclear envelope disassembly	0,00068536	0,00705178	CCNB1/PLK1/VRK1	3
BP	GO:0060249	anatomical structure homeostasis	0,000894	0,00867369	APEX1/AURKB/FEN1/IQCB1/NEK2/PARP1/PCNA/POLA2/RAD51/RFC2/RFC4/RPA2	12
BP	GO:0001556	oocyte maturation	0,00090265	0,00867369	AURKA/CCNB1/TRIP13	3
BP	GO:0000077	DNA damage checkpoint	0,00090393	0,00867369	AURKA/CCNB1/CHEK1/PCNA/PLK1/RAD9A/RPA2	7
BP	GO:0006342	chromatin silencing	0,00117269	0,01088566	ATAD2/EED/HELLS/MBD3/TRIM28	5
BP	GO:0097711	ciliary basal body-plasma membrane docking	0,00143606	0,01325837	B9D1/DYNC1H1/HAUS1/IQCB1/NEK2/PLK1	6

BP	GO:0042770	signal transduction in response to DNA damage	0,0021466	0,01919575	AURKA/CCNB1/CHEK1/PCNA/PLK1/PSMD10	6
BP	GO:0032886	regulation of microtubule-based process	0,00224919	0,01990473	AURKA/CHEK1/DYNC1H1/KIF11/NEK2/PLK1/STMN1/TPX2	8
BP	GO:0007292	female gamete generation	0,00235104	0,02033725	AURKA/CCNB1/NCAPH/PLK1/TRIP13	5
BP	GO:0043388	positive regulation of DNA binding	0,0023576	0,02033725	CDCA5/NIPBL/PARP1/TRIM28	4
BP	GO:1904666	regulation of ubiquitin protein ligase activity	0,00262411	0,02218799	CDC20/MAD2L1/PLK1	3
BP	GO:0051098	regulation of binding	0,00281448	0,02368047	AURKA/AURKB/CDCA5/CTHRC1/NEK2/NIPBL/NUCKS1/PARP1/PLK1/STMN1/TRIM28	11
BP	GO:1905269	positive regulation of chromatin organization	0,00367695	0,02990587	CCNB1/EED/NIPBL/SART3/TRIM28	5
BP	GO:2000241	regulation of reproductive process	0,00394116	0,03145564	AURKA/CDC20/CDC25A/TRIP13/WDR77	5
BP	GO:0097421	liver regeneration	0,00559078	0,04206629	AURKA/EZH2/PCNA	3
BP	GO:0019886	antigen processing and presentation of exogenous peptide antigen via MHC class II	0,00581888	0,04359055	DYNC1H1/KIF11/KIF22/KIF23/KIF2C	5
BP	GO:0051606	detection of stimulus	0,00649023	0,04798837	PARP1/PCNA/RFC2/RFC4/RPA2/USP1	6
CC	GO:0000228	nuclear chromosome	8,7177E-31	1,8133E-28	APEX1/ASF1B/AURKA/AURKB/BAZ1B/BUB1/BUB1B/CBX3/CCNB1/CDCA5/CHAF1A/CHAF1B/CHEK1/EED/EZH2/FEN1/GINS2/HIST1H2AG/HMGB3/INCENP/LRPPRC/MBD3/MCM2/MCM3/MCM4/MCM6/MCM7/MTA3/NASP/NCAPD3/NCAPH/NDC80/NEK2/NIPBL/NUCKS1/PARP1/PCNA/PLK1/POLA2/RAD51/RAD51AP1/RAD9A/RCC1/RPA2/SMC2/TRIM28/UHRF1	47
CC	GO:0044454	nuclear chromosome part	7,8222E-28	7,2152E-26	APEX1/ASF1B/AURKA/AURKB/BAZ1B/BUB1/BUB1B/CBX3/CCNB1/CDCA5/CHAF1A/CHAF1B/EED/EZH2/FEN1/GINS2/HIST1H2AG/HMGB3/INCENP/MBD3/MCM2/MCM3/MCM4/MCM6/MCM7/MTA3/NASP/NCAPD3/NCAPH/NDC80/NIPBL/NUCKS1/PARP1/PCNA/PLK1/POLA2/RAD51/RAD51AP1/RAD9A/RCC1/RPA2/TRIM28/UHRF1	43
CC	GO:0098687	chromosomal region	1,0407E-27	7,2152E-26	APEX1/AURKA/AURKB/BAZ1B/BUB1/BUB1B/CBX3/CCNB1/CDCA5/CDCA8/CENPF/CHEK1/FEN1/HELLS/INCENP/KIF22/KIF2C/MAD2L1/MCM2/MCM3/MCM4/MCM6/MCM7/MTBP/NCAPD3/NCAPG/NDC80/NEK2/NUP133/NUP43/PARP1/PCNA/PDS5A/PLK1/RAD51/RPA2/ZWINT	37
CC	GO:0000793	condensed chromosome	1,6929E-25	8,8029E-24	AURKA/AURKB/BAZ1B/BUB1/BUB1B/CBX3/CCNB1/CDCA5/CENPF/CHEK1/INCENP/KIF2C/LRPPRC/MAD2L1/NCAPD3/NCAPG/NCAPH/NDC80/NEK2/NUP133/NUP43/PLK1/RAD51/RAD9A/RCC1/RPA2/SMC2/SMC4/ZWINT	29
CC	GO:0000785	chromatin	5,6968E-21	2,3699E-19	ASF1B/BAZ1B/CBX3/CDCA5/CENPF/CHAF1A/CHAF1B/CHEK1/EED/EZH2/HELLS/HIST1H2AG/HIST1H2BJ/HMGB3/INCENP/KIF22/MBD3/MCM2/MCM7/MTA3/MTBP/NASP/NCAPD3/NIPBL/NUCKS1/PCNA/PDS5A/PLK1/RAD51/RAD51AP1/RCC1/RPA2/TMPO/TOP2B/TRIM28/UHRF1	36
CC	GO:0000775	chromosome, centromeric region	5,1542E-20	1,7868E-18	AURKA/AURKB/BAZ1B/BUB1/BUB1B/CBX3/CCNB1/CDCA5/CDCA8/CENPF/HELLS/INCENP/KIF22/KIF2C/MAD2L1/MTBP/NCAPD3/NCAPG/NDC80/NEK2/NUP133/NUP43/PDS5A/PLK1/ZWINT	25
CC	GO:0000794	condensed nuclear chromosome	3,4167E-19	1,0152E-17	AURKA/AURKB/BUB1/BUB1B/CCNB1/CHEK1/INCENP/LRPPRC/NCAPD3/NCAPH/NDC80/NEK2/PLK1/RAD51/RAD9A/RCC1/RPA2	17

CC	GO:0000779	condensed chromosome, centromeric region	9,9741E-17	2,5933E-15	AURKA/AURKB/BUB1/BUB1B/CBX3/CCNB1/CENPF/INCENP/KIF2C/MAD2L1/NCAPD3/NCAPG/NDC80/NEK2/NUP133/NUP43/PLK1/ZWINT	18
CC	GO:0005819	spindle	1,2305E-13	2,8439E-12	AURKA/AURKB/BUB1B/CBX3/CCNB1/CDC20/CDC7/CDCA8/CENPF/HAUS1/INCENP/IQCB1/KIF11/KIF20A/KIF22/KIF23/KIFC1/MAD2L1/NEK2/NUSAP1/PLK1/PRC1/TPX2/VRK1	24
CC	GO:0000776	kinetochore	1,1432E-12	2,3779E-11	AURKB/BUB1/BUB1B/CCNB1/CENPF/INCENP/KIF22/KIF2C/MAD2L1/MTBP/NDC80/NEK2/NUP133/NUP43/PLK1/ZWINT	16
CC	GO:0000777	condensed chromosome kinetochore	4,9936E-11	9,4425E-10	BUB1/BUB1B/CCNB1/CENPF/INCENP/KIF2C/MAD2L1/NDC80/NEK2/NUP133/NUP43/PLK1/ZWINT	13
CC	GO:0000790	nuclear chromatin	2,5773E-10	4,4673E-09	ASF1B/CBX3/CDCA5/CHAF1A/CHAF1B/EED/EZH2/HIST1H2AG/HMGB3/MBD3/MTA3/NASP/NCAPD3/NIPBL/NUCKS1/RAD51/RAD51AP1/RCC1/TRIM28/UHRF1	20
CC	GO:0000784	nuclear chromosome, telomeric region	1,2718E-09	2,0348E-08	APEX1/CBX3/FEN1/MCM2/MCM3/MCM4/MCM6/MCM7/PARP1/PCNA/RAD51/RPA2	12
CC	GO:0000780	condensed nuclear chromosome, centromeric region	1,3921E-09	2,0683E-08	AURKA/AURKB/BUB1/BUB1B/CCNB1/NDC80/PLK1	7
CC	GO:0000781	chromosome, telomeric region	3,6002E-09	4,9923E-08	APEX1/CBX3/CHEK1/FEN1/MCM2/MCM3/MCM4/MCM6/MCM7/PARP1/PCNA/RAD51/RPA2	13
CC	GO:0072686	mitotic spindle	6,4038E-09	8,325E-08	AURKA/AURKB/CDC7/IQCB1/KIF11/KIF22/KIF23/KIFC1/MAD2L1/NUSAP1/TPX2	11
CC	GO:0005874	microtubule	1,0844E-08	1,3268E-07	APPBP2/AURKA/AURKB/DYNC1H1/HAUS1/INCENP/KIF11/KIF20A/KIF22/KIF23/KIF2C/KIFC1/LRPPRC/NEK2/NUSAP1/PLK1/PRC1/STMN1/TPX2	19
CC	GO:0000792	heterochromatin	2,7176E-08	3,1403E-07	BAZ1B/CBX3/EED/HELLS/INCENP/MBD3/NCAPD3/TOP2B/TRIM28/UHRF1	10
CC	GO:0005815	microtubule organizing center	7,174E-08	7,8536E-07	ANKRD26/APEX1/AURKA/AURKB/B9D1/BUB1B/CCNB1/CCNB3/CDC20/CENPF/CHEK1/DYNC1H1/HAUS1/IQCB1/KIF23/KIF2C/KIFC1/MCM3/NDC80/NEK2/PCNA/PLK1/RAD51/SASS6/TPX2	25
CC	GO:0000922	spindle pole	9,2206E-08	9,4535E-07	AURKA/AURKB/CCNB1/CDC20/CENPF/HAUS1/KIF11/MAD2L1/NEK2/PLK1/PRC1/TPX2	12
CC	GO:0005813	centrosome	9,5444E-08	9,4535E-07	ANKRD26/APEX1/AURKA/AURKB/B9D1/CCNB1/CCNB3/CDC20/CENPF/CHEK1/DYNC1H1/HAUS1/IQCB1/KIF23/KIF2C/MCM3/NDC80/NEK2/PCNA/PLK1/SASS6	21
CC	GO:0005875	microtubule associated complex	1,0268E-07	9,7083E-07	APPBP2/AURKA/AURKB/CDCA8/DYNC1H1/HAUS1/KIF11/KIF20A/KIF22/KIF23/KIF2C/KIFC1	12
CC	GO:0005657	replication fork	1,3629E-07	1,2325E-06	BAZ1B/GINS2/MCM3/PCNA/POLA2/RFC2/RFC4/RPA2/UHRF1	9
CC	GO:0044815	DNA packaging complex	1,6345E-07	1,4165E-06	HIST1H2AG/HIST1H2BJ/NCAPD3/NCAPG/NCAPH/SMC2/SMC4	7
CC	GO:0000778	condensed nuclear chromosome kinetochore	4,297E-07	3,5751E-06	BUB1/BUB1B/CCNB1/NDC80/PLK1	5
CC	GO:0045120	pronucleus	1,7908E-06	1,4326E-05	AURKA/CCNA2/CENPF/EED/EZH2	5
CC	GO:0005721	pericentric heterochromatin	2,6515E-06	2,0426E-05	BAZ1B/CBX3/HELLS/INCENP/NCAPD3	5
CC	GO:0099513	polymeric cytoskeletal fiber	5,8196E-06	4,3231E-05	APPBP2/AURKA/AURKB/DYNC1H1/HAUS1/INCENP/KIF11/KIF20A/KIF22/KIF23/KIF2C/KIFC1/LRPPRC/NEK2/NUSAP1/PLK1/PRC1/STMN1/TPX2	19
CC	GO:0005871	kinesin complex	1,2091E-05	8,6722E-05	KIF11/KIF20A/KIF22/KIF23/KIF2C/KIFC1	6
CC	GO:0043596	nuclear replication fork	1,7257E-05	0,00011965	BAZ1B/GINS2/MCM3/PCNA/POLA2/RPA2	6
CC	GO:0045171	intercellular bridge	2,0436E-05	0,00013712	CDC7/CDCA8/IQCB1/KIF20A/KIF23/TPX2	6
CC	GO:0005876	spindle microtubule	2,4071E-05	0,00015646	AURKA/AURKB/KIF11/NUSAP1/PLK1/PRC1	6
CC	GO:0030496	midbody	3,3313E-05	0,00020997	AURKA/AURKB/CDCA8/CENPF/INCENP/KIF20A/KIF23/NEK2/PLK1/PRC1	10
CC	GO:0005720	nuclear heterochromatin	5,0356E-05	0,00030806	CBX3/EED/NCAPD3/TRIM28/UHRF1	5
CC	GO:0032993	protein-DNA complex	0,00010178	0,00060485	GINS2/HIST1H2AG/HIST1H2BJ/MCM3/PARP1/PCNA/POLA2/RPA2	8
CC	GO:0010369	chromocenter	0,00061216	0,00351268	AURKB/CDCA8/INCENP	3

CC	GO:0030894	replisome	0,00062485	0,00351268	MCM3/PCNA/POLA2/RPA2	4
CC	GO:0090734	site of DNA damage	0,00089541	0,0049012	CBX3/PARP1/RAD51/RNF168/RPA2	5
CC	GO:0051233	spindle midzone	0,00097424	0,00519592	AURKA/AURKB/CDCA8/PLK1	4
CC	GO:0005635	nuclear envelope	0,00348734	0,01813417	CBX3/CENPF/CSE1L/LRPPRC/MAD2L1/MCM3AP/NUP133/NUP43/PARP1/RCC1/RRM1/TMPO	12
CC	GO:0000791	euchromatin	0,00438601	0,02225098	CBX3/TRIM28/UHRF1	3
CC	GO:0043601	nuclear replisome	0,00501931	0,02485753	MCM3/POLA2/RPA2	3
CC	GO:0016604	nuclear body	0,00643878	0,03114575	APEX1/BAZ1B/BCLAF1/CCNB3/INCENP/KIF22/PCNA/PRPF40A/RAD51/RMI1/RPA2/SART3/SLTM/SMC4/SRRM2/ZWINT	16
CC	GO:0031080	nuclear pore outer ring	0,010629	0,04912959	NUP133/NUP43	2
CC	GO:0035098	ESC/E(Z) complex	0,010629	0,04912959	EED/EZH2	2
CC	GO:0000307	cyclin-dependent protein kinase holoenzyme complex	0,01097332	0,0496185	CCNA2/CCNB1/CCNB3	3
MF	GO:0003697	single-stranded DNA binding	1,4645E-11	4,13E-09	LONP1/LRPPRC/MCM4/MCM6/MCM7/NEIL3/NUCKS1/POLR3C/RAD51/RAD51AP1/RPA2/SMC2/SMC4/TSN	14
MF	GO:0003682	chromatin binding	7,8791E-10	1,111E-07	APEX1/ATAD2/BRD3/CDCA5/CENPF/CHAF1A/CHAF1B/EED/EZH2/MBD3/MTA3/NCAPD3/NCAPH/NIPBL/NUCKS1/PCNA/RAD51/RCC1/RNF168/SAFB/TOP2B/TRIM28/UHRF1/VRK1	24
MF	GO:0035173	histone kinase activity	1,5879E-08	1,4926E-06	AURKA/AURKB/BAZ1B/CCNB1/CHEK1/VRK1	6
MF	GO:0042393	histone binding	1,2862E-07	9,0674E-06	ASF1B/ATAD2/BAZ1B/BRD3/CHAF1B/MCM2/NASP/NCAPD3/RCC1/RNF168/SART3/UHRF1/VRK1	13
MF	GO:0016887	ATPase activity	9,3798E-07	5,2902E-05	ATAD2/DYNC1H1/HSPA14/KIF11/KIF20A/KIF22/KIF23/KIF2C/KIFC1/LONP1/MCM4/MCM6/MCM7/MDN1/RAD51/RFC2/RFC4/TOP2B	18
MF	GO:0140097	catalytic activity, acting on DNA	1,1371E-06	5,3443E-05	APEX1/FEN1/GINS2/MCM4/MCM6/MCM7/NEIL3/PCNA/POLA2/RAD51/RAD9A/TOP2B	12
MF	GO:0003777	microtubule motor activity	3,3934E-06	0,00013671	APPBP2/DYNC1H1/KIF11/KIF20A/KIF22/KIF23/KIF2C/KIFC1	8
MF	GO:0008094	DNA-dependent ATPase activity	6,1218E-05	0,00215793	MCM4/MCM6/MCM7/RAD51/RFC2/RFC4/TOP2B	7
MF	GO:0031491	nucleosome binding	8,9205E-05	0,00279508	EED/MBD3/RCC1/RNF168/UHRF1/VRK1	6
MF	GO:0003774	motor activity	0,00014528	0,00409697	APPBP2/DYNC1H1/KIF11/KIF20A/KIF22/KIF23/KIF2C/KIFC1	8
MF	GO:0008017	microtubule binding	0,00018488	0,00455043	KIF11/KIF20A/KIF22/KIF23/KIF2C/KIFC1/LRPPRC/NUSAP1/PLK1/PRC1	10
MF	GO:0003690	double-stranded DNA binding	0,00019364	0,00455043	APEX1/EED/ETV4/EZH2/FEN1/HMGB3/KIF2C/MBD3/MCM2/NEIL3/NUCKS1/PARP1/PCNA/RAD51/RAD51AP1/RPA2/SAFB/SMAD5/UHRF1	19
MF	GO:0042826	histone deacetylase binding	0,00039949	0,0086658	CDC20/KPNA2/MTA3/NIPBL/PARP1/RAD9A/TOP2B	7
MF	GO:0043142	single-stranded DNA-dependent ATPase activity	0,00049107	0,00989158	RAD51/RFC2/RFC4	3
MF	GO:0016799	hydrolase activity, hydrolyzing N-glycosyl compounds	0,00066705	0,01254047	APEX1/NEIL3/PCNA	3
MF	GO:0015631	tubulin binding	0,00073865	0,01301878	KIF11/KIF20A/KIF22/KIF23/KIF2C/KIFC1/LRPPRC/NUSAP1/PLK1/PRC1/STMN1	11
MF	GO:0016895	exodeoxyribonuclease activity, producing 5'-phosphomonoesters	0,00112841	0,01871836	APEX1/FEN1/RAD9A	3
MF	GO:0004672	protein kinase activity	0,00127148	0,01905256	AURKA/AURKB/BAZ1B/BUB1/BUB1B/CCNA2/CCNB1/CCNB3/CDC7/CHEK1/NEK2/PBK/PLK1/TRIM28/VRK1	15
MF	GO:0003684	damaged DNA binding	0,00135576	0,01905256	APEX1/FEN1/NEIL3/PCNA/RPA2	5

MF	GO:0008327	methyl-CpG binding	0,00141881	0,01905256	MBD3/UHRF1/WDR77	3
MF	GO:0070182	DNA polymerase binding	0,00141881	0,01905256	LONP1/PCNA/RAD51	3
MF	GO:0004529	exodeoxyribonuclease activity	0,0017521	0,02245871	APEX1/FEN1/RAD9A	3
MF	GO:0042623	ATPase activity, coupled	0,00183203	0,02246232	DYNC1H1/HSPA14/KIF11/LONP1/MCM4/MCM6/MCM7/RAD51/RFC2/RFC4/TOP2B	11
MF	GO:0004386	helicase activity	0,00205114	0,02410094	GINS2/HELLS/MCM2/MCM3/MCM4/MCM6/MCM7	7
MF	GO:0003678	DNA helicase activity	0,0025407	0,02865907	GINS2/MCM4/MCM6/MCM7	4
MF	GO:0016773	phosphotransferase activity, alcohol group as acceptor	0,00323603	0,03410151	AURKA/AURKB/BAZ1B/BUB1/BUB1B/CCNA2/CCNB1/CCNB3/CDC7/CHEK1/NEK2/NOL9/PBK/PLK1/TRIM28/VRK1	16
MF	GO:0019901	protein kinase binding	0,00326504	0,03410151	AURKA/CCNA2/CCNB1/CCNB3/CDC25A/KIF11/KIF20A/PARP1/PCNA/PLK1/PRC1/RAD9A/TOP2B/TPX2/VRK1	15
MF	GO:0008022	protein C-terminus binding	0,00340495	0,03429269	CDC20/CENPF/FBLN1/LAMA1/MAD2L1/NIPBL/RAD51/TOP2B	8
MF	GO:0019900	kinase binding	0,00392509	0,0381681	AURKA/AURKB/CCNA2/CCNB1/CCNB3/CDC25A/KIF11/KIF20A/PARP1/PCNA/PLK1/PRC1/RAD9A/TOP2B/TPX2/VRK1	16
MF	GO:0016796	exonuclease activity, active with either ribo- or deoxyribonucleic acids and producing 5'-phosphomonoesters	0,00496701	0,04668992	APEX1/DIS3/FEN1/RAD9A	4
**GO terms enriched by the top 128 down-regulated genes**						
**ONTOLOGY**	**ID**	**Description**	**pvalue**	**p.adjust**	**geneID**	**Count**
BP	GO:0030049	muscle filament sliding	3,564E-27	4,4122E-24	ACTA1/ACTC1/ACTN3/DES/MYBPC2/MYBPC3/MYH7/MYL1/MYL2/MYL3/MYL4/TNNC1/TNNC2/TNNI1/TNNI2/TNNT1/TNNT3/TPM1/TPM3	19
BP	GO:0033275	actin-myosin filament sliding	3,564E-27	4,4122E-24	ACTA1/ACTC1/ACTN3/DES/MYBPC2/MYBPC3/MYH7/MYL1/MYL2/MYL3/MYL4/TNNC1/TNNC2/TNNI1/TNNI2/TNNT1/TNNT3/TPM1/TPM3	19
BP	GO:0006936	muscle contraction	1,5322E-18	9,4845E-16	ACTA1/ACTA2/ACTC1/ACTN3/ALDOA/CRYAB/DES/FXYD1/GAMT/MYBPC2/MYBPC3/MYH1/MYH7/MYL1/MYL2/MYL3/MYL4/PTGS2/TNNC1/TNNC2/TNNI1/TNNI2/TNNT1/TNNT3/TPM1/TPM3	26
BP	GO:0008015	blood circulation	3,0574E-11	9,4627E-09	ACTA2/ACTC1/ADIPOQ/DES/EGFR/FXYD1/MYBPC3/MYH7/MYL1/MYL2/MYL3/MYL4/PTGS2/PTP4A3/RCAN1/SGK1/TNNC1/TNNI2/TNNT1/TNNT3/TPM1	21
BP	GO:0006937	regulation of muscle contraction	1,4987E-10	3,7107E-08	ACTN3/MYBPC3/MYH7/MYL2/MYL3/PTGS2/TNNC1/TNNC2/TNNI1/TNNI2/TNNT1/TNNT3/TPM1	13
BP	GO:0055008	cardiac muscle tissue morphogenesis	1,191E-08	1,9659E-06	ACTC1/MYBPC2/MYBPC3/MYH7/MYL2/MYL3/TNNC1/TNNI1/TPM1	9
BP	GO:0016053	organic acid biosynthetic process	2,48E-08	3,4114E-06	ACADL/ACTN3/ADIPOQ/ALDOA/BHMT/CD74/FBP1/GAMT/GPD1/GSTM4/HSD17B8/OAT/PFKM/PGK1/PGM1/PTGS2/UGDH/XBP1	18
BP	GO:0046394	carboxylic acid biosynthetic process	2,48E-08	3,4114E-06	ACADL/ACTN3/ADIPOQ/ALDOA/BHMT/CD74/FBP1/GAMT/GPD1/GSTM4/HSD17B8/OAT/PFKM/PGK1/PGM1/PTGS2/UGDH/XBP1	18
BP	GO:0030239	myofibril assembly	3,0348E-08	3,757E-06	ACTA1/ACTC1/MYBPC2/MYBPC3/MYL2/MYOZ1/TNNT1/TNNT3/TPM1	9
BP	GO:0010927	cellular component assembly involved in morphogenesis	6,7718E-08	6,9863E-06	ACTA1/ACTC1/MYBPC2/MYBPC3/MYL2/MYOZ1/PMP22/TNNT1/TNNT3/TPM1	10
BP	GO:0015669	gas transport	3,4002E-07	2,8063E-05	HBA2/HBE1/HBZ/MYC/RHAG	5
BP	GO:0050879	multicellular organismal movement	5,8978E-07	4,4251E-05	ACTN3/MYH7/TNNC1/TNNC2/TNNI2/TNNT1/TNNT3	7

BP	GO:0050881	musculoskeletal movement	5,8978E-07	4,4251E-05	ACTN3/MYH7/TNNC1/TNNC2/TNNI2/TNNT1/TNNT3	7
BP	GO:0046031	ADP metabolic process	1,908E-06	0,00012768	ACTN3/AK1/ALDOA/FBP1/GPD1/PFKM/PGK1/PGM1	8
BP	GO:0006165	nucleoside diphosphate phosphorylation	2,7516E-06	0,00017564	ACTN3/AK1/ALDOA/FBP1/GPD1/PFKM/PGK1/PGM1	8
BP	GO:0006091	generation of precursor metabolites and energy	6,4773E-06	0,00036776	ACTN3/ADIPOQ/ALDOA/COX7A2L/FBP1/GFPT2/GPD1/GYG1/MYC/OXCT1/PFKM/PGK1/PGM1/PYGM/SLC25A4/UGDH	16
BP	GO:0055002	striated muscle cell development	9,0079E-06	0,00044217	ACTA1/ACTC1/MYBPC2/MYBPC3/MYL2/MYOZ1/TNNT1/TNNT3/TPM1	9
BP	GO:0072525	pyridine-containing compound biosynthetic process	9,9925E-06	0,00045818	ACTN3/ALDOA/FBP1/GPD1/PFKM/PGK1/PGM1/PTGS2	8
BP	GO:0060537	muscle tissue development	1,0427E-05	0,00046943	ACTA1/ACTC1/ACTN3/EGR1/MYBPC2/MYBPC3/MYH7/MYL2/MYL3/TNNC1/TNNI1/TPM1/ZFAND5	13
BP	GO:0043462	regulation of ATPase activity	1,3552E-05	0,00056874	DNAJB1/MYBPC3/MYL3/MYL4/TNNC1/TNNT3/TPM1	7
BP	GO:0051259	protein complex oligomerization	1,4577E-05	0,00059167	ACADL/ADIPOQ/ALDOA/ARG1/CD74/CRYAB/FBP1/HBA2/HBE1/HBZ/HSD17B8/MMP3/OAT/PFKM/RIOK3/SQSTM1	16
BP	GO:0009636	response to toxic substance	1,624E-05	0,00063824	ACTC1/ADIPOQ/ARG1/BCL2L1/CRYAB/DUSP1/EGR1/HBA2/HBE1/HBZ/MBP/OXCT1/PON2/PRDX6/PTGS2	15
BP	GO:0044283	small molecule biosynthetic process	3,4872E-05	0,00126975	ACADL/ACTN3/ADIPOQ/ALDOA/BHMT/CD74/EGR1/FBP1/GAMT/GPD1/GSTM4/HSD17B8/OAT/PFKM/PGK1/PGM1/PTGS2/UGDH/XBP1	19
BP	GO:0006112	energy reserve metabolic process	3,6658E-05	0,00129744	GFPT2/GYG1/MYC/PFKM/PGM1/PYGM	6
BP	GO:0006754	ATP biosynthetic process	4,2899E-05	0,00145503	ACTN3/ALDOA/FBP1/GPD1/PFKM/PGK1/PGM1	7
BP	GO:0097435	supramolecular fiber organization	5,4691E-05	0,00180553	ACTA1/ACTC1/ALDOA/ARHGAP18/CRYAB/DES/MFAP5/MYBPC2/MYBPC3/MYL2/MYOZ1/RND3/TNNT1/TNNT3/TPM1/TPM3/TPPP3	17
BP	GO:0016052	carbohydrate catabolic process	9,5995E-05	0,0026706	ACTN3/ALDOA/FBP1/GPD1/PFKM/PGK1/PGM1/PYGM	8
BP	GO:0031032	actomyosin structure organization	9,881E-05	0,00271836	ACTA1/ACTC1/MYBPC2/MYBPC3/MYL2/MYOZ1/TNNT1/TNNT3/TPM1	9
BP	GO:0072524	pyridine-containing compound metabolic process	0,0001171	0,00315149	ACTN3/ALDOA/FBP1/GPD1/PFKM/PGK1/PGM1/PTGS2	8
BP	GO:0006094	gluconeogenesis	0,00020099	0,00507796	ADIPOQ/ALDOA/FBP1/GPD1/PGK1/PGM1	6
BP	GO:0019320	hexose catabolic process	0,00020732	0,00518506	ACTN3/ALDOA/PFKM/PGK1/PGM1	5
BP	GO:0072215	regulation of metanephros development	0,00028791	0,00698885	ADIPOQ/EGR1/MYC	3
BP	GO:0032722	positive regulation of chemokine production	0,00029778	0,00708939	ADIPOQ/CD74/EGR1/MBP	4
BP	GO:0042744	hydrogen peroxide catabolic process	0,00029778	0,00708939	HBA2/HBE1/HBZ/PRDX6	4
BP	GO:0032781	positive regulation of ATPase activity	0,00032743	0,00764828	DNAJB1/MYBPC3/MYL3/MYL4/TPM1	5
BP	GO:1902532	negative regulation of intracellular signal transduction	0,00039095	0,00882081	ACTN3/ADIPOQ/BCL2L1/CD74/DUSP1/DUSP5/FBP1/MYC/PTGS2/RCAN1/RIOK3/SOCS1/XBP1	13
BP	GO:1902106	negative regulation of leukocyte differentiation	0,00040429	0,00892191	ADIPOQ/CD74/IRF1/MYC/SOCS1	5
BP	GO:0001101	response to acid chemical	0,00041439	0,00892191	ADIPOQ/ARG1/BCL2L1/DUSP1/EGFR/EGR1/PTGS2/SERPINF1/SOCS1/XBP1	10

BP	GO:0010226	response to lithium ion	0,00051726	0,01024593	ACTA1/CDKN1B/PTGS2	3
BP	GO:0051195	negative regulation of cofactor metabolic process	0,00051726	0,01024593	ACTN3/FBP1/MMP3	3
BP	GO:1904706	negative regulation of vascular smooth muscle cell proliferation	0,00051726	0,01024593	ADIPOQ/CDKN1B/TPM1	3
BP	GO:0015893	drug transport	0,00056739	0,01097547	ARG1/HBA2/HBE1/HBZ/MYC/RHAG/SLC25A4	7
BP	GO:0046434	organophosphate catabolic process	0,00059635	0,01135822	ACTN3/ALDOA/FBP1/GPD1/PFKM/PGK1/PGM1/PRDX6	8
BP	GO:0005980	glycogen catabolic process	0,00066568	0,01211928	PFKM/PGM1/PYGM	3
BP	GO:0006525	arginine metabolic process	0,00066568	0,01211928	AGMAT/ARG1/OAT	3
BP	GO:0046716	muscle cell cellular homeostasis	0,00066568	0,01211928	ALDOA/PFKM/PGK1	3
BP	GO:0030334	regulation of cell migration	0,0007759	0,0138211	ADIPOQ/CD74/CITED2/CYR61/DUSP1/EGFR/FGFBP1/MMP3/PTGS2/RND3/SEMA3F/SERPINE1/SERPINF1/SGK1/TPM1/XBP1	16
BP	GO:0007162	negative regulation of cell adhesion	0,00090684	0,0159243	ADIPOQ/ARG1/CD74/DUSP1/IRF1/MBP/SERPINE1/SOCS1	8
BP	GO:0051384	response to glucocorticoid	0,0009753	0,016887	ADIPOQ/ARG1/DUSP1/EGFR/PTGS2/SERPINF1	6
BP	GO:0040008	regulation of growth	0,0010574	0,01781037	ACTN3/BCL2L1/CDKN1B/CRYAB/CYR61/EGFR/FBP1/FHL1/GAMT/MYL2/SEMA3F/SGK1/SOCS1/SQSTM1	14
BP	GO:0042698	ovulation cycle	0,00116831	0,01915714	EGFR/EGR1/MMP19/SERPINF1	4
BP	GO:0009108	coenzyme biosynthetic process	0,00150993	0,02361449	ACTN3/ALDOA/FBP1/GPD1/PFKM/PGK1/PGM1/PTGS2	8
BP	GO:0044282	small molecule catabolic process	0,00175141	0,02642348	ACADL/ACTN3/ADIPOQ/ALDOA/ARG1/FAAH/OAT/OXCT1/PFKM/PGK1/PGM1	11
BP	GO:0044042	glucan metabolic process	0,0017822	0,02642348	GYG1/PFKM/PGM1/PYGM	4
BP	GO:0017001	antibiotic catabolic process	0,00196492	0,02861842	HBA2/HBE1/HBZ/PRDX6	4
BP	GO:0062012	regulation of small molecule metabolic process	0,00201406	0,02916262	ACADL/ACTN3/ADIPOQ/BHMT/COX7A2L/EGR1/FBP1/GPD1/PTGS2	9
BP	GO:0002790	peptide secretion	0,00206823	0,02977289	ARL4D/CD74/EGFR/FCN1/MBP/OXCT1/PFKM/SCG2/SLC25A4/SOCS1/XBP1	11
BP	GO:0098869	cellular oxidant detoxification	0,00211675	0,03015353	HBA2/HBE1/HBZ/PRDX6/PTGS2	5
BP	GO:0046390	ribose phosphate biosynthetic process	0,00222503	0,03095037	ACTN3/AK1/ALDOA/FBP1/GPD1/PFKM/PGK1/PGM1	8
BP	GO:0010332	response to gamma radiation	0,00236849	0,03276196	BCL2L1/CRYAB/EGR1/MYC	4
BP	GO:0006979	response to oxidative stress	0,00262981	0,03538814	ADIPOQ/ARG1/CRYAB/DUSP1/EGFR/HBA2/MMP3/PON2/PRDX6/PTGS2/TPM1	11
BP	GO:0019221	cytokine-mediated signaling pathway	0,00270874	0,03625314	ADIPOQ/ARG1/BCL2L1/CD74/EGR1/IRF1/MMP3/MYC/PELI2/PTGS2/SOCS1/SQSTM1	12
BP	GO:0050679	positive regulation of epithelial cell proliferation	0,00281448	0,03682866	ARG1/EGFR/FGFBP1/MYC/SCG2/XBP1	6
BP	GO:2000242	negative regulation of reproductive process	0,00285586	0,03682866	CDKN1B/DUSP1/SERPINF1	3
BP	GO:0030308	negative regulation of cell growth	0,00310167	0,03859161	CDKN1B/CRYAB/FBP1/FHL1/MYL2/SEMA3F	6
BP	GO:0051085	chaperone cofactor-dependent protein refolding	0,00327372	0,0395401	CD74/DNAJB1/DNAJB5	3
BP	GO:0071480	cellular response to gamma radiation	0,00327372	0,0395401	BCL2L1/CRYAB/EGR1	3

BP	GO:0071549	cellular response to dexamethasone stimulus	0,00327372	0,0395401	ARG1/EGFR/SERPINF1	3
BP	GO:0009611	response to wounding	0,00358425	0,0423374	ARG1/CYR61/EGFR/HBE1/IRF1/LAMB2/PABPC4/PPL/SERPINE1/TFPI2/TPM1/XBP1	12
BP	GO:0071230	cellular response to amino acid stimulus	0,00361973	0,04247609	BCL2L1/EGFR/SOCS1/XBP1	4
BP	GO:0035690	cellular response to drug	0,0036656	0,04281139	ADIPOQ/ARG1/EGFR/EGR1/FBP1/MMP3/MYC/PTGS2/SERPINF1	9
BP	GO:0010677	negative regulation of cellular carbohydrate metabolic process	0,00372715	0,04332595	ACTN3/ADIPOQ/FBP1	3
BP	GO:0043200	response to amino acid	0,00392312	0,04476339	ARG1/BCL2L1/EGFR/SOCS1/XBP1	5
BP	GO:0035850	epithelial cell differentiation involved in kidney development	0,00421706	0,04709107	ACTA2/ADIPOQ/LAMB2	3
BP	GO:0071353	cellular response to interleukin-4	0,00421706	0,04709107	ARG1/KEAP1/XBP1	3
BP	GO:0010942	positive regulation of cell death	0,00440524	0,04847726	ADIPOQ/BCL2L1/CDKN1B/CYR61/DUSP1/EGR1/G0S2/GADD45B/HBA2/MMP3/MYC/PTGS2/SQSTM1	13
BP	GO:0000302	response to reactive oxygen species	0,00450203	0,04910586	ARG1/CRYAB/DUSP1/EGFR/HBA2/MMP3/TPM1	7
BP	GO:0051271	negative regulation of cellular component movement	0,00458053	0,0493104	ADIPOQ/CDKN1B/CITED2/DUSP1/SEMA3F/SERPINE1/SERPINF1/TPM1	8
BP	GO:0009746	response to hexose	0,00467243	0,04986611	ADIPOQ/EGR1/OXCT1/PTGS2/SERPINF1/XBP1	6
BP	GO:0034405	response to fluid shear stress	0,00474429	0,04998664	CITED2/PTGS2/TFPI2	3
CC	GO:0044449	contractile fiber part	1,2553E-18	2,95E-16	ACTA1/ACTA2/ACTC1/ALDOA/CRYAB/DES/MYBPC2/MYBPC3/MYH1/MYH7/MYL1/MYL2/MYL3/MYL4/MYOZ1/SQSTM1/TNNC1/TNNC2/TNNI1/TNNI2/TNNT1/TNNT3/TPM1/TPM3	24
CC	GO:0030017	sarcomere	3,6889E-18	4,3344E-16	ACTA1/ACTC1/ALDOA/CRYAB/DES/MYBPC2/MYBPC3/MYH1/MYH7/MYL1/MYL2/MYL3/MYL4/MYOZ1/SQSTM1/TNNC1/TNNC2/TNNI1/TNNI2/TNNT1/TNNT3/TPM1/TPM3	23
CC	GO:0043292	contractile fiber	6,1388E-18	4,8087E-16	ACTA1/ACTA2/ACTC1/ALDOA/CRYAB/DES/MYBPC2/MYBPC3/MYH1/MYH7/MYL1/MYL2/MYL3/MYL4/MYOZ1/SQSTM1/TNNC1/TNNC2/TNNI1/TNNI2/TNNT1/TNNT3/TPM1/TPM3	24
CC	GO:0030016	myofibril	3,3189E-17	1,9498E-15	ACTA1/ACTC1/ALDOA/CRYAB/DES/MYBPC2/MYBPC3/MYH1/MYH7/MYL1/MYL2/MYL3/MYL4/MYOZ1/SQSTM1/TNNC1/TNNC2/TNNI1/TNNI2/TNNT1/TNNT3/TPM1/TPM3	23
CC	GO:0005865	striated muscle thin filament	1,4689E-14	6,904E-13	ACTA1/MYBPC2/MYBPC3/TNNC1/TNNC2/TNNI1/TNNI2/TNNT1/TNNT3/TPM1/TPM3	11
CC	GO:0036379	myofilament	2,4501E-14	9,5964E-13	ACTA1/MYBPC2/MYBPC3/TNNC1/TNNC2/TNNI1/TNNI2/TNNT1/TNNT3/TPM1/TPM3	11
CC	GO:0015629	actin cytoskeleton	5,4157E-14	1,8181E-12	ACTA1/ACTA2/ACTC1/ACTN3/ALDOA/CRYAB/KEAP1/MYBPC2/MYBPC3/MYH1/MYH7/MYL1/MYL2/MYL3/MYL4/MYOZ1/PDLIM7/PGM1/SMTNL2/TNNC1/TNNC2/TNNI1/TNNI2/TNNT1/TNNT3/TPM1/TPM3	27
CC	GO:0031672	A band	6,4663E-09	1,8995E-07	ALDOA/CRYAB/MYBPC2/MYBPC3/MYH1/MYL2/MYL3/MYL4	8
CC	GO:0016459	myosin complex	1,5028E-07	3,924E-06	MYBPC2/MYBPC3/MYH1/MYH7/MYL1/MYL2/MYL3/MYL4	8
CC	GO:0005859	muscle myosin complex	1,8089E-06	4,2509E-05	MYBPC3/MYH1/MYH7/MYL1/MYL3	5
CC	GO:0016460	myosin II complex	6,1417E-06	0,00013121	MYBPC3/MYH1/MYH7/MYL1/MYL3	5
CC	GO:0031674	I band	1,0778E-05	0,00021106	ACTC1/ALDOA/CRYAB/DES/MYBPC2/MYBPC3/MYH7/MYL3/MYOZ1	9
CC	GO:0005884	actin filament	5,7275E-05	0,00100777	ACTA1/ACTC1/ACTN3/KEAP1/SMTNL2/TPM1/TPM3	7

CC	GO:0032432	actin filament bundle	6,0038E-05	0,00100777	ACTA1/CRYAB/MYH7/PDLIM7/TPM1/TPM3	6
CC	GO:0042641	actomyosin	8,3852E-05	0,00131368	ACTA1/ACTC1/MYH7/PDLIM7/TPM1/TPM3	6
CC	GO:0032982	myosin filament	0,00014254	0,00209359	MYBPC2/MYBPC3/MYH1/MYH7	4
CC	GO:0031430	M band	0,00021065	0,00291194	ALDOA/CRYAB/MYBPC2/MYBPC3	4
CC	GO:0001725	stress fiber	0,00036707	0,00454006	ACTA1/MYH7/PDLIM7/TPM1/TPM3	5
CC	GO:0097517	contractile actin filament bundle	0,00036707	0,00454006	ACTA1/MYH7/PDLIM7/TPM1/TPM3	5
CC	GO:0030018	Z disc	0,00177645	0,02048522	CRYAB/DES/MYBPC2/MYBPC3/MYH7/MYOZ1	6
CC	GO:0031012	extracellular matrix	0,00183059	0,02048522	ADIPOQ/CYR61/FCN1/LAMB2/MFAP5/MMP19/MMP3/SERPINE1/SERPINF1/TFPI2/UCMA	11
CC	GO:0097223	sperm part	0,00373016	0,03984492	ALDOA/DNAJB1/PFKM/SPA17/SQSTM1	5
MF	GO:0003779	actin binding	8,4542E-08	2,7053E-05	ACTN3/ALDOA/EGFR/MYBPC2/MYBPC3/MYH1/MYH7/MYL2/MYL3/MYL4/MYOZ1/TNNC1/TNNC2/TNNI1/TNNI2/TNNT3/TPM1/TPM3	18
MF	GO:0008307	structural constituent of muscle	5,3365E-07	8,5384E-05	ACTN3/MYBPC2/MYBPC3/MYL1/MYL2/MYL3/TPM1	7
MF	GO:0017022	myosin binding	1,1378E-06	0,00012136	ACTA1/ACTC1/LARP6/MYBPC3/MYL2/MYL3/MYL4	7
MF	GO:0051015	actin filament binding	2,5028E-05	0,00200224	EGFR/MYBPC2/MYBPC3/MYH1/MYH7/MYL4/TNNC1/TNNC2/TPM1/TPM3	10
MF	GO:0004601	peroxidase activity	0,00019621	0,01255745	HBA2/HBE1/HBZ/PRDX6/PTGS2	5
MF	GO:0016684	oxidoreductase activity, acting on peroxide as acceptor	0,0003137	0,01673062	HBA2/HBE1/HBZ/PRDX6/PTGS2	5
MF	GO:0003785	actin monomer binding	0,00108201	0,04946311	MYL2/MYL3/MYL4	3

Supplementary Table 10: Gene Ontology enrichment of the negatively-correlated, common genes between regenerative vs. wound healing and wound healing vs. control comparisons from DE lists of Microarray and RNA-Seq

A. GO enrichment of the negatively-correlated genes from Microarray DE list

ONTOLOGY	ID	Description	GeneRatio	BgRatio	pvalue	p.adjust	qvalue	geneID	Count
BP	GO:0006888	ER to Golgi vesicle-mediated transport	24/520	211/18493	6,7E-09	2,88E-05	2,54E-05	CUL3/ERGIC3/COG4/COPB1/SEC24C/TRAPPC2/KDELR2/SCFD1/DCTN6/TEX261/SPAST/GAS6/SPTAN1/TFG/RANGRF/BCAP31/TRAPPC2L/YIF1A/SEC31A/ZW10/KDELR3/DCTN2/KLHL12/STX5	24
BP	GO:0034470	ncRNA processing	32/520	376/18493	2,87E-08	6,17E-05	5,45E-05	OSGEP/ELP2/INTS8/THUMPD1/AARS/MRPS9/RPSA/NSUN2/LAGE3/REXO4/EXOSC10/TRMT12/RPP40/XRN2/LCMT2/NOL6/KRI1/EXOSC1/C1D/DUS1L/RCL1/TRMT1/TSEN2/RNF113A/NOL10/NSUN5/SEPHS2/CDKAL1/SARS/POP4/NOP2/RIOK3	32
BP	GO:0140053	mitochondrial gene expression	18/520	159/18493	5,59E-07	0,000481	0,000425	MRPS14/MRPL27/LRPPRC/SUPV3L1/TFAM/MRPS27/DAP3/MRPL24/MRPS9/MRPS34/GARS/HARS/SHMT2/ERAL1/MRPS18A/TUFM/MRPS10/CHCHD1	18
BP	GO:0032543	mitochondrial translation	16/520	135/18493	1,28E-06	0,00092	0,000812	MRPS14/MRPL27/LRPPRC/MRPS27/DAP3/MRPL24/MRPS9/MRPS34/GARS/HARS/SHMT2/ERAL1/MRPS18A/TUFM/MRPS10/CHCHD1	16
BP	GO:0015671	oxygen transport	6/520	15/18493	1,94E-06	0,001189	0,00105	MYC/HBA2/HBZ/HBG1/HBD/HBE1	6
BP	GO:0035967	cellular response to topologically incorrect protein	16/520	151/18493	5,64E-06	0,002706	0,002389	CUL3/CXXC1/COPS5/TBL2/YIF1A/ADD1/SEC31A/KDELR3/ANKZF1/HSPA1L/HSPA9/DDIT3/AGR2/ATF4/DNAJB9/HERPUD1	16
BP	GO:0035966	response to topologically incorrect protein	18/520	189/18493	6,8E-06	0,002706	0,002389	CUL3/CXXC1/COPS5/TBL2/YIF1A/STT3B/ADD1/SEC31A/KDELR3/ANKZF1/HSPA1L/HSPA9/DDIT3/AGR2/ATF4/DNAJB9/DNAJB1/HERPUD1	18
BP	GO:0070203	regulation of establishment of protein localization to telomere	5/520	11/18493	6,92E-06	0,002706	0,002389	CCT4/CCT8/CCT6A/TERF1/CCT5	5
BP	GO:0051052	regulation of DNA metabolic process	28/520	412/18493	1,69E-05	0,003895	0,003439	CCT4/SMC3/TRIP12/UBR5/TRIM28/DFFA/APAF1/THOC1/CCT8/EXOSC10/PRDM9/UBE2V2/SMARCAD1/CCT6A/CDC6/DEK/TERF1/XRCC5/HUS1/GMNN/CCT5/ZNF830/CEBPG/FGFR1/TGFB1/MYC/DUSP1/EGFR	28
BP	GO:2000058	regulation of ubiquitin-dependent protein catabolic process	15/520	147/18493	1,74E-05	0,003895	0,003439	OGT/WAC/USP5/UCHL5/UBE2V2/CCDC22/HSPBP1/BCAP31/TRIM39/PTK2/RNF19B/PLK3/RNF14/HERPUD1/KEAP1	15
BP	GO:0046148	pigment biosynthetic process	9/520	54/18493	1,77E-05	0,003895	0,003439	FXN/AP3D1/SHMT1/DDT/HMBS/PRPS1/UROD/FECH/ALAS2	9
BP	GO:0034976	response to endoplasmic reticulum stress	21/520	264/18493	1,97E-05	0,003895	0,003439	CXXC1/APAF1/COPS5/RNF103/TBL2/TMX1/BCAP31/TXNDC12/YIF1A/STT3B/ADD1/SEC31A/KDELR3/P4HB/ANKZF1/DDIT3/SRPX/AGR2/ATF4/DNAJB9/HERPUD1	21
BP	GO:0022613	ribonucleoprotein complex biogenesis	31/520	485/18493	2,02E-05	0,003895	0,003439	GEMIN7/PRPF8/SNRNP200/PAN2/LSM14A/THUMPD1/TGS1/MRPS9/RPSA/REXO4/EXOSC10/DENR/EIF3D/EIF3A/SNUPN/XRN2/ERAL1/NOL6/KRI1/EXOSC1/C1D/RCL1/NOL10/NSUN5/CD2BP2/POP4/EIF3L/NOP2/SRPK3/DDX3X/RIOK3	31
BP	GO:0042440	pigment metabolic process	10/520	69/18493	2,17E-05	0,003895	0,003439	FXN/AP3D1/SHMT1/DDT/HMBS/PRPS1/UROD/BLVRB/FECH/ALAS2	10

BP	GO:0033044	regulation of chromosome organization	24/520	330/18493	2,28E-05	0,003895	0,003439	OGT/RAD21/CCT4/CUL3/TRIP12/UBR5/TRIM28/CCT8/EXOSC10/MCPH1/CCT6A/CDC6/TERF1/XRCC5/CDK5RAP2/CCT5/TAF7/LCMT1/CTR9/ZW10/TGFB1/TAL1/MYC/DUSP1	24
BP	GO:0045862	positive regulation of proteolysis	25/520	352/18493	2,3E-05	0,003895	0,003439	OGT/PSMD14/APAF1/USP5/UBE2V2/TFAP4/CCDC22/HSPBP1/BID/BCAP31/PDCD5/PRELID1/PHB/PTK2/RNF19B/BCL2L10/CTGF/PLK3/DDX3X/ASTL/MYC/RNF14/HERPUD1/CYR61/KEAP1	25
BP	GO:0035722	interleukin-12-mediated signaling pathway	8/520	47/18493	4,43E-05	0,006149	0,005429	MTAP/RALA/SOD1/TYK2/P4HB/RAP1B/HSPA9/GSTO1	8
BP	GO:0070200	establishment of protein localization to telomere	5/520	16/18493	5,82E-05	0,007588	0,0067	CCT4/CCT8/CCT6A/TERF1/CCT5	5
BP	GO:0071349	cellular response to interleukin-12	8/520	49/18493	6,05E-05	0,007653	0,006758	MTAP/RALA/SOD1/TYK2/P4HB/RAP1B/HSPA9/GSTO1	8
BP	GO:0070671	response to interleukin-12	8/520	50/18493	7,03E-05	0,008396	0,007413	MTAP/RALA/SOD1/TYK2/P4HB/RAP1B/HSPA9/GSTO1	8
BP	GO:0006913	nucleocytoplasmic transport	24/520	357/18493	7,96E-05	0,009203	0,008126	GLE1/GEMIN7/BARD1/UBR5/TRIM28/RPSA/PHAX/RANGAP1/THOC1/YWHAE/THOC2/SNUPN/NOL6/GAS6/CPSF2/PHB2/HTATIP2/SRRM1/PPP3CA/DNAJC27/CDK5/TGFB1/PTGS2/NFKBIA	24
BP	GO:0070646	protein modification by small protein removal	21/520	292/18493	8,58E-05	0,009203	0,008126	OGT/PSMD1/BARD1/POLB/PSMD9/PSMD14/USP5/ACTR8/MAP3K7/SHMT2/UCHL5/COPS5/OTUD5/COPS4/BIRC3/WDR20/ADRB2/MYC/KEAP1/NFKBIA/USP2	21
BP	GO:0051169	nuclear transport	24/520	360/18493	9,06E-05	0,009203	0,008126	GLE1/GEMIN7/BARD1/UBR5/TRIM28/RPSA/PHAX/RANGAP1/THOC1/YWHAE/THOC2/SNUPN/NOL6/GAS6/CPSF2/PHB2/HTATIP2/SRRM1/PPP3CA/DNAJC27/CDK5/TGFB1/PTGS2/NFKBIA	24
BP	GO:0043687	post-translational protein modification	24/520	361/18493	9,45E-05	0,009203	0,008126	PSMD1/CUL3/RAB2A/DCAF10/SUMF1/UBA3/PSMD9/PSMD14/RABGGTB/NAE1/GAS6/COPS5/WIPI2/FBXL15/CCDC22/DCAF4/LCMT1/STT3B/COPS4/P4HB/LMO7/WSB1/CYR61/KEAP1	24
BP	GO:0043624	cellular protein complex disassembly	17/520	210/18493	9,68E-05	0,009203	0,008126	GLE1/MRPS14/MRPL27/MRPS27/DAP3/MRPL24/MRPS9/MRPS34/ERAL1/SPAST/MRPS18A/SPTAN1/MRPS10/ADD1/CHCHD1/PLEK/SPEF1	17
BP	GO:0048208	COPII vesicle coating	9/520	67/18493	0,000103	0,009203	0,008126	CUL3/SEC24C/TRAPPC2/SCFD1/TFG/TRAPPC2L/SEC31A/KLHL12/STX5	9
BP	GO:0030968	endoplasmic reticulum unfolded protein response	12/520	116/18493	0,000104	0,009203	0,008126	CXXC1/COPS5/TBL2/YIF1A/ADD1/SEC31A/KDELR3/DDIT3/AGR2/ATF4/DNAJB9/HERPUD1	12
BP	GO:0033157	regulation of intracellular protein transport	18/520	232/18493	0,000105	0,009203	0,008126	BARD1/UBR5/TRIM28/RANGAP1/YWHAE/MFF/GAS6/PARL/HAX1/BCAP31/PDCD5/ITGB1BP1/DNAJC27/HSPA1L/CDK5/TGFB1/PLK3/PTGS2	18
BP	GO:1904872	regulation of telomerase RNA localization to Cajal body	5/520	18/18493	0,000109	0,009203	0,008126	CCT4/CCT8/EXOSC10/CCT6A/CCT5	5
BP	GO:0033314	mitotic DNA replication checkpoint	4/520	10/18493	0,000113	0,009203	0,008126	NAE1/CDC6/HUS1/ZNF830	4
BP	GO:1903405	protein localization to nuclear body	4/520	10/18493	0,000113	0,009203	0,008126	CCT4/CCT8/CCT6A/CCT5	4
BP	GO:1904867	protein localization to Cajal body	4/520	10/18493	0,000113	0,009203	0,008126	CCT4/CCT8/CCT6A/CCT5	4

BP	GO:0016579	protein deubiquitination	20/520	276/18493	0,000113	0,009203	0,008126	OGT/PSMD1/BARD1/POLB/PSMD9/PSMD14/USP5/ACTR8/MAP3K7/SHMT2/UCHL5/COPS5/OTUD5/BIRC3/WDR20/ADRB2/MYC/KEAP1/NFKBIA/USP2	20
BP	GO:0006900	vesicle budding from membrane	11/520	101/18493	0,000127	0,009901	0,008742	CUL3/SEC24C/TRAPPC2/SCFD1/AP3D1/AP2M1/TFG/TRAPPC2L/SEC31A/KLHL12/STX5	11
BP	GO:1905477	positive regulation of protein localization to membrane	12/520	119/18493	0,000133	0,010026	0,008852	YWHAE/MFF/PTPN9/RANGRF/BID/PDCD5/ITGB1BP1/CDK5/AGR2/TGFB1/EGFR/SQSTM1	12
BP	GO:0006520	cellular amino acid metabolic process	22/520	324/18493	0,000136	0,010026	0,008852	NIT2/AARS/ALDH18A1/CARS/MTAP/FPGS/ENOPH1/GARS/HARS/HIBCH/SHMT2/SHMT1/MTR/MCCC2/BCKDHA/PYCR1/SEPHS2/P4HB/SARS/HAL/ATF4/MSRA	22
BP	GO:0090685	RNA localization to nucleus	5/520	19/18493	0,000145	0,010026	0,008852	CCT4/CCT8/EXOSC10/CCT6A/CCT5	5
BP	GO:0010458	exit from mitosis	6/520	30/18493	0,00016	0,010564	0,009327	SPAST/ANLN/PHB2/SIRT7/ZW10/TGFB1	6
BP	GO:0042168	heme metabolic process	6/520	30/18493	0,00016	0,010564	0,009327	FXN/HMBS/UROD/BLVRB/FECH/ALAS2	6
BP	GO:0042743	hydrogen peroxide metabolic process	8/520	57/18493	0,000182	0,011188	0,009878	SOD1/APOA4/HBA2/HBZ/HBG1/HBD/HBE1/EGFR	8
BP	GO:0051302	regulation of cell division	14/520	162/18493	0,000199	0,012079	0,010665	CUL3/DLL1/PIN1/SPAST/CALM1/CDC6/RBL1/INTU/ITGB1BP1/RAB11FIP4/TGFB1/PLK3/TAL1/MYC	14
BP	GO:0042744	hydrogen peroxide catabolic process	6/520	32/18493	0,000233	0,013602	0,01201	APOA4/HBA2/HBZ/HBG1/HBD/HBE1	6
BP	GO:0006783	heme biosynthetic process	5/520	21/18493	0,000242	0,013845	0,012224	FXN/HMBS/UROD/FECH/ALAS2	5
BP	GO:0071897	DNA biosynthetic process	15/520	189/18493	0,000304	0,015935	0,014069	CCT4/POLB/TYMS/CCT8/EXOSC10/CCT6A/TERF1/XRCC5/CCT5/ZBTB1/RFC1/PHB/CTGF/MYC/DUSP1	15
BP	GO:2001235	positive regulation of apoptotic signaling pathway	14/520	170/18493	0,000329	0,016874	0,014899	CASP2/APAF1/YWHAE/MFF/TFAP4/DNM1L/SOD1/BID/BCAP31/TRIM39/PDCD5/DDIT3/SRPX/G0S2	14
BP	GO:0010948	negative regulation of cell cycle process	20/520	304/18493	0,000406	0,020059	0,017711	RAD21/SMC3/ZNF268/CASP2/APAF1/NAE1/TFAP4/CDC6/TERF1/HUS1/CDK5RAP2/LCMT1/RBL1/ZNF830/ZW10/BMP7/PLK3/ATF5/CDKN1B/DUSP1	20
BP	GO:1901990	regulation of mitotic cell cycle phase transition	23/520	375/18493	0,000419	0,020314	0,017936	RAD21/CUL3/CASP2/YWHAE/CEP70/NAE1/ANLN/TFAP4/CDC6/HUS1/CDK5RAP2/PHB2/BID/LCMT1/RBL1/ZNF830/ZW10/DCTN2/TGFB1/PLK3/DDX3X/CDKN1B/DUSP1	23
BP	GO:0000075	cell cycle checkpoint	16/520	216/18493	0,000421	0,020314	0,017936	WAC/CASP2/NSUN2/THOC1/NAE1/CDC6/HUS1/CDK5RAP2/ATRIP/LCMT1/ZNF830/ZW10/TGFB1/PLK3/CDKN1B/DUSP1	16
BP	GO:0006457	protein folding	16/520	218/18493	0,000466	0,021992	0,019418	CCT4/NUDC/DFFA/PPIE/CCT8/PPIB/CCT6A/CCT5/HSPBP1/DNAJA2/PDCD5/P4HB/HSPA1L/HSPA9/APCS/DNAJB1	16
BP	GO:0007088	regulation of mitotic nuclear division	14/520	177/18493	0,000495	0,022745	0,020083	RAD21/SMC3/CUL3/PIN1/CCNE2/ANLN/CDC6/CDK5RAP2/PHB2/LCMT1/ZW10/BMP7/TGFB1/DUSP1	14
BP	GO:0032259	methylation	22/520	358/18493	0,000541	0,0235	0,020749	OGT/CXXC1/TRIM28/ZCCHC4/N6AMT1/TGS1/METTL5/NSUN2/DPY30/TYMS/PRDM9/TRMT12/LCMT2/MTR/TRMT1/LCMT1/CTR9/NSUN5/NOP2/GSTO1/FOS/MYC	22
BP	GO:0098869	cellular oxidant detoxification	10/520	101/18493	0,000552	0,023504	0,020753	SOD1/BMP7/GSTO1/PTGS2/APOA4/HBA2/HBZ/HBG1/HBD/HBE1	10

BP	GO:0045454	cell redox homeostasis	8/520	67/18493	0,000563	0,023504	0,020753	TXNL1/GLRX3/TMX1/GLRX2/TXNDC12/SCO2/P4HB/DDIT3	8
BP	GO:0006730	one-carbon metabolic process	5/520	25/18493	0,000575	0,023504	0,020753	TYMS/FPGS/SHMT2/SHMT1/MAT2A	5
BP	GO:0007339	binding of sperm to zona pellucida	6/520	38/18493	0,000617	0,024093	0,021273	CCT4/CCT8/VDAC2/CCT5/HSPA1L/ASTL	6
BP	GO:0006790	sulfur compound metabolic process	22/520	362/18493	0,000628	0,024093	0,021273	FXN/NUBPL/GLRX3/MTAP/THTPA/ENOPH1/PCCA/MTR/MCCC2/GLRX2/BPNT1/SOD1/ACSM3/CSGALNACT2/HSPA9/GSTO1/ELOVL7/B4GALT4/SLC35D1/MAT2A/FAR1/MSRA	22
BP	GO:0043249	erythrocyte maturation	4/520	15/18493	0,000659	0,024664	0,021777	MAEA/KLF2/TAL1/HBZ	4
BP	GO:0070371	ERK1 and ERK2 cascade	21/520	341/18493	0,000699	0,025451	0,022472	PIN1/EIF3A/RYK/GAS6/RANBP9/NPTN/PHB2/DDT/SPRY1/ITGB1BP1/PHB/OXTR/DNAJC27/RAP1B/FGFR1/TGFB1/CTGF/MYC/DUSP1/EGFR/CYR61	21
BP	GO:0001558	regulation of cell growth	24/520	416/18493	0,000756	0,026224	0,023155	FXN/SUPV3L1/N6AMT1/ADNP2/CDKN2C/PIN1/RYK/EAF2/EBAG9/TMEM97/PHB/ITSN2/SH3BP4/IP6K2/CDK5/TGFB1/CTGF/DDX3X/CDKN1B/SEMA3F/EGFR/SGK1/CYR61/FBP1	24
BP	GO:0043281	regulation of cysteine-type endopeptidase activity involved in apoptotic process	15/520	209/18493	0,000872	0,029064	0,025662	APAF1/YWHAE/GAS6/TFAP4/BID/BCAP31/PDCD5/BIRC3/BCL2L10/CTGF/DDX3X/PTGS2/MYC/HERPUD1/CYR61	15
BP	GO:0097237	cellular response to toxic substance	16/520	235/18493	0,001045	0,032806	0,028966	FXN/SIGMAR1/SOD1/PYCR1/ANKZF1/KLF2/BMP7/GSTO1/PTGS2/EGR1/APOA4/HBA2/HBZ/HBG1/HBD/HBE1	16
BP	GO:0001890	placenta development	12/520	149/18493	0,001045	0,032806	0,028966	TRIM28/OVOL2/GGNBP2/SOD1/NR2F2/BMP7/ST14/PTK2/PTGS2/CDKN1B/EGFR/CYR61	12
BP	GO:0032388	positive regulation of intracellular transport	15/520	214/18493	0,001108	0,033786	0,029831	UBR5/TRIM28/YWHAE/MFF/GAS6/DNM1L/BCAP31/PDCD5/SCP2/ITGB1BP1/HSPA1L/CDK5/TGFB1/PLK3/PTGS2	15
BP	GO:0045926	negative regulation of growth	17/520	260/18493	0,001152	0,034678	0,030619	FXN/CDKN2C/TBX5/RYK/EAF2/PHB/SH3BP4/IP6K2/CDK5/PTK2/TGFB1/DDX3X/ADRB2/CDKN1B/SEMA3F/SQSTM1/FBP1	17
BP	GO:0045912	negative regulation of carbohydrate metabolic process	6/520	43/18493	0,00121	0,035793	0,031603	COX11/MAEA/PLEK/TGFB1/SIK1/FBP1	6
BP	GO:0046822	regulation of nucleocytoplasmic transport	10/520	112/18493	0,001236	0,036088	0,031864	BARD1/UBR5/TRIM28/RANGAP1/YWHAE/GAS6/DNAJC27/CDK5/TGFB1/PTGS2	10
BP	GO:0009896	positive regulation of catabolic process	23/520	407/18493	0,001271	0,036678	0,032385	BARD1/SUPV3L1/WAC/IDE/USP5/MAP3K7/EPM2A/UBE2V2/CCDC22/HSPBP1/BCAP31/SH3BP4/PTK2/MOV10/RNF19B/PLK3/ADRB2/APOA4/CDKN1B/RNF14/HERPUD1/KEAP1/STX5	23
BP	GO:0097193	intrinsic apoptotic signaling pathway	18/520	287/18493	0,001339	0,038133	0,03367	CUL3/POLB/CASP2/APAF1/VDAC2/DNM1L/NUPR1/SOD1/BID/BCAP31/TXNDC12/P4HB/DDIT3/BCL2L10/ATF4/DDX3X/PTGS2/HERPUD1	18
BP	GO:0009130	pyrimidine nucleoside monophosphate biosynthetic process	4/520	18/18493	0,001382	0,038819	0,034275	TYMS/UPRT/UMPS/SHMT1	4
BP	GO:0002753	cytoplasmic pattern recognition receptor signaling pathway	7/520	60/18493	0,001421	0,039152	0,034569	LSM14A/MAP3K7/TSPAN6/BIRC3/TIFA/RIOK3/NFKBIA	7
BP	GO:0051306	mitotic sister chromatid separation	7/520	60/18493	0,001421	0,039152	0,034569	RAD21/CUL3/CDC6/CDK5RAP2/LCMT1/ZW10/DUSP1	7
BP	GO:1905475	regulation of protein localization to membrane	13/520	177/18493	0,001532	0,041548	0,036685	YWHAE/MFF/PTPN9/AP2M1/RANGRF/BID/PDCD5/ITGB1BP1/CDK5/AGR2/TGFB1/EGFR/SQSTM1	13
BP	GO:1905818	regulation of chromosome separation	7/520	62/18493	0,001724	0,044652	0,039426	RAD21/CUL3/CDC6/CDK5RAP2/LCMT1/ZW10/DUSP1	7

BP	GO:0000302	response to reactive oxygen species	15/520	224/18493	0,001744	0,044889	0,039635	FXN/SIGMAR1/MTR/GLRX2/SOD1/PYCR1/ANKZF1/KLF2/BMP7/FOS/PLK3/APOA4/HBA2/DUSP1/EGFR	15
CC	GO:0005759	mitochondrial matrix	41/531	462/19659	2,57E-11	1,28E-08	1,07E-08	MRPS14/MRPL27/LRPPRC/FXN/SUPV3L1/TFAM/MRPS27/DAP3/NUBPL/MRPL24/MIPEP/MRPS9/TYMS/FPGS/MRPS34/PMPCB/ABHD10/GARS/PCCA/HIBCH/SHMT2/ERAL1/VDAC2/FDXR/MRPS18A/TUFM/MCCC2/BCKDHA/GLRX2/ETFB/IDH3B/SOD1/MRPS10/MPG/PYCR1/SCO2/ACSM3/CHCHD1/HSPA9/FECH/ALAS2	41
CC	GO:0031838	haptoglobin-hemoglobin complex	5/531	11/19659	5,7E-06	0,000911	0,000758	HBA2/HBZ/HBG1/HBD/HBE1	5
CC	GO:0000314	organellar small ribosomal subunit	7/531	28/19659	7,29E-06	0,000911	0,000758	MRPS14/MRPS27/DAP3/MRPS9/MRPS34/MRPS18A/MRPS10	7
CC	GO:0005763	mitochondrial small ribosomal subunit	7/531	28/19659	7,29E-06	0,000911	0,000758	MRPS14/MRPS27/DAP3/MRPS9/MRPS34/MRPS18A/MRPS10	7
CC	GO:0005833	hemoglobin complex	5/531	12/19659	9,55E-06	0,000955	0,000794	HBA2/HBZ/HBG1/HBD/HBE1	5
CC	GO:0016607	nuclear speck	26/531	382/19659	1,68E-05	0,001059	0,000881	OSGEP/PRPF8/BARD1/WAC/CXXC1/TRIP12/REXO4/PPIE/THOC1/BCAS2/VPS72/PIN1/THOC2/NCAPG2/EAF2/CWC22/SRRM1/ZC3H14/CTR9/COPS4/CD2BP2/ZNF830/ATPAF2/DDX3X/CCNL1/SGK1	26
CC	GO:0009295	nucleoid	8/531	43/19659	1,69E-05	0,001059	0,000881	LRPPRC/SUPV3L1/TFAM/SHMT2/VDAC2/TUFM/MPG/HSPA9	8
CC	GO:0042645	mitochondrial nucleoid	8/531	43/19659	1,69E-05	0,001059	0,000881	LRPPRC/SUPV3L1/TFAM/SHMT2/VDAC2/TUFM/MPG/HSPA9	8
CC	GO:0005791	rough endoplasmic reticulum	11/531	86/19659	1,98E-05	0,001097	0,000913	RP9/STAU1/RPN2/FKRP/RANGRF/BCAP31/PTGDS/CDKAL1/TMEM97/SEC61A1/SFTPD	11
CC	GO:0015935	small ribosomal subunit	10/531	73/19659	2,56E-05	0,001281	0,001066	MRPS14/MRPS27/DAP3/MRPS9/RPSA/MRPS34/MRPS18A/MRPS10/DDX3X/HBA2	10
CC	GO:0031968	organelle outer membrane	17/531	204/19659	4,15E-05	0,001852	0,00154	LRPPRC/SIGMAR1/RTN4IP1/MFF/VDAC2/DNM1L/PHB2/HAX1/BID/WASF1/NUCB2/PHB/ANKZF1/CNP/BCL2L10/DDX3X/RAB11FIP5	17
CC	GO:0019867	outer membrane	17/531	206/19659	4,69E-05	0,001852	0,00154	LRPPRC/SIGMAR1/RTN4IP1/MFF/VDAC2/DNM1L/PHB2/HAX1/BID/WASF1/NUCB2/PHB/ANKZF1/CNP/BCL2L10/DDX3X/RAB11FIP5	17
CC	GO:0005852	eukaryotic translation initiation factor 3 complex	5/531	16/19659	4,81E-05	0,001852	0,00154	EIF3D/EIF3A/COPS5/EIF3L/DDX3X	5
CC	GO:0044445	cytosolic part	19/531	250/19659	5,31E-05	0,001897	0,001578	PSMD1/CCT4/FXN/IDE/RPSA/PSMD14/APAF1/CCT8/PIN1/MAP3K7/UCHL5/CCT6A/CCT5/DDX3X/HBA2/HBZ/HBG1/HBD/HBE1	19
CC	GO:0005743	mitochondrial inner membrane	28/531	463/19659	6,69E-05	0,00223	0,001854	MRPS14/MRPL27/MRPS27/COX11/DAP3/MRPL24/MRPS9/TYMS/ALDH18A1/FPGS/MRPS34/PMPCB/SHMT2/ERAL1/FDXR/MRPS18A/PARL/ECSIT/PHB2/MRPS10/SCO2/ACAD11/PHB/CHCHD1/CNP/COQ10B/FECH/ALAS2	28
CC	GO:0000313	organellar ribosome	10/531	87/19659	0,000118	0,003465	0,002881	MRPS14/MRPL27/MRPS27/DAP3/MRPL24/MRPS9/MRPS34/MRPS18A/MRPS10/CHCHD1	10
CC	GO:0005761	mitochondrial ribosome	10/531	87/19659	0,000118	0,003465	0,002881	MRPS14/MRPL27/MRPS27/DAP3/MRPL24/MRPS9/MRPS34/MRPS18A/MRPS10/CHCHD1	10
CC	GO:0005832	chaperonin-containing T-complex	4/531	11/19659	0,000149	0,004112	0,003419	CCT4/CCT8/CCT6A/CCT5	4
CC	GO:0101031	chaperone complex	5/531	20/19659	0,000156	0,004112	0,003419	CCT4/CCT8/STIP1/CCT6A/CCT5	5

CC	GO:0005741	mitochondrial outer membrane	14/531	180/19659	0,000395	0,009874	0,008211	RTN4IP1/MFF/VDAC2/DNM1L/PHB2/HAX1/BID/WASF1/PHB/ANKZF1/CNP/BCL2L10/DDX3X/RAB11FIP5	14
CC	GO:0016282	eukaryotic 43S preinitiation complex	4/531	17/19659	0,000947	0,022538	0,018742	EIF3D/EIF3A/EIF3L/EIF1B	4
CC	GO:0070993	translation preinitiation complex	4/531	18/19659	0,001191	0,027074	0,022514	EIF3D/EIF3A/EIF3L/EIF1B	4
MF	GO:0031720	haptoglobin binding	5/525	10/17632	5,11E-06	0,002512	0,002262	HBA2/HBZ/HBG1/HBD/HBE1	5
MF	GO:0016667	oxidoreductase activity, acting on a sulfur group of donors	10/525	58/17632	7,26E-06	0,002512	0,002262	SUMF1/TXNL1/GLRX3/TMX1/GLRX2/TXNDC12/SCO2/P4HB/GSTO1/MSRA	10
MF	GO:0140101	catalytic activity, acting on a tRNA	14/525	120/17632	1,34E-05	0,002941	0,002648	AARS/NSUN2/CARS/TRMT12/RPP40/GARS/HARS/LCMT2/DUS1L/TRMT1/TSEN2/CDKAL1/SARS/POP4	14
MF	GO:0015036	disulfide oxidoreductase activity	8/525	40/17632	1,94E-05	0,002941	0,002648	TXNL1/GLRX3/TMX1/GLRX2/TXNDC12/SCO2/P4HB/GSTO1	8
MF	GO:0140098	catalytic activity, acting on RNA	26/525	352/17632	2,12E-05	0,002941	0,002648	PRPF8/SNRNP200/SUPV3L1/PAN2/DHX57/TGS1/AARS/NSUN2/CARS/EXOSC10/TRMT12/RPP40/GARS/HARS/XRN2/LCMT2/DUS1L/RCL1/TRMT1/TSEN2/CDKAL1/SARS/POP4/NOP2/MOV10/DDX3X	26
MF	GO:0140104	molecular carrier activity	8/525	43/17632	3,39E-05	0,003637	0,003275	FXN/SNUPN/SOD1/HBA2/HBZ/HBG1/HBD/HBE1	8
MF	GO:0005344	oxygen carrier activity	5/525	14/17632	3,68E-05	0,003637	0,003275	HBA2/HBZ/HBG1/HBD/HBE1	5
MF	GO:0015037	peptide disulfide oxidoreductase activity	5/525	15/17632	5,38E-05	0,004656	0,004193	GLRX3/GLRX2/TXNDC12/P4HB/GSTO1	5
MF	GO:0016741	transferase activity, transferring one-carbon groups	18/525	228/17632	0,000171	0,013126	0,011821	CXXC1/ZCCHC4/N6AMT1/TGS1/METTL5/NSUN2/DPY30/TYMS/PRDM9/TRMT12/LCMT2/SHMT2/SHMT1/MTR/TRMT1/LCMT1/NSUN5/NOP2	18
MF	GO:0051082	unfolded protein binding	12/525	127/17632	0,000413	0,028555	0,025715	CCT4/NUDC/PPIE/CCT8/PPIB/CCT6A/CCT5/DNAJA2/HSPA1L/HSPA9/APCS/DNAJB1	12
MF	GO:0003743	translation initiation factor activity	7/525	51/17632	0,000742	0,044664	0,040221	EIF2B4/DENR/EIF3D/EIF3A/COPS5/EIF3L/EIF1B	7
MF	GO:0008168	methyltransferase activity	16/525	216/17632	0,000775	0,044664	0,040221	CXXC1/ZCCHC4/N6AMT1/TGS1/METTL5/NSUN2/DPY30/TYMS/PRDM9/TRMT12/LCMT2/MTR/TRMT1/LCMT1/NSUN5/NOP2	16
MF	GO:0048037	cofactor binding	28/525	495/17632	0,000936	0,044664	0,040221	FXN/NUBPL/CYP20A1/GLRX3/UXS1/TYMS/GPHN/PCCA/SHMT2/SHMT1/MTR/DUS1L/GLRX2/IDH3B/SIRT7/SCP2/CDKAL1/ACAD11/CYP2C8/COQ10B/FECH/PTGS2/HBA2/HBZ/HBG1/HBD/HBE1/ALAS2	28
MF	GO:0016830	carbon-carbon lyase activity	7/525	53/17632	0,000938	0,044664	0,040221	UXS1/UMPS/SHMT2/SHMT1/BCKDHA/DDT/UROD	7
MF	GO:0031625	ubiquitin protein ligase binding	19/525	286/17632	0,000982	0,044664	0,040221	PSMD1/LRPPRC/CUL3/TRIM28/RANGAP1/YWHAE/RALA/NAE1/XRCC5/DNM1L/HSPBP1/BID/UBE2T/HSPA1L/HSPA9/EGFR/SQSTM1/NFKBIA/USP2	19
MF	GO:0016209	antioxidant activity	9/525	86/17632	0,001033	0,044664	0,040221	SOD1/GSTO1/PTGS2/APOA4/HBA2/HBZ/HBG1/HBD/HBE1	9
MF	GO:0043177	organic acid binding	15/525	204/17632	0,001205	0,049044	0,044165	AARS/TYMS/PCCA/SHMT2/UBR2/SHMT1/NR2F2/SCP2/PTGDS/HBA2/HBZ/HBG1/HBD/HBE1/ALAS2	15

B. GO enrichment of the negatively-correlated genes from RNA-Seq DE list

ONTOLOGY	ID	Description	GeneRatio	BgRatio	pvalue	p.adjust	qvalue	geneID	Count
BP	GO:0032963	collagen metabolic process	7/153	106/18493	2,84E-05	0,025757	0,022684	ID1/MMP1/MMP3/ARG1/LARP6/PRTN3/HIF1A	7
BP	GO:0034123	positive regulation of toll-like receptor signaling pathway	4/153	22/18493	2,93E-05	0,025757	0,022684	F2RL1/PJA2/TLR5/PELI1	4
BP	GO:0051384	response to glucocorticoid	8/153	149/18493	3,4E-05	0,025757	0,022684	ZFP36/PTGS2/AREG/FOXO1/ARG1/DUSP1/DDIT4/FOS	8

BP	GO:0035690	cellular response to drug	12/153	355/18493	4,07E-05	0,025757	0,022684	ID1/PTGS2/FOXO1/MMP3/FBP1/PDE4B/EGR1/ARG1/DDIT4/RHOB/ACTB/RNF149	12
BP	GO:1901652	response to peptide	14/153	481/18493	4,75E-05	0,025757	0,022684	ID1/JUND/PTGS2/AREG/TIMP1/FOXO1/SLC2A1/NR4A1/SOCS1/EGR1/KHK/ARG1/KLF10/GAB1	14
BP	GO:0071560	cellular response to transforming growth factor beta stimulus	9/153	242/18493	0,000189	0,045132	0,039747	ITGA3/ID1/ACVR1/ZYX/ARG1/FOS/CDKN1C/KLF10/UBC	9
BP	GO:0010038	response to metal ion	11/153	358/18493	0,000198	0,045132	0,039747	HVCN1/JUND/PTGS2/SLC30A1/FBP1/CDKN1B/KHK/ARG1/DUSP1/FOS/HIF1A	11
BP	GO:0007179	transforming growth factor beta receptor signaling pathway	8/153	193/18493	0,000207	0,045132	0,039747	ITGA3/ID1/ACVR1/ZYX/FOS/CDKN1C/KLF10/UBC	8
BP	GO:0009746	response to hexose	8/153	196/18493	0,00023	0,045132	0,039747	PTGS2/PIM3/RAB11FIP5/EGR1/KHK/RMI1/GLUL/HIF1A	8
BP	GO:0071559	response to transforming growth factor beta	9/153	249/18493	0,000233	0,045132	0,039747	ITGA3/ID1/ACVR1/ZYX/ARG1/FOS/CDKN1C/KLF10/UBC	9
BP	GO:0051017	actin filament bundle assembly	7/153	151/18493	0,000264	0,045132	0,039747	ID1/SORBS3/ZYX/RND3/MARCKS/RHOB/S100A10	7
BP	GO:0034284	response to monosaccharide	8/153	201/18493	0,000273	0,045132	0,039747	PTGS2/PIM3/RAB11FIP5/EGR1/KHK/RMI1/GLUL/HIF1A	8
BP	GO:0061572	actin filament bundle organization	7/153	155/18493	0,000309	0,045132	0,039747	ID1/SORBS3/ZYX/RND3/MARCKS/RHOB/S100A10	7
BP	GO:0021987	cerebral cortex development	6/153	111/18493	0,000322	0,045132	0,039747	TACC2/XAB2/SLC2A1/SLC38A2/HIF1A/FBXO45	6
BP	GO:0071214	cellular response to abiotic stimulus	10/153	319/18493	0,00033	0,045132	0,039747	HVCN1/PTGS2/TLR5/SLC2A1/RAB11FIP5/EGR1/SLC38A2/RHOB/BCL2L1/DDX3X	10
BP	GO:0104004	cellular response to environmental stimulus	10/153	319/18493	0,00033	0,045132	0,039747	HVCN1/PTGS2/TLR5/SLC2A1/RAB11FIP5/EGR1/SLC38A2/RHOB/BCL2L1/DDX3X	10
BP	GO:0071241	cellular response to inorganic substance	8/153	207/18493	0,000333	0,045132	0,039747	HVCN1/JUND/PTGS2/FOXO1/MMP3/FBP1/CDKN1B/FOS	8
BP	GO:0046677	response to antibiotic	10/153	323/18493	0,000365	0,047079	0,041461	ID1/AREG/FOXO1/ACTR2/CDKN1B/EGR1/ARG1/DUSP1/RHOB/BCL2L1	10
CC	GO:0005741	mitochondrial outer membrane	8/156	180/19659	9,69E-05	0,021329	0,019331	HADHB/RAB11FIP5/ARG1/MCL1/BCL2L1/PPP2R2B/DDX3X/UBC	8
CC	GO:0031968	organelle outer membrane	8/156	204/19659	0,000229	0,021329	0,019331	HADHB/RAB11FIP5/ARG1/MCL1/BCL2L1/PPP2R2B/DDX3X/UBC	8
CC	GO:0019867	outer membrane	8/156	206/19659	0,000245	0,021329	0,019331	HADHB/RAB11FIP5/ARG1/MCL1/BCL2L1/PPP2R2B/DDX3X/UBC	8
CC	GO:0005925	focal adhesion	11/156	402/19659	0,000375	0,021329	0,019331	ITGA3/AHNAK/SORBS3/ACTR2/RRAS2/ZYX/RND3/PPIA/MARCKS/RHOB/ACTB	11
CC	GO:0005924	cell-substrate adherens junction	11/156	405/19659	0,000399	0,021329	0,019331	ITGA3/AHNAK/SORBS3/ACTR2/RRAS2/ZYX/RND3/PPIA/MARCKS/RHOB/ACTB	11
CC	GO:0030055	cell-substrate junction	11/156	409/19659	0,000434	0,021329	0,019331	ITGA3/AHNAK/SORBS3/ACTR2/RRAS2/ZYX/RND3/PPIA/MARCKS/RHOB/ACTB	11

Supplementary Table 11: Candidate genes that were absent, overlooked, not consistently found as DE, or not found as DE at all in the studies used in IDA. They are taken from the 351 top Upregulated genes commonly identified by microarray and RNA-Seq analyses in regenerative vs. control comparison

Gene Symbol	Gene Name	Validated by qRT-PCR	PathCards (Human biological pathway unification)- SuperPathway Name : if > 10, the top 10 are shown
RBMXL1	RBMX Like 1		mRNA Splicing - Major Pathway
CCDC112	Coiled-Coil Domain Containing 112		N/A
TRIP13	Thyroid Hormone Receptor Interactor 13		N/A
PCDHGC3	Protocadherin Gamma Subfamily C, 3	Yes	N/A
SLC37A2	Solute Carrier Family 37 Member 2		N/A
CSE1L	Chromosome Segregation 1 Like		p53 pathway/Cell cycle_Spindle assembly and chromosome separation/Direct p53 effectors
POLR1C	RNA Polymerase I And III Subunit C		Assembly of RNA Polymerase-I Initiation Complex/Inhibition of Ribosome Biogenesis by p14(ARF)/RNA Polymerase I Promoter Escape/RNA Polymerase III Transcription Initiation/Cytosolic sensors of pathogen-associated DNA/ATP,ITP metabolism/RIG-I,MDA5 mediated induction of IFN-alpha/beta pathways/Chks in Checkpoint Regulation/Activated PKN1 stimulates transcription of AR (androgen receptor) regulated genes KLK2 and KLK3/Gene Expression
TMPO	Thymopoietin		Initiation of Nuclear Envelope Reformation/Mitotic Metaphase and Anaphase/Transport of the SLBP independent Mature mRNA/Apoptosis and Autophagy/Chromatin Regulation, Acetylation/Mitotic Prophase/DNA Damage/Cytoskeletal Signaling/Cell Cycle, Mitotic
IFNAR1	Interferon Alpha And Beta Receptor Subunit 1	Yes	Osteoclast Signaling/Peginterferon alpha-2a, Peginterferon alpha-2b Pathway (Hepatocyte), Pharmacodynamics/Downstream signaling in naive CD8+ T cells/Interferon type I signaling pathways/Immune response IFN alpha, beta signaling pathway/Osteoclast differentiation/Type I Interferon Signaling Pathways/Immune response Role of DAP12 receptors in NK cells/Necroptosis/Interferon gamma signaling
CTPS1	CTP Synthase	Yes	Synthesis and interconversion of nucleotide di- and triphosphates/superpathway of pyrimidine deoxyribonucleotides de novo biosynthesis/Metabolism of nucleotides/Metabolism
TNFAIP1	TNF Alpha Induced Protein 1		N/A
GART	Phosphoribosylglycinamide Formyltransferase		Antifolate resistance/One carbon pool by folate/purine nucleotides de novo biosynthesis/Selenium Micronutrient Network/Metabolism of nucleotides/Metabolism
ZRANB2	Zinc Finger RANBP2-Type Containing 2		N/A
EWSR1	EWS RNA Binding Protein 1		BARD1 signaling events/Translational Control/Transcriptional misregulation in cancer/Chromatin Regulation / Acetylation
CTTNBP2NL	CTTNBP2 N-Terminal Like		N/A
PRPF39	Pre-MRNA Processing Factor 3		N/A
PGGT1B	Protein Geranylgeranyltransferase Type I Subunit Beta		G-protein signaling_Rap2B regulation pathway/G-protein signaling_Rap1B regulation pathway/G-protein signaling_RhoB regulation pathway/G-protein signaling_Rac2 regulation pathway/Cytoskeleton remodeling_RalA regulation pathway/G-protein signaling_Rap1A regulation pathway/G-protein signaling_Regulation of RAC1 activity/G-protein signaling_RhoA regulation pathway
ZNF862	Zinc Finger Protein 862		N/A
METTL2A	Methyltransferase Like 2A	Yes	N/A
USP1	Ubiquitin Specific Peptidase 1		miR-517 relationship with ARCN1 and USP1/ Fanconi anemia pathway/ DNA damage_ATM/ATR regulation of G1/S checkpoint/Translesion synthesis by Y family DNA polymerases bypasses lesions on DNA template/Telomere C-strand (Lagging Strand) Synthesis/Ubiquitin-Proteasome Dependent Proteolysis/DNA Damage/ DNA Double-Strand Break Repair
CLNS1A	Chloride Nucleotide-Sensitive Channel 1A		Hepatic ABC Transporters/Transport of the SLBP independent Mature mRNA/RNA transport/Neuropathic Pain-Signaling in Dorsal Horn Neurons/Activation of cAMP-Dependent PKA/Gene Expression
MCM3AP	Minichromosome Maintenance Complex Component 3 Associated Protein		N/A
UBE2I	Ubiquitin Conjugating Enzyme E2 I		SUMOylation/Sumoylation by RanBP2 regulates transcriptional repression/Regulation of cytoplasmic and nuclear SMAD2/3 signaling/HIF Repressor Pathways/Signaling events mediated by HDAC Class II/Transcriptional regulation by the AP-2 (TFAP2) family of transcription factors/Jak,STAT Signaling Pathway Intracellular Regulation/Proteolysis Putative SUMO-1 pathway/Development_Glucocorticoid receptor signaling/Coregulation of Androgen receptor activity
